# SPNOA: An Improved Nutcracker Optimizer Based on Specular Reflection Learning and Probability-Driven Hybrid Mutation Strategy for Global Optimization

**DOI:** 10.3390/biomimetics11100764

**Published:** 2026-10-21

**Authors:** Xingbo Yao, Changqing Fang

**Affiliations:** 1 Department of Art and Design, Xi’an University of Technology, Xi’an 710054, China; 2 Department of International Engineering, Xi’an University of Technology, Xi’an 710054, China

**Keywords:** nutcracker optimizer, the specular reflection learning strategy, the nonlinear convergence factor, the diversity attack strategy, the probability-driven hybrid mutation strategy, engineering optimization problems

## Abstract

The nutcracker optimization algorithm (NOA) is a novel swarm intelligence algorithm, inspired by the different natural behaviors of nutcrackers during two periods. Compared with many classical algorithms, NOA performs competitively on various test suites and optimization problems. NOA still suffers from local-optimum entrapment, an imbalance between exploration and exploitation, and insufficient convergence accuracy. To address these deficiencies, this paper proposes a multi-strategy enhanced NOA, termed SPNOA. The proposed method incorporates specular reflection learning, a nonlinear convergence factor, a diversity attack strategy, and a probability-driven hybrid mutation strategy. First, the specular reflection learning strategy is employed to enhance the diversity of the initial population. Second, the nonlinear convergence factor is introduced to balance the exploration and exploitation of SPNOA. Then, to augment the exploitation ability of the algorithm and improve its convergence accuracy, the diversity attack strategy is used to replace the storage phase in the foraging and storage strategy. Finally, the probability-driven hybrid mutation strategy is used before the end of each iteration, which helps SPNOA to break free from the local optimum. Subsequently, NOA, the newly proposed algorithm, the high-performance improved algorithm, and the CEC competition-winning algorithms are chosen as the comparison algorithms. Experiments are conducted across various dimensions of the CEC2020 test suite to assess the performance of SPNOA. The outcomes demonstrate that the proposed SPNOA exhibits superior convergence accuracy and robustness. Finally, 27 real-world engineering problems in different domains are selected to validate the ability of SPNOA to handle realistic optimization problems. The experimental results indicate that SPNOA retains its competitive edge in tackling complex, real-world optimization problems with diverse dimensions.

## 1. Introduction

An optimization problem entails selecting the optimal solution within a given feasible set to either maximize or minimize a target value [1]. Almost all scientific and technological fields involve optimization problems. With the continuous advancement of society, the intricacy of real-world optimization challenges is escalating. Since these optimization problems usually exhibit nonlinear, multimodal, nondifferentiable, and high-dimensional characteristics [2], traditional optimization algorithms cannot solve them efficiently. This is because traditional optimization algorithms often have high time complexity and may require long solution times and encounter difficulties when solving large-scale practical engineering problems. The field received widespread attention when researchers discovered that the metaheuristic algorithm (MA) could efficiently tackle these complex optimization problems.

MAs have the advantages of simplicity, flexibility, and not requiring derivation mechanisms. MAs draw their primary inspiration from natural phenomena or mechanisms. The principles underlying these algorithms are relatively simple but highly practical for different optimization problems. When the problem to be solved changes, MAs can be used to solve the new problem with only minor adjustments, thereby obtaining a superior outcome. Based on the different types of inspiration for algorithms, MAs are divided into evolutionary algorithms (EAs), physics-based or mathematical principles-based algorithms, swarm intelligence algorithms (SIs), and human-based algorithms [3].

EAs are inspired by the process of biological evolution. Classical examples of EAs include the genetic algorithm (GA) [4], evolutionary strategy (ES) [5], and differential evolution (DE) [6], among others, of which GA is the most representative. New biological evolution-based algorithms are also continuously proposed by researchers, such as the earthworm optimization algorithm [7] and the tree-seed algorithm [8]. Physics-based or mathematical-based algorithms are inspired by physical laws or mathematical theorems. Some representative examples include simulated annealing (SA) [9], which is based on the principles of solid annealing, sine cosine algorithm (SCA) [10], which utilizes the sine cosine mathematical model, exponential-trigonometric optimization (ETO) [11], obtained through a complex combination of exponential function and trigonometric function, and symmetric projection optimizer algorithm (SPO) [12], which is based on Fourier series fundamental wave fitting. SIs are inspired by the foraging survival strategies of natural organisms. The most iconic example is particle swarm optimization (PSO) [13]. Such algorithms draw inspiration from an array of sources, so new swarm intelligence algorithms continue to be proposed, including the gray wolf optimizer (GWO) [14], superb fairy-wren optimization algorithm (SFOA) [15], harris hawks optimization (HHO) [16], Marine Predators Algorithm (MPA) [17], genghis khan shark optimizer (GKSO) [18], and artificial rabbit optimization (ARO) [19]. The last type of classification is human-based algorithms. Such algorithms are mainly inspired by human thinking and behavioral styles. Examples include teaching-learning-based optimization (TLBO) [20], dream optimization algorithm (DOA) [21], and alpine Skiing optimization (ASO) [22].

Due to the high applicability of MAs, complex optimization problems in different domains can be solved using MAs. For example, an adaptive quadratic interpolation optimization algorithm has been applied to six popular engineering design problems [23]. An improved glider snake algorithm has been used to design vehicle suspension component design [24]. A multi-strategy improved wide-kissing dolphin algorithm for constrained path planning for unmanned aerial vehicles to identify optimal feasible paths [25]. An improved multi-objective PSO to optimize the intrinsic frequency and mass of gradient plates with different loading configurations for the design of bi-directional functionally graded plates with variable thickness [26].

Recent advances in evolutionary optimization have explored richer search-enhancement mechanisms. Song et al. [27] proposed a learning-assisted search-path reconstruction evolution strategy, which employs a fully connected neural network to reconstruct continuous search paths from discrete samples collected during the evolutionary process. Fu et al. [28] developed a spatial information sampling algorithm with an adaptive distributed population structure, in which the population is divided into multiple subpopulations and coordinated through multilayer information interaction. These studies demonstrate the potential of learning-assisted search-path reconstruction and adaptive population organization for improving exploration and exploitation.

According to the No Free Lunch (NFL) [29] theorem, we know that no algorithm is universally effective for every optimization problem, which means that each MA will have optimization problems that it is good at handling and optimization problems that it cannot solve efficiently. This theory drives scholars in the field of intelligent optimization to continuously invent new algorithms or refine existing ones to fit a more diverse set of optimization problem types. Exploration and exploitation are the two most significant components of MAs. In the exploration phase, an MA accesses invisible neighbors within the feasible domain, thereby significantly improving the efficiency of locating the global optimum. During the exploitation phase, the MA searches the area surrounding the previously identified optimum in an attempt to refine and obtain the global optimal solution [30]. After successfully balancing these two important phases for MAs, algorithms can attain optimal performance for specific optimization tasks. Nutcracker optimizer (NOA) [31] is an innovative MA inspired by the different natural behaviors of nutcrackers during two periods. Like other MAs, the original NOA suffers from the drawbacks of unbalanced exploration and exploitation, and is prone to premature convergence and limited solution precision. Therefore, this paper introduces an enhanced NOA, termed SPNOA. Unlike these approaches, SPNOA does not rely on learning-assisted search-path reconstruction or an adaptive distributed population architecture. Instead, it retains the two-behavior framework of NOA and improves its initialization, exploration-exploitation control, population diversity, and local-optimum avoidance through four mechanisms. The main idea of SPNOA is as follows:

First, SPNOA employs the specular reflection learning strategy for population initialization to achieve a more favorable starting population and accelerate the convergence process. Second, to balance exploitation and exploration, different nonlinear convergence factors are introduced to replace the exchange of two important strategies in the original algorithm and the exchange of two phases in the foraging and storage strategy, respectively. Meanwhile, the diversity attack strategy is incorporated to strengthen the exploitation capability. Lastly, the probability-driven hybrid mutation strategy is introduced to augment its capacity to evade local optima.

The principal contributions of this paper are as follow:

• An improved algorithm termed SPNOA is proposed based on the specular reflection learning strategy, nonlinear convergence factor, diversity attack strategy, and probability-driven hybrid mutation strategy.
• On the CEC2020 test suite, SPNOA is compared with 16 algorithms through a large number of experiments. The experimental results are analyzed in depth to confirm the advantages of SPNOA over other compared algorithms.
• SPNOA’s effectiveness in addressing complex optimization tasks is validated through 27 real-world engineering problems.

The framework of this study is as follows: Section 2 reviews the related work on NOA improvements and applications. Section 3 provides a comprehensive account of NOA. Section 4 proposes an improved NOA, along with detailed descriptions of the four strategies utilized. Section 5 begins with a sensitivity analysis of the three important parameters and then analyzes the experimental data for the CEC2020 test suite. Section 6 applies SPNOA and comparative algorithms to a total of 27 real-world engineering problems in five areas and analyzes the results. Section 7 presents the conclusions and discusses future work.

## 2. Related Work

Improvements to NOA generally aim to strengthen population coverage and diversity throughout the search process. Common strategies include chaotic mapping, high-quality population initialization, and perturbation-based candidate generation. For example, logistic-tent chaotic mapping [32] is adopted to distribute the initial population. Good point set initialization [33] and hybrid initialization [34] yield more uniformly distributed or higher-quality initial candidates. In addition, Cauchy perturbation [35], t-distribution perturbation [33], and chaotic lens-imaging opposition-based learning [34] generate supplementary candidates and assist the algorithm in escaping stagnant regions. Fitness-based population decomposition and Q-learning strategy selection [36] are used to balance exploration and exploitation. Differential evolution search [37], migration and exhaustive methods [38] are used to enhance the algorithm’s exploitation capability.

Sun et al. [32] constructed a multi-strategy INOA by combining logistic-tent chaotic mapping, dynamic fitness-distance balance selection, and other strategies. The method was evaluated on benchmark functions and then used for PCCP wire-break signal analysis and dam HST prediction. Luo et al. [34] developed INOA-SQP for multi-AUV path planning. Hybrid initialization improved solution quality, dynamic hierarchical acceptance controlled candidate selection, elitist SQP enhanced local search, and chaotic dynamic lens-imaging opposition learning helped escape late-stage local optima. Li et al. [36] developed RLNOA by dividing the population into exploration and exploitation subpopulations according to fitness. Random opposition-based learning was used to generate alternative candidates, while Q-learning selected exploitation actions adaptively. The method improved the coordination between global exploration and local exploitation. Dahou et al. [39] embedded an enhanced NOA in a multitask Transformer framework for Arabic fake-news detection. Across Arabic social-media datasets, the framework achieved about 87% accuracy for binary classification. Mohamed et al. [40] combined NOA with Chaos Game Optimization and CrossViT feature extraction for breast-image classification. The modified optimizer was tested on the CEC2022 test suite and several image classification datasets. Zhang et al. [41] integrated random opposition learning, non-monopolized search, and a phased Marine Predators Algorithm into NOA, and used the resulting INOA to optimize the parameters of a hybrid kernel extreme learning machine. The INOA-HKELM model improved the accuracy and stability of projectile aerodynamic parameter identification. Chen et al. [42] developed HNOA by adding Tent-map initialization and perturbation. In a garbage-collection traffic network, the HNOA-based green vehicle-routing plan reduced average carbon emissions by up to 7.28%. Dou et al. [43] incorporated genetic crossover into foraging, mutation-driven cache recovery, and adaptive boundary constraints to form GNOA. The method generated feasible, collision-free, and energy-aware trajectories for multiple UAVs in complex three-dimensional terrain. Ji et al. [44] proposed a Nutcracker Genetic Algorithm for task scheduling in UAV swarm mobile edge computing with adaptive caching. The cache-and-recovery mechanism of NOA was retained and reinforced with dedicated crossover and mutation operators, reducing task-completion time by up to 42.8%.

Furthermore, NOA and its variants have been widely applied across multiple engineering and scientific domains. In energy systems, they address parameter estimation, scheduling, forecasting, and control tasks, including wind-solar integrated energy dispatch [45], photovoltaic power forecasting [46], and transient frequency regulation [47].

Combined with predictive models, NOA variants perform feature selection and parameter tuning for tasks such as groundwater mapping [48] and lung diagnosis [49]. Table 1 summarizes the research conducted in the aforementioned literature.

These studies demonstrate that NOA’s search process has been strengthened through chaotic mapping, high-quality population initialization, and perturbation-based candidate generation. The improved algorithm has also been applied to solve optimization problems in various fields. Despite these advancements, these variants of NOA still face issues such as premature convergence or an imbalance between exploration and exploitation processes. In this context, this paper proposes an improved nutcracker optimizer based on specular reflection learning and a probability-driven hybrid mutation strategy (SPNOA), aiming to further enhance the optimization capability of NOA.

## 3. Nutcracker Optimizer

NOA is inspired by two different patterns of behavior that the nutcracker exhibits at different times of the year. In summer and fall, nutcrackers seek out seeds and store good seeds in suitable storage points, a behavior summarized as the foraging and storage strategy. In winter and spring, nutcrackers forage for hidden storage points that they have marked at various angles, utilizing distinct markings as reference points to locate them. When nutcrackers fail to find seeds based on reference points, they search randomly in the space to find seeds, a behavior summarized as the cache search and recovery strategy. Both behaviors are equally important and therefore have the same likelihood of occurring. This section introduces the principles and models of NOA. For ease of reference, the main variables in the manuscript are presented in Table 2.

### 3.1. Initialization

In NOA, the initial population is generated randomly. Equation (1) presents the formulation for the random initialization process.

(1) $$X_{i,j}^{\mathrm{iter}}=L_{j}+(U_{j}-\;L_{j})\;\times \;\mathrm{RM},$$

where $X_{i,j}^{\mathrm{iter}}$ denotes the coordinates of the *j*th dimension for the *i*th nutcracker individual in the *iter*th iteration. *L_j_* denotes the lower bound for the *j*th dimension, and *U_j_* represents the upper bound of that dimension. *RM* denotes a random number uniformly distributed within [0,1]. Each nutcracker individual can be viewed as a feasible solution.

### 3.2. Foraging and Storage Strategy

This strategy consists of two primary phases, foraging and storage, both of which are elaborated upon in detail in this section.

#### 3.2.1. Foraging Phase

The foraging phase is also referred to as exploration phase 1. At this stage, nutcrackers start to adopt the initial position/food location generated by Equation (1) in the search space (collection area). Each nutcracker examines a cone at the initial location, which contains seeds. If nutcrackers find good-quality seeds in this cone, they transport the seeds to the storage area and bury them in a storage point. If nutcrackers do not find suitable seeds in the cone, they will then seek out another cone in a pine or another tree. Equation (2) is the mathematical formulation of the foraging phase.

(2) Xi,jiter(new)=Xi,jiter, if τ1<τ2,Xtp1i,tp1jiter, otherwise,

(3) $$X_{\mathrm{tp}1i,\mathrm{tp}1j}^{\mathrm{iter}}=\left\{\begin{array}{l} X_{m,j}^{\mathrm{iter}}+\gamma \times (X_{A,j}^{\mathrm{iter}}-X_{B,j}^{\mathrm{iter}})+\mu \times (r^{2}\times U_{j}-L_{j}),\mathrm{if t}\leq T_{\max}/2,\\ X_{C,j}^{\mathrm{iter}}+\mu \times (X_{A,j}^{\mathrm{iter}}-X_{B,j}^{\mathrm{iter}})+\mu \times (r_{1}<\delta )\times (r^{2}\times U_{j}-L_{j}),\mathrm{otherwise}, \end{array}\right.$$

where $X_{i}^{\mathrm{iter}(\mathrm{new})}$ is the new location for the *i*th nutcracker individual. $X_{m,j}^{\mathrm{iter}}$ denotes the average of all solutions in the *j*th dimension. *τ*_1_, *τ*_2_, *r*, and *r*_1_ are random numbers within [0,1]. *A*, *B*, *C* denote the indexes of nutcrackers randomly chosen and which are not identical to each other. *γ* represents a random number generated through Lévy flights. *t* is the current number of function calculations, while *T_max_* is the maximum allowed. *δ* denotes the parameter that controls the updating within the current dimension’s upper and lower bounds, which was determined to take the value of 0.05 through sensitivity analysis in the original NOA article. The *μ* generation method is randomly selected from a uniform distribution (*τ*_3_), a standard normal distribution (*τ*_4_), and a Lévy flight (*τ*_5_), as shown in Equation (4).

(4) μ=τ3, if r1<r2,τ4, if r2<r3,τ5, if r1<r3,

where *r*_2_ and *r*_3_ are random numbers.

#### 3.2.2. Storage Phase

The storage phase is also known as exploitation phase 1. Nutcrackers move the seeds they have collected to a temporary storage location (Storage Area) away from the collection area. That is, at this time, nutcrackers exploit the seeds of pine trees and store them. Equation (5) is the mathematical formulation of the storage phase.

(5) Xiiter(new)=Xiiter+ μ ×(Xbestiter−Xiiter)×λ+r1×(XAiter−XBiter), if τ1<τ2,Xbestiter+ μ ×(XAiter−XBiter), if τ1<τ3,Xbestiter× l, otherwise,

where $X_{\mathrm{best}}^{\mathrm{iter}}$ denotes the optimal solution found so far. *λ* is a number generated using Lévy flights. *l* is the factor that decreases linearly in the interval [0,1] during the storage phase.

The foraging phase is exchanged with the storage phase in accordance with Equation (6).

(6) $$X_{i}^{\mathrm{iter}(\mathrm{new})}=\left\{\begin{array}{c} \mathrm{Equation}\;(2),\mathrm{if \varphi}<P_{a_{1}},\\ \mathrm{Equation}\;(5),\mathrm{otherwise}, \end{array}\right.$$

where *φ* represents a random within [0,1]. $P_{a_{1}}$ represents a probability value that diminishes linearly from 1 to 0 as the number of function calculations increases.

### 3.3. Cache Search and Recovery Strategy

This strategy consists of two primary phases, cache search and recovery, both of which are thoroughly elaborated in this section.

#### 3.3.1. Cache Search Phase

The cache search phase is also known as exploration phase 2. In this phase, the nutcrackers localize storage points through a spatial memory strategy, but nutcrackers may use multiple different objects as markers for a single storage point. When building the mathematical model, NOA assumes that each storage point only has two markers, and defines these two markers as reference points (RPs). The two RPs for each nutcracker/storage point can be present in a matrix, as shown in Equation (7).

(7) $$\begin{array}{c} \mathrm{RPs}=\left[\begin{array}{c} {\mathrm{RP}}_{1,1}^{\mathrm{iter}}{\mathrm{RP}}_{1,2}^{\mathrm{iter}}\\ \vdots \vdots \\ \begin{array}{c} {\mathrm{RP}}_{i,1}^{\mathrm{iter}}{\mathrm{RP}}_{i,2}^{\mathrm{iter}}\\ \vdots \vdots \end{array}\\ {\mathrm{RP}}_{N,1}^{\mathrm{iter}}{\mathrm{RP}}_{N,2}^{\mathrm{iter}} \end{array}\right], \end{array}$$

where ${\mathrm{RP}}_{i,1}^{\mathrm{iter}}$ and ${\mathrm{RP}}_{i,2}^{\mathrm{iter}}$ are the two RPs of the storage point $X_{i}^{\mathrm{iter}}$ of the *i*th nutcracker. Indexes of the rows of the matrix denote the indexes of the nutcracker individuals/storage points, and the indexes of the columns of the matrix denote the indexes of the RPs.

To improve the exploration operation performed by the nutcracker in searching for storage points for seeds, the first and second RPs were generated using different equations, as indicated by Equations (8) and (9), respectively.

(8) $${\mathrm{RP}}_{i,1}^{\mathrm{iter}}=\left\{\begin{array}{l} X_{i}^{\mathrm{iter}}+\alpha \times \cos(\theta )\times (X_{A}^{\mathrm{iter}}-X_{B}^{\mathrm{iter}})+\alpha \times \mathrm{RP},\mathrm{if \theta}=\pi /2,\\ X_{i}^{\mathrm{iter}}+\alpha \times \cos(\theta )\times (X_{A}^{\mathrm{iter}}-X_{B}^{\mathrm{iter}}),\mathrm{otherwise}, \end{array}\right.$$

(9) $${\mathrm{RP}}_{i,2}^{\mathrm{iter}}=\left\{\begin{array}{l} X_{i}^{\mathrm{iter}}+(\alpha \times \cos(\theta )\times ((U-L)\times \tau_{3}+L)+\alpha \times \mathrm{RP})\times U_{2},\mathrm{if \theta}=\pi /2,\\ X_{i}^{\mathrm{iter}}+\alpha \times \cos(\theta )\times ((U-L)\times \tau_{3}+L)\times U_{2},\mathrm{otherwise}, \end{array}\right.$$

(10) U2=1, if r2<Prp,0, otherwise,

where ${\mathrm{RP}}_{i,1}^{\mathrm{iter}}$ and ${\mathrm{RP}}_{i,2}^{\mathrm{iter}}$ are the first and second columns of the *i*th row of the matrix defined in Equation (7), respectively, representing two RPs of the *i*th nutcracker’s current position/storage point. *θ* represents the viewpoint of the nutcrackers and is randomly generated within [0, π]. *RP* is the randomly selected position. *P_rp_* is a probability value used to regulate the inclination for exploring other regions, and it was determined that this value should be set to 0.2 through a sensitivity analysis conducted in the original NOA paper. To ensure that NOA can achieve periodic convergence, the formula for *α* is displayed in Equation (11).

(11) α =(1−tTmax)2×tTmax, if r1<r2,(tTmax)2t, otherwise,

In NOA, each nutcracker employs an exploration mechanism that searches for potential regions that may hide an optimal solution. As iterations continue, the algorithm will use appropriate RPs to explore and exploit the area around the storage point. The calculation for updating the nutcrackers’ position is presented in Equation (12).

(12) $$X_{i}^{\mathrm{iter}(\mathrm{new})}=\left\{\begin{array}{l} X_{i}^{\mathrm{iter}},\mathrm{if f}(X_{i}^{\mathrm{iter}})<f({\mathrm{RP}}_{i,1}^{\mathrm{iter}}),\\ {\mathrm{RP}}_{i,1}^{\mathrm{iter}},\mathrm{otherwise}, \end{array}\right.$$

where *f*() denotes the fitness function, which is used to determine the fitness value. Equation (12) serves to guide the NOA to explore and exploit the promising regions around ${\mathrm{RP}}_{i,1}^{\mathrm{iter}}$. If no satisfying region is found around ${\mathrm{RP}}_{i,1}^{\mathrm{iter}}$, it will be shifted to the promising region around ${\mathrm{RP}}_{i,2}^{\mathrm{iter}}$, whose update formula is shown in Equation (13).

(13) $$X_{i}^{\mathrm{iter}(\mathrm{new})}=\left\{\begin{array}{l} X_{i}^{\mathrm{iter}},\mathrm{if f}(X_{i}^{\mathrm{iter}})<f({\mathrm{RP}}_{i,2}^{\mathrm{iter}}),\\ {\mathrm{RP}}_{i,2}^{\mathrm{iter}},\mathrm{otherwise}. \end{array}\right.$$

The balance between the exploration behaviors performed by the RPs is achieved by Equation (14).

(14) $$X_{i}^{\mathrm{iter}(\mathrm{new})}=\left\{\begin{array}{l} \mathrm{Equation}\;(12),\mathrm{if f}(\mathrm{Equation}\;(12))<f(\mathrm{Equation}\;(13)),\\ \mathrm{Equation}\;(13),\mathrm{otherwise}. \end{array}\right.$$

#### 3.3.2. Recovery Phase

The recovery phase is also known as exploitation phase 2. In this phase, nutcrackers remove seeds from storage points. As the nutcracker uses RPs to locate the storage point, two scenarios can occur. The first scenario is that the nutcracker uses the first RPs to locate the storage point, at which point there are again two possible outcomes: the seed in the storage point either exists or does not exist. Equation (15) is the formula for the first scenario of the recovery phase.

(15) Xi,jiter(new)=Xi,jiter, if τ3<τ4,Xi,jiter+r1×(Xbest,jiter−Xi,jiter)+r2×(RPi,1iter−XC,jiter), otherwise.

The second scenario is that nutcrackers cannot locate the storage point using the first RPs, in which case they use the second RPs to find the storage point. The formula for the second scenario is shown in Equation (16).

(16) Xi,jiter(new)=Xi,jiter, if τ5<τ6,Xi,jiter+r1×(Xbest,jiter−Xi,jiter)+r2×(RPi,2iter−XC,jiter), otherwise,

where *τ*_6_ is a random number. The simulation of nutcracker behavior during the recovery phase is shown in Equation (17).

(17) Xiiter(new)=Equation (15), if τ7<τ8,Equation (16), otherwise,

where *τ*_7_ and *τ*_8_ are random numbers within [0,1].

The shift between the cache search and recovery phases is determined by Equation (18).

(18) $$X_{i}^{\mathrm{iter}(\mathrm{new})}=\left\{\begin{array}{c} \mathrm{Equation}(17),\mathrm{if \phi}<P_{a_{2}},\\ \mathrm{Equation}(14),\mathrm{otherwise}, \end{array}\right.$$

where *ϕ* represents a random value within [0,1]. $P_{a_{2}}$ denotes a probability value, which was set to 0.2 based on the sensitivity analysis conducted in the original NOA article.

Finally, both the original and updated solutions of the nutcrackers are assessed based on the fitness function. When the fitness value of the updated solution is superior to the original, the nutcracker’s current position will be modified. Conversely, the nutcracker keeps the current position unchanged. The mechanism is summarized in Equation (19). The pseudo-code of NOA is presented in Algorithm 1.

(19) $$X_{i}^{\mathrm{iter}(\mathrm{new})}=\left\{\begin{array}{l} X_{i}^{\mathrm{iter}(\mathrm{new})},\mathrm{if f}(X_{i}^{\mathrm{iter}(\mathrm{new})})<f(X_{i}^{\mathrm{iter}}),\\ X_{i}^{\mathrm{iter}},\mathrm{otherwise}, \end{array}\right.$$

**Algorithm 1:** Pseudo-code for NOA**Input**: *N*(Population size), *dim*(Population dimension), *U*(Upper limit), *L*(Lower limit), *t*(Current number of function calculations), *T_max_*(Maximum number of function calculations), *iter*(Current number of iteration loops).**Output**: The best Nutcracker *X_best_* and its corresponding fitness.Initialization of nutcracker/solution using Equation (1).Calculate each solution of the population and select the optimal one from it.*t* = *N*; *iter* = 1.**while** *t* < *T_max_*
Generate random numbers *σ*_1_ and *σ*_2_.
**If** *σ*_1_ < *σ*_2_// Foraging and storage strategy //

Generate random number *φ*.

**for** *i* = 1: *N*


**If** $\varphi <P_{a_{1}}$/ Exploration phase 1/



Update $X_{i}^{\mathrm{iter}}$ using Equation (2) and Equation (19).


**else**/Exploitation phase 1/



Update $X_{i}^{\mathrm{iter}}$ using Equation (5) and Equation (19).


**end if**


Current number of function calculations *t* = *t* + 1.

**end for**
**else**//Cache-search and recovery strategy//

Generate RP matrix using Equation (8) and Equation (9).

Generate random number *ϕ*.

**for** *i* = 1: *N*


**If** $\phi >P_{a_{2}}$/Exploration phase 2/



Update $X_{i}^{\mathrm{iter}}$ using Equation (14) and Equation (19).



Current number of function calculations *t* = *t* + 2.


**else**/ Exploitation phase 2/



Update $X_{i}^{\mathrm{iter}}$ using Equation (17) and Equation (19).


**end if**


Current number of function calculations *t* = *t* + 1.

**end for**
**end if**
Current number of iteration loops *iter* = *iter* + 1.**end while**

## 4. Improved Nutcracker Optimizer

Although the original NOA has strong performance, it also suffers from problems such as low convergence accuracy and uneven exploration and exploitation. Thus, this section introduces the enhanced variant of NOA, designated as SPNOA. SPNOA combines the specular reflection learning strategy, the nonlinear convergence factor, the diversity attack strategy, and the probability-driven hybrid mutation strategy, comprehensively enhancing NOA’s ability to identify the optimum.

### 4.1. Specular Reflection Learning Strategy

In the population initialization phase, good initialization results accelerate convergence, while poor initialization results increase the time required for the algorithm to converge. NOA uses the random initialization approach, which can generate an initial population with no relevant information to the optimization problem, but the random generation method will have low stability and uneven distribution. Hence, to bolster NOA’s global search capability and accelerate its convergence, this paper introduces the specular reflection learning strategy (SRL) [50].

**Figure 1:**
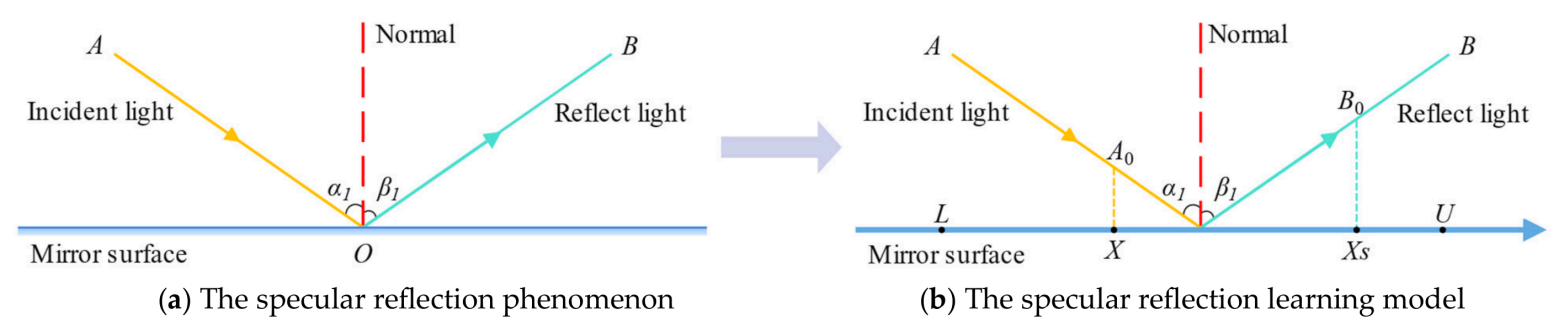
Diagram of the specular reflection learning principle.

The phenomenon of specular reflection is very common in real life. The underlying principle is that when light strikes the smooth surface of an object, the light will be reflected in one direction parallel to it. Figure 1a depicts the phenomenon of specular reflection, with *Ao* being the incident light and *oB* being the reflected light. The incident angle, denoted as *α*_1_, is the angle between the incident ray *Ao* and the normal. The reflection angle, denoted as *β*_1_, is the angle between the reflected ray *oB* and the normal. As per the law of reflection, *α*_1_ = *β*_1_.

In order to build a mathematical model of the specular reflection phenomenon, as shown in Figure 1b, we specify that the direction of the angle of incidence is positive. The possible solutions will only occur within the interval [*L*, *U*], with *O* = (*X*_0_,0) as the midpoint of the interval. *X* = (*a*,0) stands for a random variable in the given interval, and (*b*,0) represents the mirror solution *Xs* corresponding to the original solution *X*.

At this point, the tangent functions of the angles of incidence and reflection are Equations (20) and (21), respectively.

(20) $$\tan\alpha_{1}=\frac{X_{0}-a}{A_{0}},$$

(21) $$\tan\beta_{1}=\frac{b-X_{0}}{B_{0}}.$$

As dictated by the reflection law, *α*_1_ = *β*_1_. Therefore, the corresponding sinusoidal values of these two angles are also equal, and the model derived from this theory is shown in Equation (22). This can be simplified as shown in Equation (23).

(22) $$\frac{X_{0}-a}{A_{0}}=\frac{b-X_{0}}{B_{0}},$$

(23) $$b=\frac{B_{0}\times (X_{0}-a)}{A_{0}}+X_{0}.$$

To further simplify Equation (23), let *B*_0_ = *ω* × *A*_0_ and *ω* be positive real numbers to obtain Equation (24).

(24) $$\begin{array}{ll} b & =\omega \times (X_{0}-a)+X_{0}\\ & =(\omega +1)\times X_{0}-\omega \times a\\ & =0.5\times (\omega +1)\times (L+U)-\omega \times a, \end{array}$$

where *ω* is expressed as shown in Equation (25).

(25) ω=1+R0× ξ, if κ1>κ2,1−R0× ξ, otherwise,

where *R*_0_ is the neighborhood radius and *ξ* is the elasticity factor, both of which take values in the [0,1] interval, and *κ*_1_ and *κ*_2_ are random numbers in [0,1]. According to the established model of specular reflection learning, Equation (26) is the updated equation for the mirror solution.

(26) $${\mathrm{Xs}}_{i}^{\mathrm{iter}}=0.5\;\times \;(\omega +1)\;\times \;(L_{i}+U_{i})\;-\;\omega \;\times \;X_{i}^{\mathrm{iter}},$$

where ${\mathrm{Xs}}_{i}^{\mathrm{iter}}$ is the mirror solution for the *i*th nutcracker during the current *iter*th iteration.

### 4.2. Nonlinear Convergence Factor

In NOA, the two main strategies are of equal importance. Both strategies encompass the stages of global exploration and local exploitation, and therefore share an equivalent likelihood of being executed. Within the foraging and storage strategy, the probability value of transitioning between exploration and exploitation linearly decreases from 1 to 0 as the current number of function calculations increases, as described in Equation (6). However, for the cache search and recovery strategy, Equation (18) reveals that the probability of transitioning between the exploration and exploitation phases is consistently 0.2. This means that this strategy will be more biased towards the global exploration phase. To harness the powerful exploration capability of the cache search and recovery strategy effectively, the probabilities of the execution of the two strategies are adjusted as shown in Equation (27).

(27) S1, if 0.5<R1,S2, otherwise,

(28) $$R_{1}=0.2+0.6\times {\left(\frac{t}{T_{\max}}\right)}^{2},$$

where *S*1 denotes the foraging and storage strategy. *S*2 denotes the cache search and recovery strategy. *R*_1_ denotes a probability value that determines when the two strategies are exchanged. At this point, the algorithm will prefer to choose *S*2 to fully utilize its powerful global exploration ability.

In the foraging storage strategy, the convergence factor *Pa*_1_ decreases linearly from 1 to 0. However, the linear decrease in *Pa*_1_ does not accurately capture the complexity of the search process. More importantly, after incorporating the first nonlinear convergence factor, the algorithm will execute this strategy more often in the late iterations, when it is especially important to determine whether the exploitation capacity of the strategy is strong. Hence, to achieve a profound equilibrium between exploration and exploitation, *Pa*_1_ is adjusted to decrease nonlinearly from 0.8 to 0.2, as depicted in Equation (29).

(29) $${\mathrm{Pa}}_{1}=0.2+0.6\times {\left(\frac{T_{\max}-t}{T_{\max}}\right)}^{4},$$

**Figure 2:**
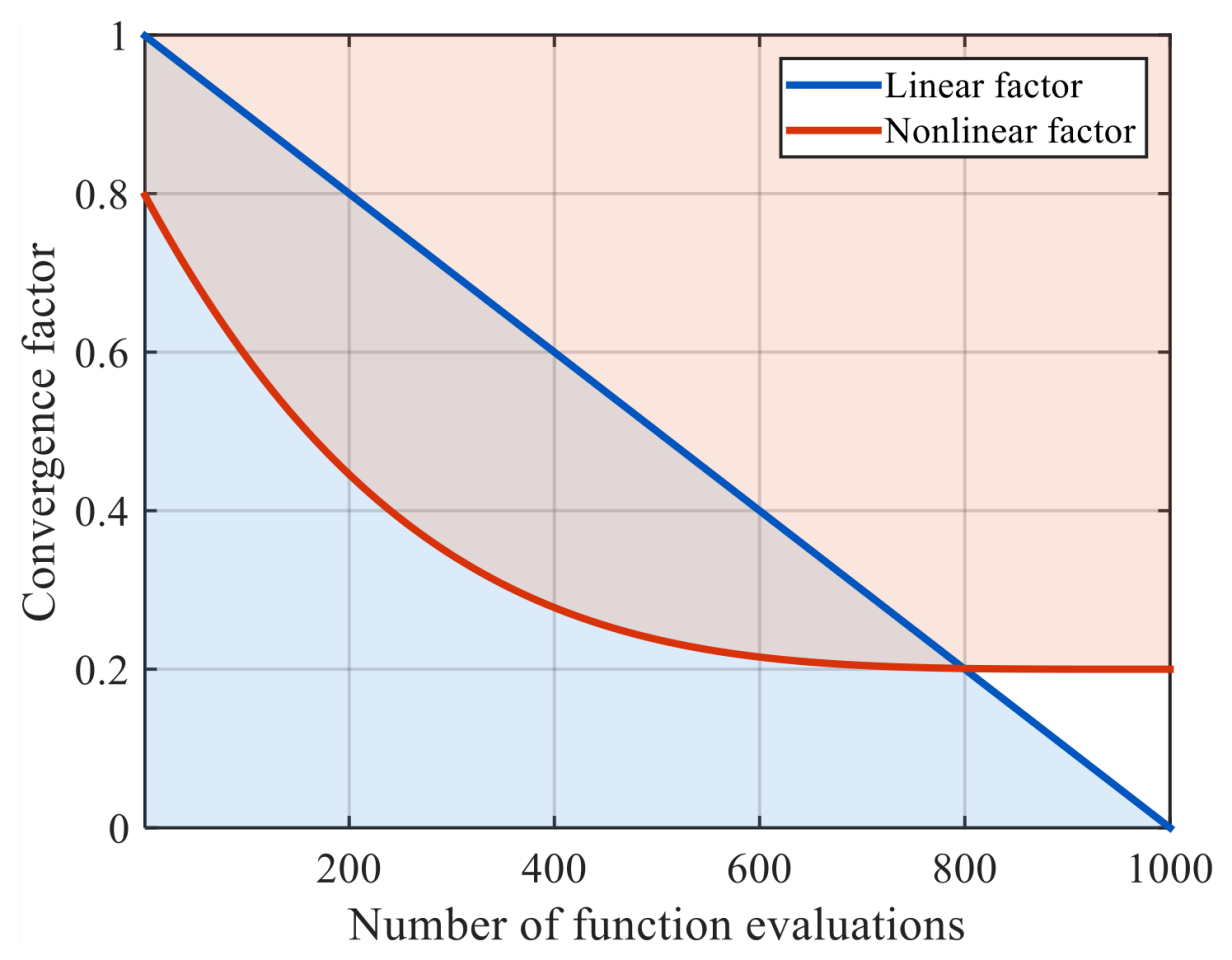
Comparison of linear and fourth-power nonlinear convergence factors.

Compared with the original linear factor, the nonlinear convergence factor in Equation (29) decreases more rapidly during the early and middle stages of the search while maintaining a lower bound of 0.2 in the later stage. As shown in Figure 2, the shaded regions represent the ranges in which the corresponding factor can activate the associated search operation. This nonlinear schedule provides a different temporal allocation of the search operations and avoids a complete loss of their influence in the final search stage.

In SPNOA, the execution of the two important strategies is determined by the nonlinear convergence factor *R*_1_. This adjustment makes the algorithm execute more cache search and recovery strategies during the early iterations, thereby significantly enhancing its exploration capabilities during this period. As the iterations progress to the later stages, SPNOA favors the execution of the foraging and storage strategy, and according to Equation (29), this strategy has greater local exploitation ability after adjusting the convergence factor *Pa*_1_, which meets the algorithm’s needs in the later phases.

### 4.3. Diversity Attack Strategy

Fu et al. [51] proposed the red-billed blue magpie optimizer (RBMO), inspired by the species’ cooperative and efficient attacking strategies (hereinafter referred to as the magpie). In RBMO, magpies demonstrate exceptional skill levels and collaboration in the pursuit of prey. When magpies act in small groups, their main targets are small prey or plants, and Equation (30) provides the mathematical model for this case. When magpies act in flocks, they can hunt large prey together; Equation (31) provides the mathematical model for this case.

(30) $$X_{i}^{\mathrm{iter}(\mathrm{new})}=X_{\mathrm{food}}^{\mathrm{iter}}+\mathrm{CF}\;\times \;(\frac{1}{q_{1}}\;\times \;\sum_{m=1}^{q_{1}}X_{m}^{\mathrm{iter}}-\;X_{i}^{\mathrm{iter}})\;\times \;{\mathrm{RD}}_{1},$$

(31) $$X_{i}^{\mathrm{iter}(\mathrm{new})}=X_{\mathrm{food}}^{\mathrm{iter}}+\mathrm{CF}\;\times \;(\frac{1}{q_{2}}\;\times \;\sum_{m=1}^{q_{2}}X_{m}^{\mathrm{iter}}-\;X_{i}^{\mathrm{iter}})\;\times \;{\mathrm{RD}}_{2},$$

where $X_{\mathrm{food}}^{\mathrm{iter}}$ denotes the global optimum. *q*_1_ denotes the number of magpies in small groups, which is randomly selected from 2 to 5 from the ebntire population. *q*_2_ denotes the number of magpies in flocks, which is randomly selected from 10 to *N* from the entire population. *RD*_1_ and *RD*_2_ represent the random variable following the standard normal distribution. Equation (32) provides the formula for the generation of CF.

(32) $$\mathrm{CF}={\left(1-(\frac{t}{T_{\max}})\right)}^{\frac{2t}{T_{\max}}}.$$

In NOA’s foraging and storage strategy, the storage phase embodies NOA’s exploitation ability, and its exploitation operator has three different manifestations, demonstrating the diversity of exploitation operators. Although the diversity of exploitation operators enhances NOA’s capacity to escape local optima, it also disrupts the algorithm’s exploitation ability, which means that the original strategy fails to successfully balance exploitation ability and operator diversity. Therefore, to improve the exploitation ability of the foraging and storage strategy, this paper proposes a diversity attack strategy inspired by the attacking behavior of magpies. With the aim of extending the applicability of this strategy to all individuals, Equation (31) is chosen to be improved, and *q*_2_ = *N* is set at the same time. Although the original attack strategy has excellent exploitation ability, the operator is single. Consequently, there is a need to enhance the diversity of the exploitation operators. The improved operator is shown in Equation (33).

(33) Xi,jiter(new)=Xtp2i,tp2jiter, if κ3<0.4,Xi,jiter, otherwise,

(34) Xtp2i,tp2jiter=Xbest,jiter+ CF ×(Xm,jiter−Xi,jiter)×RD2, if κ4<0.9,(1+R2)×Xbest,jiter+RD3×(Xbest,jiter−Xr,jiter), otherwise,

where *R*_2_ is a random variable that adheres to uniform distribution over [−0.1,0.1], *κ*_3_, *κ*_4_ and *RD*_3_ are random numbers within [0,1], and $X_{r,j}^{\mathrm{iter}}$ represents the *j*th dimension of a randomly chosen nutcracker.

### 4.4. Probability-Driven Hybrid Mutation Strategy

Later in the iteration, the algorithm mainly performs exploitation operations, updating around the optimal solution. If the optimization problem tackled by NOA possesses multiple local optimal solutions, the likelihood of premature convergence to the local optimal increases during the solving process. To enhance the improved NOA’s convergence precision and escape local optima, this paper proposes the probability-driven hybrid mutation strategy.

#### 4.4.1. Quasi-Reflection-Based Learning

Opposition-Based Learning (OBL) was proposed by Tizhoosh [52]. Its main principle involves generating a solution that is the antipodal counterpart to the feasible solution, comparing the new solution’s fitness with the original’s fitness, and retaining the superior solution as a new candidate. OBL believes that concurrently analyzing both the original and its opposite solution can help locate the global optimum. OBL has already been used in several algorithms. The generation of the OBL opposite solution is shown in Equation (35).

(35) $$Z_{i,j}^{\mathrm{iter}}=L_{j}+U_{j}-X_{i,j}^{\mathrm{iter}},$$

where $Z_{i,j}^{\mathrm{iter}}$ is the opposite solution corresponding to $X_{i,j}^{\mathrm{iter}}$.

Based on OBL, quasi-opposition-based learning (QOBL) [53] was proposed. Quasi-opposite numbers are used to generate opposite solutions. The definition of the quasi-opposite number is shown in Equation (36).

(36) $${\bar{Z}}_{i,j}^{\mathrm{iter}}=\mathrm{rand}\left({\mathrm{CE}}_{j},Z_{i,j}^{\mathrm{iter}}\right),$$

(37) $${\mathrm{CE}}_{j}=\frac{L_{j}+U_{j}}{2},$$

where *CE_j_* represents the interval center of the *j*th dimension. ${\bar{Z}}_{i,j}^{\mathrm{iter}}$ is a random value between *CE_j_* and $Z_{i,j}^{\mathrm{iter}}$.

Based on OBL and QOBL, Fan et al. [54] proposed a novel quasi-reflection-based learning mechanism known as QRBL. Quasi-reflection number ${\bar{\mathrm{Zs}}}_{i,j}^{\mathrm{iter}}$ is generated by the quasi-opposite number ${\bar{Z}}_{i,j}^{\mathrm{iter}}$. Its definition is shown in Equation (38).

(38) $${\bar{\mathrm{Zs}}}_{i,j}^{\mathrm{iter}}=\mathrm{rand}\left({\mathrm{CE}}_{j},X_{i,j}^{\mathrm{iter}}\right),$$

where ${\bar{\mathrm{Zs}}}_{i,j}^{\mathrm{iter}}$ represents a random between *CE_j_* and $X_{i,j}^{\mathrm{iter}}$.

#### 4.4.2. Adaptive Guided Mutation Strategy

Adaptive guided mutation strategy (AGMS) was proposed by Mohamed et al. [55] in 2019. This strategy proposes a new mutation approach by dividing a population of size *N* into three groups based on the ranking of adaptation values: the first 100*p*% nutcrackers with high adaptation value rankings, the last 100*p*% nutcrackers with low adaptation rankings, and the *N* − 2 (100*p*%) nutcrackers with intermediate adaptation rankings. During the implementation of the mutation strategy, a nutcracker is arbitrarily chosen from each of these three populations, and the specific mutation approach is depicted in Equation (39).

(39) $$\psi_{i}^{\mathrm{iter}}=X_{\mathrm{md}}^{\mathrm{iter}}+F\times \left(X_{p\_\mathrm{best}}^{\mathrm{iter}}-X_{p\_\mathrm{worst}}^{\mathrm{iter}}\right),$$

where $X_{p\_\mathrm{best}}^{\mathrm{iter}}$ represents a nutcracker that is randomly selected from the top 100*p*% of nutcrackers. $X_{p\_\mathrm{worst}}^{\mathrm{iter}}$ denotes a nutcracker that is randomly picked from the bottom 100*p*% of nutcrackers. $X_{\mathrm{md}}^{\mathrm{iter}}$ refers to a nutcracker that is randomly chosen from the remaining *N* − 2 (100*p*%) nutcrackers. In this paper, we set *p* = 0.2. *F* represents a random value within [0.1,1]. Using the fitness value as a selection condition in the mutation strategy firstly ensures that individuals are not invariably drawn towards the best solution identified thus far by the whole population, thereby reducing the risk of falling into local optimality and undergoing premature convergence. Second, by steering clear of exploring in the direction of the least fit individual, the search is directed towards more promising areas, thereby enhancing the algorithm’s capacity for exploration.

In addition to the proportionality factor, the crossover rate *CR* can also influence the mutation effect. In AGMS, *CR* is generated through an adaptive scheme. The crossover rate *CR* of each nutcracker is determined based on one of the two uniform distributions illustrated in Equation (40).

(40) $$\left\{\begin{array}{l} {\mathrm{CR}}_{1}\in [0.05,0.15],\\ {\mathrm{CR}}_{2}\in [0.9,1]. \end{array}\right.$$

The generating sets of these two *CR* are adaptively selected based on the historical success experience of generating promising solutions, as shown in Equation (41).

(41) CRiiter=CR1, if κ5≤p1,CR2, if p1<κ5≤p1+p2,

where *p_j_*, *j* = 1,2 represents the likelihood of choosing the *j*th set. *κ*_5_ is a random number within [0,1]. The sum of the probabilities *p_j_* equals 1. The first time the strategy is implemented, there is not enough experience to choose which set to use, and therefore *p_j_* is initialized to 1/2.

Based on the probability *p_j_*, the roulette approach is utilized to ascertain the appropriate generation set of *CR* for all nutcrackers. In each iteration, the probability *p_j_* is updated according to Equation (42).

(42) *p_j_* = ((*iter* − 1) × *p_j_* + *ps_j_*)/*iter*,

(43) $${\mathrm{ps}}_{j}=\frac{s_{j}}{\sum_{j=1}^{m}s_{j}},$$

(44) $$s_{j}=\frac{{\mathrm{ns}}_{j}}{{\mathrm{ns}}_{j}+{\mathrm{nf}}_{j}}+\varepsilon ,$$

where *iter* is a counter that logs the current number of iterations. *ns_j_* indicates the number of times the update failed after generating *CR* using the *j*th set. *nf_j_* indicates the times of the successful position updates following the selection of the *j*th set. *s_j_* is the probability that the position is successfully updated after generating *CR* using the *j*th set for the crossover rate. *ps_j_* indicates the odds of picking the *j*th set to produce *CR*. *ε* is an exceedingly small constant value used to avoid the extreme case in which one of the sets is selected 0 times in an iteration. In this paper, this variable is set to 0.0001. When the success rate *s_j_* is 0 for both sets, *p_j_* is reinitialized to 0.5 to overcome possible stagnation.

To enhance the capacity of the improved NOA to evade local optima, we integrated AGMS with QRBL into one strategy, and the two mutation strategies were executed alternately, as shown in Equation (45).

(45) Xiiter(new)=Equation (38), if κ6<PR1,Xtp3i,tp3jiter, otherwise,

(46) Xtp3i,tp3jiter=Equation (39), if κ7<PR2,Xi,jiter, otherwise,

(47) $${\mathrm{PR}}_{1}=0.3-0.1\times \frac{t}{T_{\max}},$$

(48) $${\mathrm{PR}}_{2}=0.5+0.4\;\times \;\sin((\frac{t}{T_{\max}}-\;0.5)\;\times \;\pi ),$$

where *κ*_6_ and *κ*_7_ are random numbers within [0,1].

### 4.5. Computational Complexity

The computational complexity of SPNOA is analyzed in terms of the population size *N*, the problem dimension *D*, and the maximum number of function calculations *T_max_*. The analysis is split into two stages: the initialization stage and the iterative search stage. During the initialization stage, the specular reflection learning strategy introduces an additional *O*(*N* × *D*) operations during the initialization process. Therefore, the fitness values of 2*N* individuals need to be evaluated, resulting in a cost of *O*(2 × *N* × *D*). During the iterative search stage, SPNOA terminates upon exhausting the prescribed function-evaluation budget *T_max_*, instead of using a fixed iteration limit. The probability-driven hybrid mutation strategy may introduce up to 2*N* extra function calculations per iteration. These evaluations consume the same *T_max_* budget, which reduces the total completed iterations but does not exceed the prescribed evaluation budget. Accordingly, the objective-function evaluation cost in the iterative search phase is *O*(*D* × (*T_max_* − 2 × *N*)). Thus, the computational complexity of SPNOA presented is

(49) $$\begin{array}{ll} O(\mathrm{SPNOA}) & =O(\mathrm{Initialization}\;\mathrm{phase})+O(\mathrm{Iterative}\;\mathrm{phase})\\ & =O(2\times N\times D)+O(D\times (T_{\max}-2\times N))\\ & =O(T_{\max}\times D) \end{array}$$

**Figure 3:**
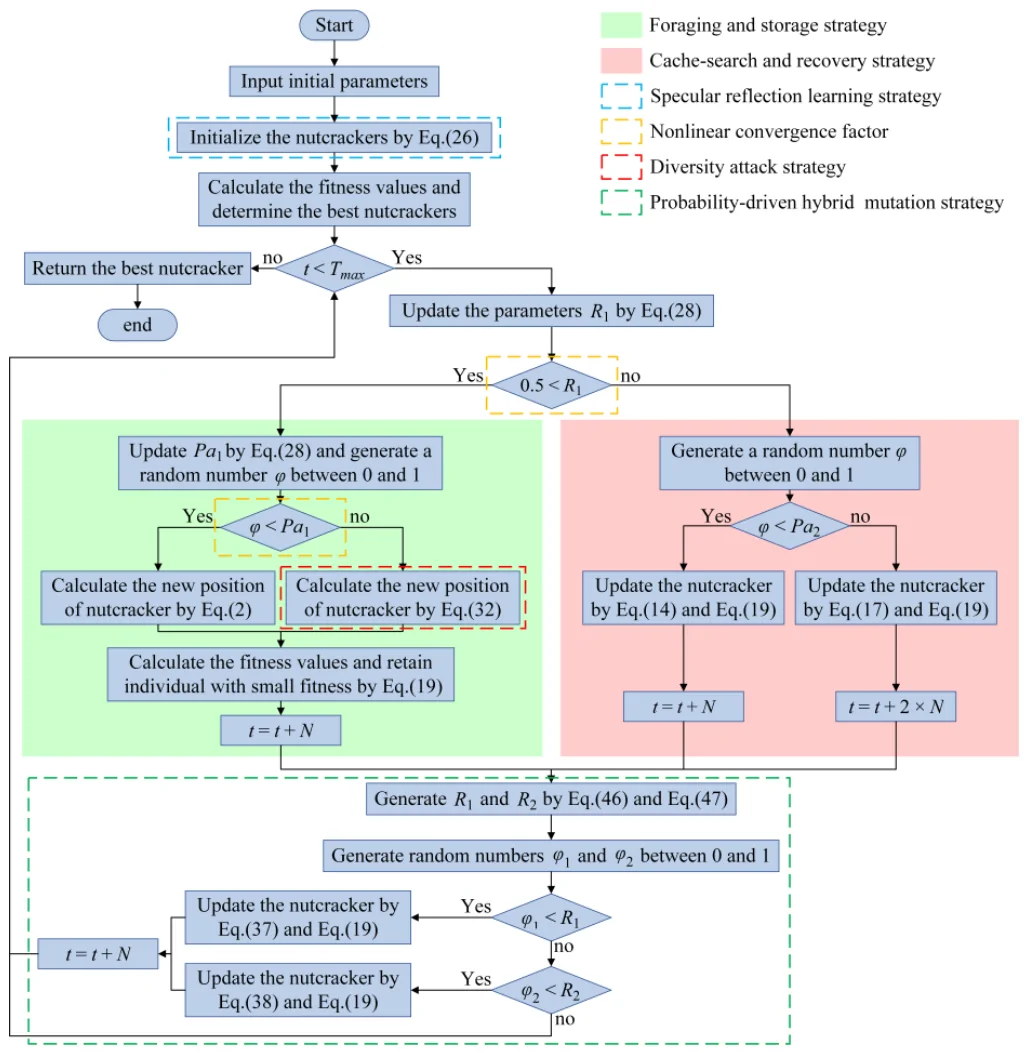
The flowchart of SPNOA.

To demonstrate the modified algorithm more intuitively, the pseudo-code of SPNOA is given in Algorithm 2, while its flowchart is shown in Figure 3.

**Algorithm 2:** Pseudo-code for SPNOA**Input**: *N*(Population size), *dim*(Population dimension), *U*(Upper limit), *L*(Lower limit), *t*(Current number of function calculations), *T_max_*(Maximum number of function calculations), *iter*(Current number of iteration loops).**Output**: The best Nutcracker *X_best_* and its corresponding fitness.Based on SRL, using Equation (26) to initialize nutcracker/solution.Calculate each solution of the population and select the optimal one from it. Generate the nonlinear *R*_1_ according to Equation (28).*t* = 2 × *N*; *iter* = 1;**while** *t* < *T_max_*
**If** 0.5 < *R*_1_// Foraging and storage strategy //

Generate random number *φ* in [0,1].

Generate the nonlinear $P_{a_{1}}$ according to Equation (28).

**for** *i* = 1: *N*


**If** $\varphi <P_{a_{1}}$/ Exploration phase 1/



Update $X_{i}^{\mathrm{iter}}$ using Equation (2) and Equation (19).



**else**
/Exploitation phase 1/



Based on diversity attack strategy, using Equation (33) and Equation (19) to update $X_{i}^{\mathrm{iter}}.$



**end if**



Current number of function calculations *t* = *t* + 1.


**end for**


**else**
//Cache-search and recovery strategy//

Generate RP matrix using Equation (8) and Equation (9).

Generate random number *ϕ* in [0,1].

**for** *i* = 1: *N*


**If** $\phi >P_{a_{2}}$/ Exploration phase 2/



Update $X_{i}^{\mathrm{iter}}$ using Equation (14) and Equation (19).



Current number of function calculations *t* = *t* + 2.



**else**
/ Exploitation phase 2/



Update $X_{i}^{\mathrm{iter}}$ using Equation (17) and Equation (19).



Current number of function calculations *t* = *t* + 1.



**end if**



**end for**


**end if**

Generate *RP*_1_ according to Equation (47).// Probability-driven hybrid mutation strategy //
Generate *RP*_2_ according to Equation (48).

Generate random numbers *κ*_6_ and *κ*_7_ that obey a uniform distribution in [0,1].
**if** *κ*_6_ < *RP*_1_/QRBL/

**for** *i* = 1: *N*


Based on QRBL, using Equation (38) and Equation (19) to update $X_{i}^{\mathrm{iter}}.$


Current number of function calculations *t* = *t* + 1.


**end for**

**elseif** *κ*_7_ < *RP*_2_/AGMS/

**for** *i* = 1: *N*


Based on AGMS, using Equation (39) and Equation (19) to update $X_{i}^{\mathrm{iter}}.$


Current number of function calculations *t* = *t* + 1.


**end for**


**end if**

Current number of iteration loops *iter* = *iter* + 1.
**end while**


### 4.6. Space Complexity

In terms of space complexity, NOA stores the population, fitness values, and temporary candidates, requiring *O*(*N* × *D*) memory for an *O*(*N* × *D*) population matrix. SPNOA additionally generates reflection, attack, and mutation candidates, but these require only a constant number of reusable *N* × *D* matrices. Thus, its space complexity is *O*(*c* × *N* × *D*), where *c* is a constant, indicating only a constant-factor increase in memory usage.

## 5. Numerical Experiment Outcomes and Analysis

We evaluate SPNOA’s performance by conducting a comprehensive set of experiments. First, a sensitivity analysis is performed on the three parameters to determine their optimal values. Second, the performance of SPNOA is tested by comparing SPNOA with 16 representative high-performance algorithms on 10, 20, and 50 dimensions of the CEC2020 test suite. Then, statistical tests are used to assess the differences between SPNOA and the comparison algorithms, thereby further validating the performance of SPNOA and the need to improve the original NOA. Finally, an analysis of the algorithms’ convergence behavior is conducted. All numerical experiments in this article are operated in “Windows 11(64-bit)”, “AMD Ryzen 7 7745HX with Radeon Graphics @ 3.6GHz 16 GB”, “MATLAB 2020b”.

### 5.1. Benchmark Functions and Comparison Algorithms

We employ the CEC2020 test suite to assess the performance of SPNOA. The CEC2020 test suite comprises ten representative optimized test functions, which are the single-peak function F1, the basic functions F2–F4, the hybrid functions F5–F7, and the composition functions F8–F10. Table S2 in the Supplementary Material summarizes the full details of the CEC2020 test suite, including the function names, search bounds, and corresponding optimal objective values.

To obtain a more complete picture of SPNOA’s comprehensive performance, it is essential to benchmark it against other high-performance algorithms, and the comparison algorithms chosen include NOA. The newly proposed algorithms are APO [56], AROA [57], BWO [58], HEOA [59], RBMO [51], and KOA [60]. The improved algorithms are HFPSO [61], EAPSO [62], ACEPSO [63], RNEGDE [64], MCDE [65], and HMRFO [66]. The CEC competition-winning algorithms are IMODE [67], AGSK [68], and LSHADE_cnEpSin [69]. The compared algorithms are configured according to the parameter settings recommended in their original publications, without additional problem-specific returning. For SPNOA, a single parameter configuration is selected through sensitivity analysis on the 30-dimensional CEC2020 test suite. This configuration is then kept unchanged for the 10-, 20-, and 50-dimensional CEC2020 test suites and the 27 engineering design optimization problems. Table 3 summarizes the core strategies and inherent limitations of several CEC competition-winning algorithms and SPNOA.

All the experiments in this section are conducted on the CEC2020 test suite. The population size *N* is uniformly set to 30, and the maximum number of function calculations *T_max_* is set to *D* × 10000, with 30 independent runs for each algorithm. Here, *D* represents the dimension of the problem.

### 5.2. Performance Indicators

To effectively analyze the experimental outcomes presented in this paper and to more intuitively illustrate the performance disparities among various algorithms, the following six performance indicators need to be used:

1. The best value (Best) represents the optimal solution that an algorithm can achieve for a given optimization problem, and its definition is shown in Equation (50).
2. The worst value (Worst) demonstrates the limitations of an algorithm by showing the most unfavorable outcome it produces, which is defined as shown in Equation (51).
3. The mean value (Mean) is a commonly used statistic to measure the level of data, because the algorithm has strong randomness, so the mean value offers a more accurate reflection of the algorithm’s effect, which is defined as shown in Equation (52).
4. Standard deviation (Std) is a metric that quantifies the dispersion of data within a dataset. A smaller Std indicates that the data points are more tightly clustered around the mean, suggesting greater stability in the algorithm. Its definition is shown in Equation (53).
5. Rank (Rank) is the ranking obtained by all algorithms in the order best to worst according to the average value when dealing with an optimization problem. This metric indicates the relative effectiveness of each algorithm on that problem. Mean Rank is an indicator that reflects the algorithm’s overall performance. A lower Mean Rank indicates better overall performance.
6. The average calculation time (Time), measured in seconds, is the mean wall-clock time required to complete one independent run on a given test function at a specified dimensionality. Each algorithm is executed 30 times under identical hardware and software settings, with the same population size and maximum number of function calculations. The reported Time is the average across these runs; lower values indicate lower computational cost and higher efficiency.

(50) Best = min(*fit*_1_,*fit*_2_,…,*fit_n_*)

(51) Worst = max(*fit*_1_,*fit*_2_,…,*fit_n_*)

(52) $$\mathrm{Mean}=\frac{{\mathrm{fit}}_{1}+{\mathrm{fit}}_{2}+\ldots +{\mathrm{fit}}_{\mathrm{runs}}}{\mathrm{runs}}$$

(53) $$\mathrm{Std}=\sqrt{\frac{1}{\mathrm{runs}-1}\sum_{k=1}^{\mathrm{runs}}{\left({\mathrm{fit}}_{1}-\mathrm{Mean}\right)}^{2}}$$

where *fit_k_* represents the optimal value achieved during the *k*th execution and *runs* denotes the maximum number of executions.

### 5.3. Ablation Experiment

To evaluate the contributions of the four strategies, this section compares the original NOA, variants integrated with each individual strategy, and variants derived by removing one strategy from the full SPNOA. Each variant is named using the initials of its incorporated strategies. For instance, NOA-S denotes NOA embedded with the specular reflection learning strategy, whereas NOA-SND represents SPNOA with the probability-driven hybrid mutation strategy removed. The ablation experiments are carried out on the 10-dimensional CEC2020 test suite, and the experimental results are listed in Table 4.

Results from single-strategy variants reveal that each strategy contributes differently to the algorithm. Integrating all four strategies does not guarantee that the improved algorithm achieves optimal performance on every function. For example, NOA-D achieves lower objective values than SPNOA on F3 and F6, demonstrating that the standalone diversity attack strategy yields better performance for these two functions. Comparative removal tests further show that NOA-SND, which excludes the probability-driven hybrid mutation strategy, underperforms SPNOA on F1 and F4, verifying the positive effect of this mutation strategy on algorithm performance. Overall, the ablation results validate the effectiveness of these strategies for boosting the proposed SPNOA performance.

### 5.4. Sensitivity Analysis

Following the parameter selection approach of the original NOA study, this section focuses on *δ*, *P_rp_*, and *Pa*_2_, which govern dimension-wise updating, the exploration of other regions, and the selection between the cache search and recovery phases, respectively. This analysis therefore focuses on the effects of the original NOA control parameters in SPNOA. For the parameters introduced by the additional strategies, the settings were mainly adopted from the corresponding original studies. For example, the crossover rate ranges *CR*_1_ in [0.05,0.15] and *CR*_2_ in [0.9,1] were adopted from AGMS [55] and left unchanged. The selection probabilities for these ranges were initialized at 0.5 and updated according to their success histories, following the rule described in Section 4.4.2. The probability schedules *PR*_1_ and *PR*_2_, as defined in Equations (47) and (48), are not independently specified parameters within the strategy; therefore, they were not included in the sensitivity analysis.

When analyzing one parameter, the remaining parameters were kept at their predefined settings. The sensitivity analyses of all three parameters were chosen to be conducted on the CEC2020 test suite, with the test dimension *D* = 30. The final experimental results are reported as the mean of the outcomes from these 50 independent replications, and the optimal parameter value is the one that corresponds to the outcome with the lowest Mean rank.

To achieve a more precise determination of the optimal values for different parameters, 12 different values were set for *δ*, and 11 different values were set for both *P_rp_* and *Pa*_2_. In the experiment, it was found that no matter how the values of the three key parameters change, the algorithm can find the optimal value of the function F4; that is, the function is not comparatively meaningful, so F4 is deleted in the results. The final experimental data are shown in Table 5, Table 6 and Table 7. Table 5 and Table 6 indicate that the optimal values of *δ* and *P_rp_* are both 0.1. To determine the optimal value of parameter *Pa*_2_, Table 7 shows that the Mean Ranks are the same when *Pa*_2_ is 0.3 and 0.5. However, three functions are ranked first when *Pa*_2_ is 0.3, whereas only one function is ranked first when *Pa*_2_ is 0.1. Therefore, the final value selected for *Pa*_2_ is 0.3.

The different performances under different parameter settings result from their distinct effects on the search process. A larger *δ* increases dimension-wise perturbations, *P_rp_* enhances the exploration of alternative regions, and *Pa*_2_ increases the probability of selecting the recovery phase. The selected values *δ* = 0.1, *P_rp_* = 0.1, and *Pa*_2_ = 0.3 therefore provide a suitable balance between exploration and exploitation. These values were also used unchanged in the 10-, 20-, and 50-dimensional experiments and the real-world engineering problems, where SPNOA remained competitive, providing indirect evidence of their robustness.

### 5.5. Comparative Analysis of Convergence Factor Forms

To investigate the effect of the exponent in the nonlinear convergence factor, four forms were compared: the original linear form and the power-based nonlinear forms with exponents of 2, 4, and 6. The first convergence factor and all other components of SPNOA were left unchanged during the comparison. Therefore, the experiment isolates the influence of the second convergence factor.

The complete results are provided in Table S1 of the Supplementary Materials. The fourth-power form achieves the lowest mean rank of 1.9, compared with 2.7 for the linear form, 2.6 for the second-power form, and 2.2 for the sixth-power form. It also obtains the best mean results on most test functions. These results support the use of the fourth-power nonlinear factor in SPNOA.

### 5.6. CEC2020 Test Suite Result Analysis

To comprehensively investigate the overall performance and optimization behavior of SPNOA, we conduct performance experiments of SPNOA with 16 competing algorithms on the CEC2020 test suite at dimensions of 10, 20, and 50. Nonparametric statistical tests and data visualization are further performed on the 10-dimensional results to verify the effectiveness and necessity of the proposed SPNOA.

#### 5.6.1. Experimental Results and Analysis

The experimental outcomes are summarized in Table 8, Table 9 and Table 10. For the single-peak function F1, SPNOA demonstrates convergence to the optimal solution in both 10 and 20 dimensions when considering Mean and Std. However, the algorithm exhibits a slight reduction in stability as the problem dimensionality increases. This decline in stability results in a drop in SPNOA’s ranking from first to fifth, although it converges to the optimum. In contrast, NOA does not fully converge to the optimum in either dimension, and NOA is less stable than SPNOA in both dimensions. In the 50-dimensional, both SPNOA and NOA fail to converge to the optimum and the results show large fluctuations, but from the values of Std and Worst, the stability of SPNOA is still stronger than that of NOA. Therefore, it can be concluded that the four strategies added to the algorithm do enhance the stability and performance of SPNOA.

For the basis function F2, SPNOA’s Mean ranks first in all three selected dimensions. The Std of SPNOA in the 10 dimensions also outperforms all compared algorithms. Although the Std of SPNOA in the 20 and 50 dimensions increases with increasing dimensionality, it is still smaller than the Std of NOA in the corresponding dimensions. SPNOA ranks first in the 10- and 20-dimensional cases and second in the 50-dimensional case, indicating that it generally maintains superior convergence accuracy and stability across the three dimensions. For the basis function F3, SPNOA ranked in the top three in all three selected dimensions. In contrast, NOA’s Rank fluctuates more, and the optimal result occurs in the 50-dimensional case, but at this point, the Rank is only fourth. The algorithms ranked first across these three dimensions (ranging from 10 to 50 dimensions) are APO, IMODE, and ACEPSO, respectively. This further demonstrates that SPNOA exhibits better stability than the comparative algorithms. SPNOA’s Best value is lower than NOA’s in all three dimensions. As for the basis function F4, NOA was able to fully converge to the optimum, and SPNOA retained the strengths of the original NOA and was able to converge to the optimal with stability. LSHADE_cnEpSin also achieves competitive results on these basic functions. However, SPNOA generally obtains comparable or better Mean values across the three dimensions, further demonstrating its competitiveness against this state-of-the-art algorithm.

For the hybrid functions F5-F7, SPNOA ranks first in almost all cases in 10 and 20 dimensions and ranks third for function F6 in 20 dimensions, whereas NOA ranks fifth. In 50 dimensions, although SPNOA ranks first only for function F6, SPNOA’s Mean and Std are both better than NOA’s in this dimension. Therefore, it can be considered that SPNOA’s performance is nevertheless improved compared to that of NOA, with the advantage being more prominent in problems of lower dimensionality.

For the composition functions F8 and F9, SPNOA cannot take the absolute lead in any of these three specified dimensions, but it can rank among the top four and maintain relatively small fluctuations, which shows that SPNOA has few disadvantages across different test functions. Relative to the original algorithm, SPNOA has a higher Mean than NOA only for function F8 in 10 dimensions, and SPNOA performs better than NOA in the rest of the cases. For the composition function F10, SPNOA ranks first in both 10 and 50 dimensions. SPNOA ranks third in 20 dimensions, while the first-ranked algorithm is the CEC competition-winning algorithm AGSK. The Best values indicate that SPNOA’s convergence accuracy outperforms NOA’s in both 20 and 50 dimensions. Compared with LSHADE_cnEpSin, SPNOA achieves better Mean values on most of the F8–F10 functions. LSHADE_cnEpSin performs slightly better on F10 at 20 dimensions, indicating that SPNOA has strong overall competitiveness but does not dominate every individual problem.

In addition to solution quality, the computational time of the algorithms was evaluated using the average time reported in Table 8, Table 9 and Table 10. Under the same hardware, software environment, population size, termination criterion, and function-evaluation budget, SPNOA requires only slightly more execution time than NOA. Although SPNOA introduces additional operations, its time overhead remains limited. Across the ten CEC2020 test functions, the maximum execution times recorded for LSHADE_cnEpSin reached approximately 1.46 s, 2.98 s, and 19.12 s at 10, 20, and 50 dimensions, respectively. These results indicate that the additional computational cost of SPNOA is modest, while its execution time remains lower than that of the added SOTA comparator. Overall, the improvement in solution quality is achieved without a substantial increase in computational time.

#### 5.6.2. Statistical Testing

Nonparametric statistical tests were conducted on the fitness data of the 10-dimensional CEC2020 test suite obtained from 30 independent runs for each algorithm. Specifically, the Friedman test [70] was used to perform an overall performance assessment across all algorithms, while the Wilcoxon rank-sum test [71] was used to carry out pairwise comparisons. The Wilcoxon rank-sum test was chosen over the Wilcoxon signed-rank test as the 30 repeated runs of each algorithm per function were independent and unpaired.

The Friedman test is often employed to assess the overall performance of multiple optimization algorithms. The Friedman test will also yield different sequence values according to the order of the algorithm performance from good to poor. The better the algorithm’s performance, the lower its assigned rank value, and if two algorithms achieve identical performance, their rank values are divided evenly. The test results are detailed in Table 11, where the best average is indicated in bold black font. It is evident that the SPNOA presented in this paper has the smallest Friedman mean ranking among these 17 algorithms, followed by IMODE. This result indicates that SPNOA achieves the best overall Mean Rank among the compared algorithms.

The Wilcoxon rank-sum test is applicable to determine if there is a statistically significant performance difference between SPNOA and comparative algorithms. For each test function, the Wilcoxon test compared a sample of 30 fitness values from SPNOA with a separate sample of 30 fitness values from each competing algorithm, usually *p* = 0.05. If *p* < 0.05, it suggests that there is a significant performance difference between SPNOA and the compared algorithm. If *p* ≥ 0.05, it indicates that there is no significant performance difference. Table 12 shows the experiment results. “+” means SPNOA outperforms the comparison algorithm significantly; “-” means the comparison algorithm outperforms SPNOA significantly; and “=” means that there is no significant difference between SPNOA and the comparison algorithm. “NaN” indicates that both SPNOA and the comparison algorithm converged stably to the optimum across the 30 repeated experiments. Therefore, this symbol signifies that there is no statistically significant difference between SPNOA and compared algorithms. Table 12 shows that SPNOA outperforms the comparison algorithms in most of the tested functions. The cases in which there is no significant difference between the algorithms with fewer occurrences are mostly the ones indicated by the symbol “NaN”. These results support the effectiveness and superiority of SPNOA.

#### 5.6.3. Data Visualization and Analysis

To gain insight into the convergence process of SPNOA, the experimental results need to be visualized. For consistency with 5.6.2, 10-dimensional experimental data are still chosen for graphing in this section.

**Figure 4:**
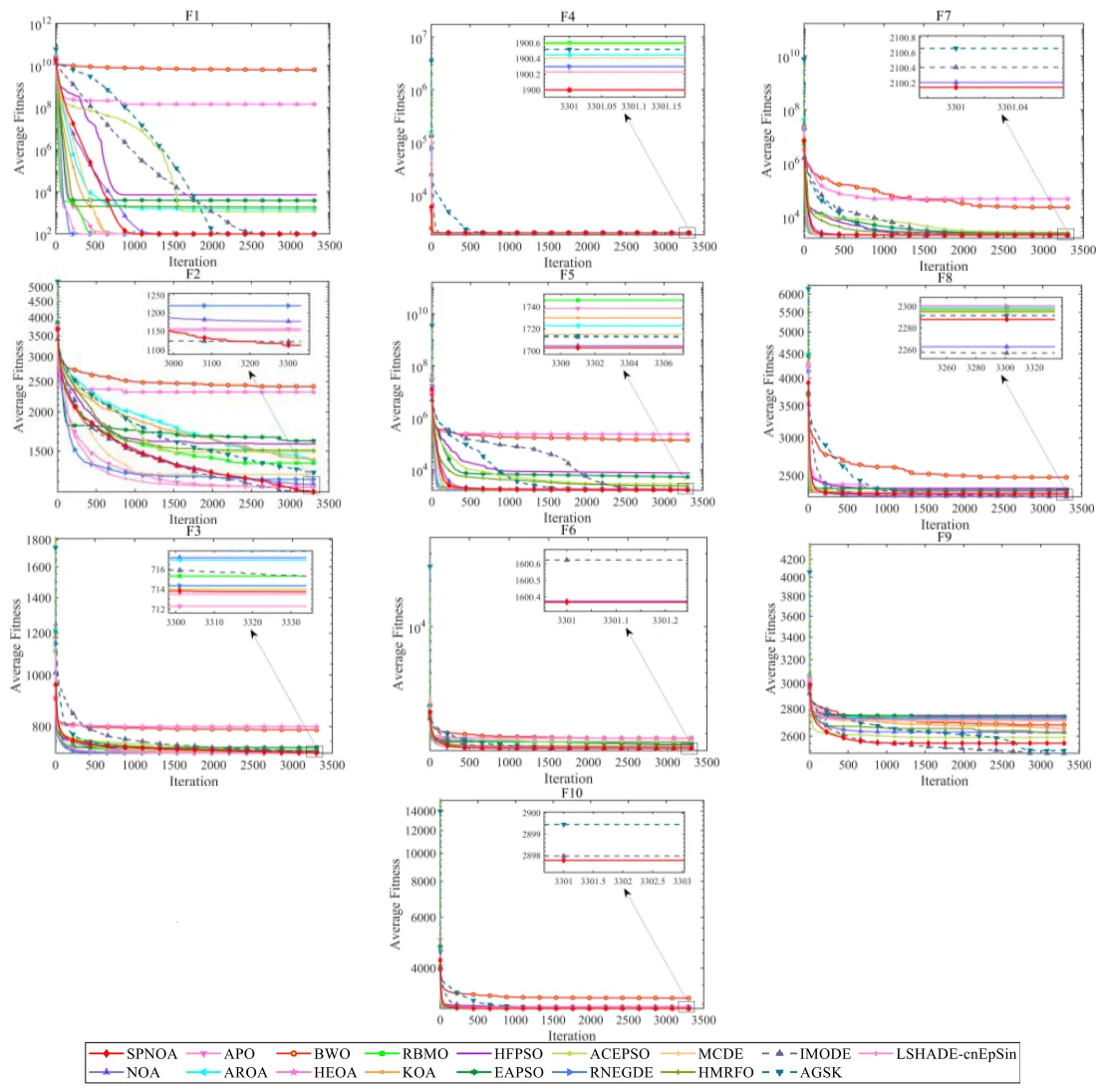
The Convergence curves.

The convergence curves provide a visual means to understand the convergence process of various algorithms. Figure 4 shows the convergence curve, with the red curve representing SPNOA proposed in this paper. From the F2 function, it is evident that the convergence process of SPNOA is more stable. In the F1 and F4 functions, SPNOA reaches the global optimum more quickly than competing algorithms. From F9 and F10, SPNOA exhibits better convergence speed and accuracy than NOA. This suggests that adding the mutation strategy can assist SPNOA in escaping local optima to some extent, thereby aiding in the search for better solutions.

**Figure 5:**
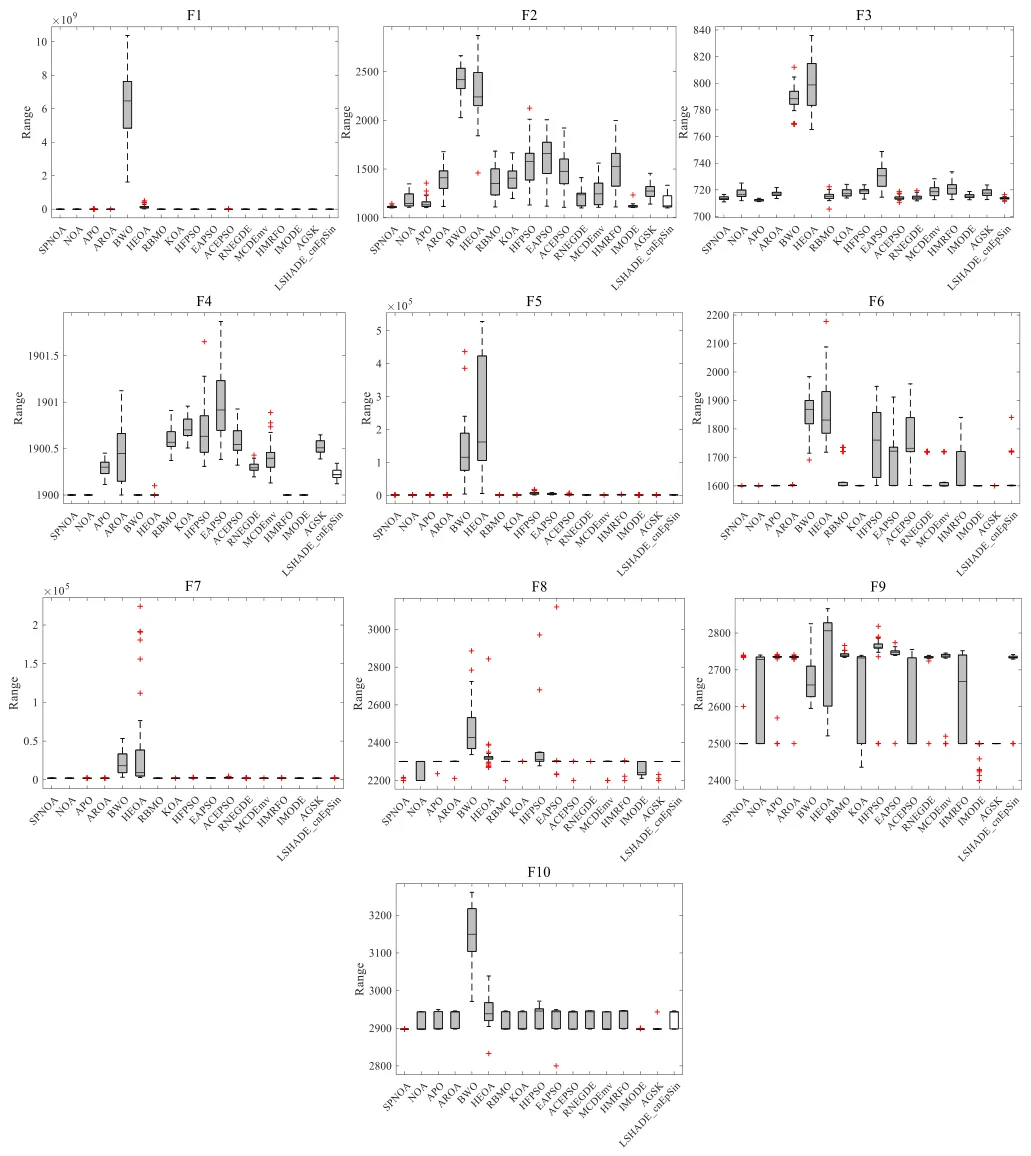
The Boxplots.

Boxplots are capable of showing the dispersion and centralizing trends within a dataset. Therefore, the use of boxplots can help understand the performance and stability of optimization algorithms, as presented in Figure 5. The longer the box, the more scattered the data, and the less stable the algorithm. The red symbol “+” indicates outliers in the data. From the boxplot, it can be seen that, in most cases, SPNOA has the shortest box, the lowest position, and the fewest outliers. This suggests that SPNOA exhibits superior stability and convergence accuracy.

**Figure 6:**
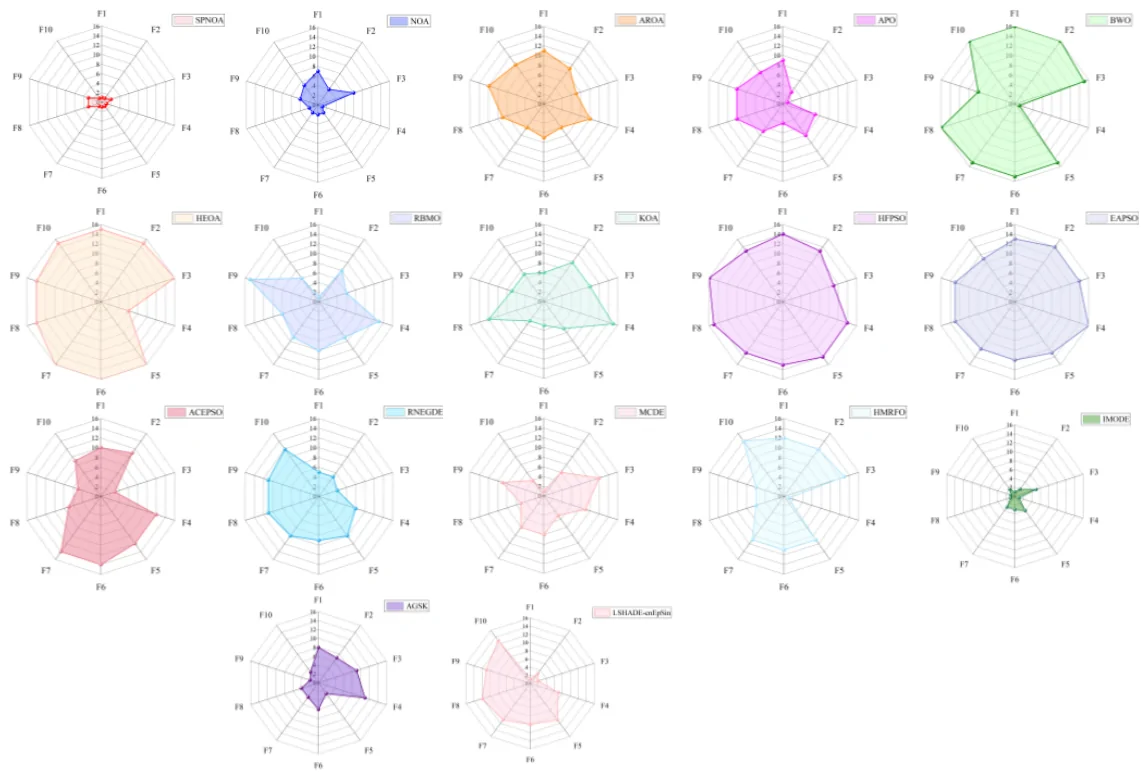
The Radar charts.

The radar chart not only visualizes the ranking of the algorithms across different functions, but also makes it possible to judge the overall performance of the algorithms through the size of the shaded area. Figure 6 shows the radar charts of SPNOA and the comparison algorithm. From the radar plot, it is evident that SPNOA has the smallest shadow area, followed by the contest-winning algorithm IMODE. However, these two algorithms are not ranked first across all functions. Although BWO, RBMO, and HMRFO have larger shadow areas, they achieve first place in specific functions. This phenomenon proves the NFL.

## 6. Engineering Design Optimization

### 6.1. Introduction to Engineering Examples

**Figure 7:**
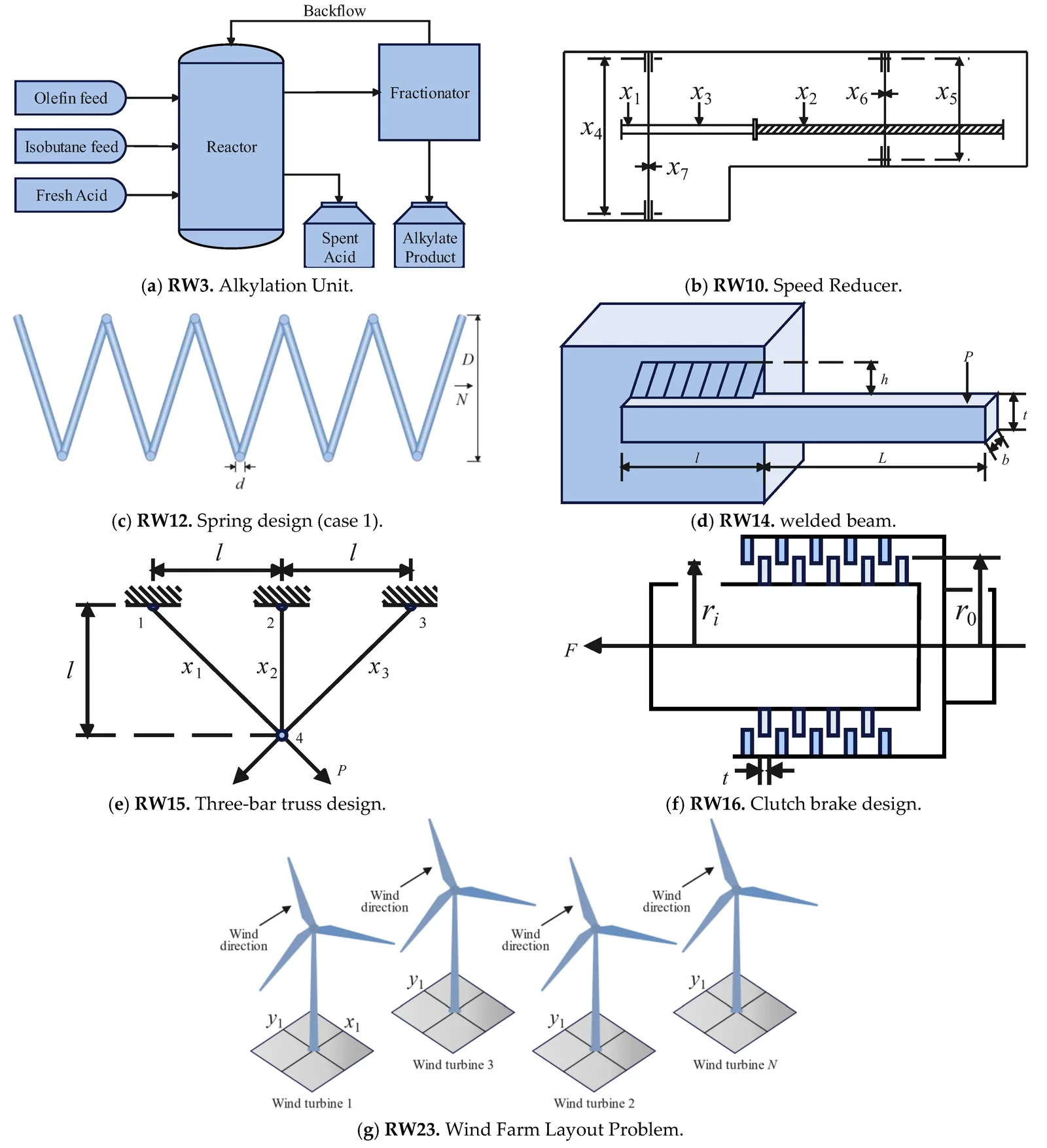
Schematic diagram of some engineering optimization problems.

In this section, we employ 27 constrained optimization problems extracted from the CEC2020 real-world problem test suite. These problems are categorized into five main groups, including industrial chemical processes (RW01-RW03), process synthesis and design (RW04-RW09), mechanical engineering (RW10-RW22), electric power systems (RW23), and power electronics (RW24-RW27). Figure 7 shows the model sketches of some of the optimization problems. The detailed information concerning the 27 real optimization problems is presented in Table S3 of the Supplementary Materials, where *D* refers to the problem dimension, *g*(*z*) denotes the number of inequality constraints, *h*(*z*) signifies the number of equation constraints, and f*(*z*) indicates the theoretical optimal solution. In this section, one representative problem in each category is chosen to be presented.

#### 6.1.1. Optimal Operation of Alkylation Unit (OOAU)

OOAU [72] is a constrained optimization problem for industrial chemical processes. The primary objective is to maximize the production of alkylation products from olefin feedstock following a series of chemical reactions that occur in an acidic environment. Consequently, the amount of alkylation produced is an objective function of this problem. The problem contains seven variables and 14 nonlinear constraints. The mathematical model based on OOAU is:

(54) Maximum *f*(*z*) = 0.035*z*_1_*z*_6_ - 0.063*z*_3_*z*_5_ + 1.715*z*_1_ + 10.0*z*_2_ + 4.0565*z*_3_

Subject to:

$$\begin{array}{c} g_{1}(z)=6.66173269z_{6}^{2}-191.20592z_{6}+172.39878z_{5}-56.596669z_{4}-10000\leq 0,\\ g_{2}(z)=0.0059553571z_{6}^{2}z_{1}-0.1175625z_{6}z_{1}+0.0059553571z_{3}-z_{1}\leq 0\\ g_{3}(z)=0.006198z_{7}z_{4}z_{3}-25.125634z_{2}z_{4}-z_{3}z_{4}+2462.3121z_{2}\leq 0,\\ g_{4}(z)=1.08702z_{6}-0.03762z_{6}^{2}-z_{5}+0.32175z_{4}+56.85075\leq 0,\\ g_{5}(z)=161.18996z_{3}z_{4}-z_{3}z_{4}z_{7}+5000.0z_{2}z_{4}-489510.0z_{2}\leq 0,\\ g_{6}(z)=1.1088z_{1}-0.0066033z_{6}^{2}z_{1}+0.1303533z_{1}z_{6}-z_{3}\leq 0,\\ g_{7}(z)=1020.4082z_{4}z_{2}+1.2244898z_{3}z_{4}-100000z_{2}\leq 0,\\ g_{8}(z)=6.25z_{1}z_{6}-7.625z_{3}+6.25z_{1}-100000\leq 0,\\ g_{9}(z)=0.022556z_{5}-0.007595z_{7}-1\leq 0,\\ g_{10}(z)=0.819692z_{1}-z_{3}+0.819672\leq 0,\\ g_{11}(z)=24500.0z_{2}-250.0z_{2}z_{4}-z_{3}z_{4}\leq 0,\\ g_{12}(z)=0.33z_{7}-z_{5}+44.333333\leq 0,\\ g_{13}(z)=0.00061z_{3}-0.0005z_{1}-1\leq 0,\\ g_{14}(z)=1.22z_{3}-z_{6}z_{1}-z_{1}+1\leq 0. \end{array}$$

With

$$\begin{array}{l} 1000\leq z_{1}\leq 2000,0\leq z_{2}\leq 100,2000\leq z_{3}\leq 4000,0\leq z_{4}\leq 100,\\ 0\leq z_{5}\leq 100,0\leq z_{6}\leq 20,0\leq z_{7}\leq 200. \end{array}$$

#### 6.1.2. Process Synthesis Problem (PSP)

PSP [73] is a constrained optimization problem in process synthesis and design. The problem is the nonconvex optimization issue that includes both continuous and discrete variables, comprising seven variables and nine nonlinear constraints. The mathematical model based on PSP is:

(55) $$\begin{array}{ll} \mathrm{Minimize f}(z) & ={\left(z_{1}-1\right)}^{2}+{\left(z_{2}-1\right)}^{2}+{\left(z_{3}-1\right)}^{2}-\ln(z_{7}+1)\\ & +{\left(z_{4}-1\right)}^{2}+{\left(z_{5}-1\right)}^{2}+{\left(z_{6}-1\right)}^{2} \end{array}$$

Subject to:

$$\begin{array}{c} g_{1}(z)=z_{1}+z_{6}+z_{2}+z_{5}+z_{3}+z_{4}-5\leq 0,\\ g_{2}(z)=z_{6}^{3}+z_{1}^{2}+z_{2}^{2}+z_{3}^{2}-5.5\leq 0,\\ g_{3}(z)=z_{5}^{2}+z_{3}^{2}-4.64\leq 0,\\ g_{4}(z)=z_{5}^{2}+z_{2}^{2}-1.64\leq 0,\\ g_{5}(z)=z_{6}^{2}+z_{3}^{2}-4.25\leq 0,\\ g_{6}(z)=z_{1}+z_{7}-1.2\leq 0,\\ g_{7}(z)=z_{2}+z_{5}-1.8\leq 0,\\ g_{8}(z)=z_{3}+z_{6}-2.5\leq 0,\\ g_{9}(z)=z_{1}+z_{4}-1.2\leq 0. \end{array}$$

With

$$\begin{array}{c} 0\leq z_{3},z_{2},z_{1}\leq 100,\\ 0<z_{7},z_{6},z_{5},z_{4}<1. \end{array}$$

#### 6.1.3. Weight Minimization of a Speed Reducer (WMSR)

WMSR [74] represents a constrained optimization problem within the realm of mechanical engineering, with its main goal being the minimization of the total weight of a speed reducer designed for a small plane. The problem contains seven variables and 11 nonlinear constraints. The mathematical model based on WMSR is:

(56) $$\begin{array}{ll} \mathrm{Minimize f}(z) & =0.7854z_{2}^{2}z_{1}((3.3333z_{3}+14.9334)z_{3}-43.0934)\\ & +\;0.7854(z_{5}z_{7}^{2}+z_{4}z_{6}^{2})-1.508z_{1}(z_{7}^{3}+z_{6}^{2})+7.477(z_{7}^{3}+z_{6}^{3}) \end{array}$$

Subject to:

$$\begin{array}{c} g_{1}(z)=10z_{6}^{-3}\sqrt{16.91\times 10^{6}+{\left(745z_{4}z_{2}^{-1}z_{3}^{-1}\right)}^{2}}-1100\leq 0,\\ g_{2}(z)=10z_{7}^{-3}\sqrt{157.5\times 10^{6}+{\left(745z_{5}z_{2}^{-1}z_{3}^{-1}\right)}^{2}}-850\leq 0,\\ g_{3}(z)=-z_{2}z_{6}^{4}z_{3}z_{4}^{-3}+1.93\leq 0,\\ g_{4}(z)=-z_{2}z_{7}^{4}z_{3}z_{5}^{-3}+1.93\leq 0,\\ g_{5}(z)=-z_{1}z_{2}^{2}z_{3}^{2}+397.5\leq 0,\\ g_{6}(z)=1.5z_{6}-z_{4}+1.9\leq 0,\\ g_{7}(z)=1.1z_{7}-z_{5}+1.9\leq 0,\\ g_{8}(z)=-z_{1}z_{2}^{2}z_{3}+27\leq 0,\\ g_{9}(z)=z_{1}z_{2}^{-1}-12\leq 0,\\ g_{10}(z)=-z_{1}z_{2}^{-1}+5\leq 0,\\ g_{11}(z)=z_{2}z_{3}-40\leq 0. \end{array}$$

With

$$\begin{array}{c} 5\leq z_{7}\leq 5.5,2.9\leq z_{6}\leq 3.9,7.3\leq z_{5},z_{4}\leq 8.3,\\ 17\leq z_{3}\leq 28,0.7\leq z_{2}\leq 0.8,2.6\leq z_{1}\leq 3.6. \end{array}$$

#### 6.1.4. Wind Farm Layout Problem (WFLP)

WFLP [75] represents a constrained optimization problem within the domain of power systems. Wind farm layout is one of the pivotal factors that determine the energy output of the wind farm throughout its operational lifespan. The main goal of WFLP is to enhance overall power output. In this case, the total power generation is the objective function. The problem contains 30 variables and three constraints. The mathematical model based on WFLP is:

(57) Maximum f =∑i=1NE(Pi)

Subject to:

$$\begin{array}{c} \sqrt{{\left(x_{i}-y_{j}\right)}^{2}+{\left(y_{i}-y_{j}\right)}^{2}}\geq 5F_{r},j=1,2,\ldots ,\mathrm{N and j}\neq i,\\ \underline{y}+F_{r}\leq y_{i}\leq \bar{y}-F_{r},\\ \underline{x}+F_{r}\leq x_{i}\leq \bar{x}-F_{r}. \end{array}$$

With

$$\begin{array}{c} E(P_{i})=\sum_{n=1}^{h}\xi_{n}\{P_{r}(e^{{-(v_{r}/c_{i}^{’}(0.5(\gamma_{n-1}+\gamma_{n})))}^{k_{i}(0.5(\gamma_{n-1}+\gamma_{n}))}}-e^{{-(v_{\mathrm{co}}/c_{i}^{’}(0.5(\gamma_{n-1}+\gamma_{n})))}^{k_{i}(0.5(\gamma_{n-1}+\gamma_{n}))}})\\ +\sum_{j=1}^{\gamma }\left(e^{{-(v_{j-1}/c_{i}^{’}(0.5(\gamma_{n-1}+\gamma_{n})))}^{k_{i}(0.5(\gamma_{n-1}+\gamma_{n}))}}-e^{{-(v_{j}/c_{i}^{’}(0.5(\gamma_{n-1}+\gamma_{n})))}^{k_{i}(0.5(\gamma_{n-1}+\gamma_{n}))}}\right)\frac{e^{0.5(v_{j-1}+v_{j})}}{\alpha +\beta e^{0.5(v_{j-1}+v_{j})}}\} \end{array}$$

and *ξ_n_* is the frequency of the interval, with values of [*γ_n_*_−1_,*γ_n_*), *F_r_* = 40, *P_r_* = 1500, *v_r_* = 14, *v_co_* = 25, *α* = 6.0268, and *β* = 0.0007.

#### 6.1.5. SOPWM for Three-Level Inverters

SOPWM for Three-level Inverters [76] is a constrained optimization problem within the field of power electronics. SOPWM is a technique designed to control sub-drivers with the aim of enhancing the operational efficiency of inverters. The primary goal is to optimize the switching angle within a fundamental frequency cycle in order to minimize harmonic distortion. The mathematical model varies with the inverter level; this section presents a three-level inverter whose mathematical model is defined as:

(58) Minimize f =∑k(k−4)((∑i=1Np(i)coskβi))2∑kk−4

Subject to:

$$\begin{array}{c} \beta_{i+1}-\beta_{i}-0.00001>0,i=1,2,\ldots ,N-1,\\ n-\sum_{i=1}^{N}p(i)\cos\left(\beta_{i}\right)=0. \end{array}$$

With

$$k=5,7,\ldots ,97,\;p(i)={\left(-1\right)}^{i+1},\;N=\left\lfloor f_{p,\max}/\left(f\;\times \;n\right)\right\rfloor ,\;0\;<\;\beta_{i}\;<\;\frac{\pi }{2},i=1,2,\ldots ,N.$$

### 6.2. Experimental Setup

The parameter settings for the experiments on the actual optimization problems are kept consistent with those in Section 5.1. The results were evaluated comprehensively using five metrics: Mean, Std, Worst, Best, and Rank. Due to the specificity of the MCDE algorithm (approximating the problem variables as a combination of discrete and continuous), it was not fully applicable to these 27 real optimization problems. Therefore, the algorithm was removed from this experiment, and the ability of the remaining 16 algorithms to tackle real-world engineering problems was evaluated.

### 6.3. Analysis of Experimental Results

Table 13 presents the experimental results for the 27 engineering problems. The bold values indicate the best Mean, the lowest Std, and the best Rank for each problem. Overall, SPNOA achieves the lowest Mean Rank of 2.19, followed by AGSK with a Mean Rank of 3.33. This result indicates that SPNOA achieves strong overall performance on the engineering problems considered in this study.

For the industrial chemical process problems (RW01-RW03), SPNOA ranks first on RW01 and RW02 and third on RW03. Its strong performance on the first two problems may be related to its ability to maintain population diversity while strengthening local refinement, which is beneficial for constrained and nonlinear process models. On RW03, SPNOA remains ahead of NOA, which ranks fifth, but does not achieve the best result.

For process synthesis and design problems (RW04-RW09), the relatively low dimensionality allows several algorithms to converge stably to high-quality solutions. In particular, multiple algorithms converge to the known optimum on RW04 and RW08. For mechanical engineering problems (RW10-RW22), SPNOA ranks first on RW10, RW13, RW14, RW16, RW17, RW20, and RW21, and remains within the top three on most of the other problems. The combination of population diversity preservation, nonlinear search control, and mutation-based refinement may help SPNOA explore feasible regions and improve solutions in constrained nonlinear design tasks. RW15 is an exception, where SPNOA ranks sixth. Although SPNOA can obtain a high-quality solution for this problem, its relatively large Std indicates less stable repeated run performance. For the power system problem (RW23), SPNOA ranks third, behind APO and RNEGDE. Although its Mean and Std are better than those of NOA, SPNOA does not outperform all competing algorithms on this problem. This difference may be related to the specific landscape and constraint interactions of the wind farm layout problem, for which the search mechanisms of APO and RNEGDE may be better matched. For the power electronics problems (RW24-RW27), SPNOA ranks first on RW26 and second on RW25 and RW27. Its Mean and Std are also generally better than those of NOA, indicating a consistent improvement over the original algorithm, although not universal superiority over every competitor.

Taken together, the results show that SPNOA improves solution accuracy and stability over NOA in most engineering categories while remaining competitive with the stronger comparison algorithms. Its advantages are most evident in several industrial chemical, mechanical engineering, and power electronics problems. However, the results for RW03, RW15, and RW23 demonstrate that its performance remains problem-dependent and that stability and problem-specific adaptation require further improvement. These cases provide useful directions for future improvements in adaptive parameter adjustment, stability control, and problem-specific constraint handling.

## 7. Conclusions

To address the limitations inherent in NOA, this paper introduces a variant of NOA, known as SPNOA. Four strategies are used in the improved algorithm. First, SRL was introduced at the population initialization stage to gain a more favorable starting population. Then, the inclusion of nonlinear convergence factor and diversity attack strategy enhanced the algorithm’s exploitation capabilities during later iterations, successfully balancing the exploration and exploitation. Ultimately, probability-driven hybrid mutation strategy was employed to assist SPNOA in escaping local optima after each iteration was completed. To understand the effect of SPNOA, a series of experiments were meticulously designed and conducted. First, sensitivity analysis was conducted on three pivotal parameters of SPNOA to determine the optimal values. Then, the different number of newly proposed algorithms, high-performance improved algorithms, and CEC competition-winning algorithms were chosen as comparative algorithms to compare with SPNOA on the CEC2020 test suite. The experimental outcomes indicate that SPNOA holds a significant competitive edge over the other algorithms under comparison. Meanwhile, Friedman test makes the analysis results more credible, and Wilcoxon rank-sum test proves the superiority of SPNOA. Finally, SPNOA’s capability to solve complex optimization problems was evaluated using 27 real engineering problems, with the experimental outcomes further confirming the necessity and superiority of SPNOA.

Although the experimental results show that SPNOA generally outperforms the original NOA, several limitations remain. First, SPNOA integrates multiple strategies. In particular, the probability-driven hybrid mutation strategy increases the number of function calculations per iteration. It raises the overall computational cost of SPNOA. The effectiveness and computational efficiency of SPNOA may degrade when problem dimensionality rises substantially beyond the ranges examined in this study. Second, the current parameter settings are fixed after sensitivity analysis, although the optimal parameter values may vary across different optimization problems. Third, the experimental validation is limited to the CEC2020 test suite and 27 engineering design problems; therefore, the applicability of SPNOA to a broader range of practical engineering tasks requires further investigation. To address these limitations, future work will focus on proposing more efficient strategies and designing adaptive parameter tuning mechanisms. Meanwhile, we will extend SPNOA to more extensive engineering fields, such as microgrid dispatch applications [77], path planning for plant protection UAVs [78], and gear profile dynamic optimization [79].

## Figures and Tables

**Table 1:** Summary of Related Work.

Ref.	Main Modification Strategies	CEC Benchmark Suites	Application Task
[32]	logistic-tent chaos, tangent flight, dynamic fitness-distance balance, criss-cross and Gaussian perturbations	23 benchmark functions	Water conservancy engineering
[33]	Good point set initialization, t-distribution perturbation	No CEC benchmark tests	Antenna phase distribution
[34]	Hybrid initialization, dynamic hierarchical acceptance, elitist SQP, and chaotic lens-imaging opposition learning	CEC2017 test suite	Multi-AUV path planning
[35]	Adaptive parameters, cauchy perturbation, adaptive fitness update, and information sharing	CEC2013, CEC2017	Solar photovoltaic cell parameter extraction
[36]	Bi-population decomposition, random opposition learning, and Q-learning strategy selection.	CEC2014, CEC2017, CEC2020	No application task
[37]	Adaptive weighting and Lévy flight differential evolution operator	No CEC benchmark tests	Valuables transportation routing
[38]	Circle-chaotic initialization, exhaustive discrete search, and migration	No CEC benchmark tests	Energy-saving optimization in chiller plants
[39]	Tournament selection and an enhanced production operator	No CEC benchmark tests	Arabic fake-news detection
[40]	Combined with Chaos Game Optimization	CEC2022	Medical image classification
[41]	Random opposition learning, non-monopolized search, and phased MPA	CEC2017	Aerodynamic parameter identification
[42]	Tent-map initialization and perturbation	No CEC benchmark tests	Green vehicle path planning
[43]	Genetic crossover, mutation-based cache recovery, and adaptive boundary constraints.	No CEC benchmark tests	Multi-UAV path planning
[44]	Cache recovery and dedicated crossover/mutation.	No CEC benchmark tests	UAV swarm task scheduling
[45]	Original NOA	No CEC benchmark tests	Optimization of integrated energy system
[46]	Original NOA	No CEC benchmark tests	Photovoltaic power forecasting
[47]	Original NOA	No CEC benchmark tests	Transient frequency control
[48]	ANFIS hybridized with NOA and Dandelion optimization	No CEC benchmark tests	Groundwater potential mapping
[49]	Original NOA	No CEC benchmark tests	Lung-disease diagnosis

**Table 2:** The main variables in the manuscript.

Symbol	Definition
*N*	Population size
*D*	Problem dimension
*L_j_*, *U_j_*	Lower and upper bounds of the *j*th decision variable
$X_{i,j}^{\mathrm{iter}}$	Value of the *j*th decision variable of the *i*th individual at iteration *iter*th
$X_{\mathrm{best}}^{\mathrm{iter}}$	Best solution found up to iteration *iter*th
$f\left(X_{i}^{\mathrm{iter}}\right)$	Fitness value of the *i*th individual
*t*, *T_max_*	Current number and maximum number of function calculations
*δ*	Coefficient for the bounded update in the foraging phase
*μ*	Step coefficient for position updating
*γ*, *λ*	Lévy flight random number
*l*	Linearly decreasing storage factor
$P_{a_{1}}$	Probability of selecting the foraging or storage phase
*P_rp_*	Probability of exploring other regions during cache search
*α*	Time-varying coefficient for reference-point generation
*θ*	Nutcracker viewpoint angle
${\mathrm{RP}}_{i,1}^{\mathrm{iter}}$, ${\mathrm{RP}}_{i,2}^{\mathrm{iter}}$	Two reference points of the *i*th individual

**Table 3:** Overview of SPNOA and CEC competition-winning algorithms.

Algorithm	Core Strategy	Limitations
IMODE	Quality- and diversity- based sub-population allocation, and linear population reduction	The relative usefulness of different operators varies across problem classes
AGSK	Junior and senior gaining-sharing knowledge phases with an adaptive knowledge factor and knowledge ratio	The two-stage knowledge-sharing process introduces parameter interactions and a mechanism-specific search bias
LSHADE-cnEpSin	Ensemble of sinusoidal approaches with performance adaptation and covariance matrix learning; two sinusoidal waves for scaling-factor adaptation	The additional adaptive and covariance updates make the implementation more involved
SPNOA (this study)	Specular reflection learning, a nonlinear control factor, a diversity attack strategy, and a probability-driven hybrid mutation mechanism.	The additional operators introduce a modest execution-time overhead

**Table 4:** Ablation experiment results on the 10-dimensional CEC2020 test suite.

Fun	NOA	NOA-S	NOA-N	NOA-D	NOA-P	NOA-NDP	NOA-SDP	NOA-SNP	NOA-SND	SPNOA
F1	1.00000 × 10^2^	1.00000 × 10^2^	1.00000 × 10^2^	1.00000 × 10^2^	1.00000 × 10^2^	1.00000 × 10^2^	1.00000 × 10^2^	1.00000 × 10^2^	1.00000 × 10^2^	1.00000 × 10^2^
F2	1.17609 × 10^3^	1.13617 × 10^3^	1.12417 × 10^3^	1.11732 × 10^3^	1.20021 × 10^3^	1.12743 × 10^3^	1.12017 × 10^3^	1.17128 × 10^3^	1.12230 × 10^3^	1.11221 × 10^3^
F3	7.17164 × 10^2^	7.12981 × 10^2^	7.14674 × 10^2^	7.12022 × 10^2^	7.14438 × 10^2^	7.14661 × 10^2^	7.13805 × 10^2^	7.15867 × 10^2^	7.13325 × 10^2^	7.13756 × 10^2^
F4	1.90000 × 10^3^	1.90000 × 10^3^	1.90000 × 10^3^	1.90009 × 10^3^	1.90000 × 10^3^	1.90000 × 10^3^	1.90000 × 10^3^	1.90000 × 10^3^	1.90038 × 10^3^	1.90000 × 10^3^
F5	1.70438 × 10^3^	1.70402 × 10^3^	1.70448 × 10^3^	1.70523 × 10^3^	1.70434 × 10^3^	1.70342 × 10^3^	1.70350 × 10^3^	1.70304 × 10^3^	1.70356 × 10^3^	1.70261 × 10^3^
F6	1.60037 × 10^3^	1.60027 × 10^3^	1.60069 × 10^3^	1.60024 × 10^3^	1.60033 × 10^3^	1.60034 × 10^3^	1.60043 × 10^3^	1.60047 × 10^3^	1.60047 × 10^3^	1.60036 × 10^3^
F7	2.10020 × 10^3^	2.10016 × 10^3^	2.10015 × 10^3^	2.10015 × 10^3^	2.10015 × 10^3^	2.10015 × 10^3^	2.10018 × 10^3^	2.10015 × 10^3^	2.10014 × 10^3^	2.10014 × 10^3^
F8	2.26273 × 10^3^	2.27031 × 10^3^	2.27852 × 10^3^	2.26516 × 10^3^	2.29031 × 10^3^	2.30033 × 10^3^	2.28137 × 10^3^	2.28028 × 10^3^	2.28030 × 10^3^	2.28790 × 10^3^
F9	2.62843 × 10^3^	2.59229 × 10^3^	2.55376 × 10^3^	2.64835 × 10^3^	2.59394 × 10^3^	2.58480 × 10^3^	2.61472 × 10^3^	2.58160 × 10^3^	2.57168 × 10^3^	2.55068 × 10^3^
F10	2.91614 × 10^3^	2.92057 × 10^3^	2.89783 × 10^3^	2.92534 × 10^3^	2.91147 × 10^3^	2.89780 × 10^3^	2.90742 × 10^3^	2.89780 × 10^3^	2.89781 × 10^3^	2.89777 × 10^3^

**Table 5:** Sensitivity analysis of parameter *δ*.

Fun	*δ* = 0	*δ* = 0.05	*δ* = 0.1	*δ* = 0.2	*δ* = 0.3	*δ* = 0.4	*δ* = 0.5	*δ* = 0.6	*δ* = 0.7	*δ* = 0.8	*δ* = 0.9	*δ* = 1
F1	1.00000 × 10^2^	1.00000 × 10^2^	1.00000 × 10^2^	1.00000 × 10^2^	1.00000 × 10^2^	1.00000 × 10^2^	1.00000 × 10^2^	1.00000 × 10^2^	1.00000 × 10^2^	1.00000 × 10^2^	1.00000 × 10^2^	1.00000 × 10^2^
F2	3.28306 × 10^3^	3.21420 × 10^3^	3.14095 × 10^3^	3.22018 × 10^3^	3.38770 × 10^3^	3.21542 × 10^3^	3.22615 × 10^3^	3.24752 × 10^3^	3.26309 × 10^3^	3.24138 × 10^3^	3.29187 × 10^3^	3.31565 × 10^3^
F3	8.00540 × 10^2^	7.95376 × 10^2^	7.95746 × 10^2^	7.92299 × 10^2^	7.96853 × 10^2^	7.95752 × 10^2^	7.92883 × 10^2^	7.93696 × 10^2^	7.97166 × 10^2^	7.87535 × 10^2^	7.93740 × 10^2^	7.93733 × 10^2^
F5	2.29163 × 10^3^	2.25142 × 10^3^	2.22553 × 10^3^	2.15472 × 10^3^	2.33240 × 10^3^	2.39586 × 10^3^	2.44851 × 10^3^	2.26208 × 10^3^	2.37359 × 10^3^	2.26484 × 10^3^	2.19494 × 10^3^	2.26287 × 10^3^
F6	1.70794 × 10^3^	1.69261 × 10^3^	1.69306 × 10^3^	1.71772 × 10^3^	1.71821 × 10^3^	1.71669 × 10^3^	1.71580 × 10^3^	1.69461 × 10^3^	1.69865 × 10^3^	1.70098 × 10^3^	1.67871 × 10^3^	1.69546 × 10^3^
F7	2.31025 × 10^3^	2.31369 × 10^3^	2.36663 × 10^3^	2.35652 × 10^3^	2.32394 × 10^3^	2.29498 × 10^3^	2.33648 × 10^3^	2.33439 × 10^3^	2.34004 × 10^3^	2.31984 × 10^3^	2.31910 × 10^3^	2.29702 × 10^3^
F8	2.30017 × 10^3^	2.30031 × 10^3^	2.30008 × 10^3^	2.30047 × 10^3^	2.30019 × 10^3^	2.30028 × 10^3^	2.30057 × 10^3^	2.30042 × 10^3^	2.30013 × 10^3^	2.30047 × 10^3^	2.30028 × 10^3^	2.30037 × 10^3^
F9	2.88348 × 10^3^	2.87938 × 10^3^	2.88304 × 10^3^	2.88150 × 10^3^	2.88457 × 10^3^	2.88469 × 10^3^	2.87628 × 10^3^	2.87928 × 10^3^	2.88267 × 10^3^	2.88686 × 10^3^	2.88274 × 10^3^	2.88162 × 10^3^
F10	2.88665 × 10^3^	2.88849 × 10^3^	2.88638 × 10^3^	2.88857 × 10^3^	2.88618 × 10^3^	2.88645 × 10^3^	2.88690 × 10^3^	2.88809 × 10^3^	2.88671 × 10^3^	2.88828 × 10^3^	2.88674 × 10^3^	2.88691 × 10^3^
Mean Rank	6.2	4.6	4.4	7.2	8.1	6.5	6.8	6	7.1	7.5	5.2	5.9

**Table 6:** Sensitivity analysis of parameter *P_rp_*.

Fun	*P_rp_* = 0	*P_rp_* = 0.1	*P_rp_* = 0.2	*P_rp_* = 0.3	*P_rp_* = 0.4	*P_rp_* = 0.5	*P_rp_* = 0.6	*P_rp_* = 0.7	*P_rp_* = 0.8	*P_rp_* = 0.9	*P_rp_* = 1
F1	1.00000 × 10^2^	1.00000 × 10^2^	1.00000 × 10^2^	1.00000 × 10^2^	1.00000 × 10^2^	1.00000 × 10^2^	1.00000 × 10^2^	1.00000 × 10^2^	1.00000 × 10^2^	1.00000 × 10^2^	1.00000 × 10^2^
F2	3.45360 × 10^3^	3.04104 × 10^3^	3.20430 × 10^3^	3.50351 × 10^3^	3.57756 × 10^3^	3.52173 × 10^3^	3.70110 × 10^3^	3.49271 × 10^3^	3.67024 × 10^3^	3.52418 × 10^3^	3.50464 × 10^3^
F3	8.07689 × 10^2^	7.83184 × 10^2^	7.89879 × 10^2^	7.97750 × 10^2^	8.00755 × 10^2^	8.03308 × 10^2^	8.07528 × 10^2^	8.08359 × 10^2^	8.13396 × 10^2^	8.04749 × 10^2^	8.06083 × 10^2^
F5	2.47524 × 10^3^	2.40560 × 10^3^	2.48854 × 10^3^	2.31397 × 10^3^	2.34773 × 10^3^	2.46772 × 10^3^	2.30947 × 10^3^	2.36296 × 10^3^	2.48333 × 10^3^	2.53319 × 10^3^	2.40854 × 10^3^
F6	1.71094 × 10^3^	1.68341 × 10^3^	1.67934 × 10^3^	1.72700 × 10^3^	1.69404 × 10^3^	1.74658 × 10^3^	1.74560 × 10^3^	1.75776 × 10^3^	1.71941 × 10^3^	1.72978 × 10^3^	1.74445 × 10^3^
F7	2.34430 × 10^3^	2.30053 × 10^3^	2.30373 × 10^3^	2.34244 × 10^3^	2.36102 × 10^3^	2.31841 × 10^3^	2.31696 × 10^3^	2.34231 × 10^3^	2.36396 × 10^3^	2.36228 × 10^3^	2.38820 × 10^3^
F8	2.30036 × 10^3^	2.30037 × 10^3^	2.30021 × 10^3^	2.30058 × 10^3^	2.30060 × 10^3^	2.30050 × 10^3^	2.30035 × 10^3^	2.30029 × 10^3^	2.30047 × 10^3^	2.30082 × 10^3^	2.30041 × 10^3^
F9	2.89105 × 10^3^	2.88676 × 10^3^	2.88657 × 10^3^	2.87678 × 10^3^	2.88108 × 10^3^	2.87797 × 10^3^	2.88061 × 10^3^	2.87768 × 10^3^	2.87572 × 10^3^	2.88096 × 10^3^	2.87718 × 10^3^
F10	2.88728 × 10^3^	2.88642 × 10^3^	2.88872 × 10^3^	2.88858 × 10^3^	2.88695 × 10^3^	2.88779 × 10^3^	2.88726 × 10^3^	2.88711 × 10^3^	2.88872 × 10^3^	2.88957 × 10^3^	2.88643 × 10^3^
Mean Rank	5.5	**3.8**	4.5	4.6	5.6	6.4	5.6	5	6.7	7.7	5.1

**Table 7:** Sensitivity analysis of parameter *Pa*_2_.

Fun	*Pa*_2_ = 0	*Pa*_2_ = 0.1	*Pa*_2_ = 0.2	*Pa*_2_ = 0.3	*Pa*_2_ = 0.4	*Pa*_2_ = 0.5	*Pa*_2_ = 0.6	*Pa*_2_ = 0.7	*Pa*_2_ = 0.8	*Pa*_2_ = 0.9	*Pa*_2_ = 1
F1	1.000000 × 10^2^	1.000000 × 10^2^	1.000000 × 10^2^	1.000000 × 10^2^	1.000000 × 10^2^	1.000000 × 10^2^	1.000000 × 10^2^	1.000000 × 10^2^	1.000000 × 10^2^	1.000000 × 10^2^	1.000000 × 10^2^
F2	3.26016 × 10^3^	3.25817 × 10^3^	3.14599 × 10^3^	3.21242 × 10^3^	3.21877 × 10^3^	3.24617 × 10^3^	3.25076 × 10^3^	3.47518 × 10^3^	3.31155 × 10^3^	3.56617 × 10^3^	3.87835 × 10^3^
F3	7.98757 × 10^2^	7.98290 × 10^2^	7.92421 × 10^2^	7.91898 × 10^2^	7.87783 × 10^2^	7.90014 × 10^2^	7.86758 × 10^2^	7.91440 × 10^2^	7.86588 × 10^2^	7.83807 × 10^2^	7.81264 × 10^2^
F5	2.33465 × 10^3^	2.39474 × 10^3^	2.39712 × 10^3^	2.17745 × 10^3^	2.48072 × 10^3^	2.80074 × 10^3^	2.70072 × 10^3^	2.97730 × 10^3^	3.20005 × 10^3^	1.15120 × 10^4^	1.10591 × 10^4^
F6	1.68328 × 10^3^	1.68132 × 10^3^	1.70287 × 10^3^	1.69388 × 10^3^	1.70247 × 10^3^	1.68775 × 10^3^	1.72179 × 10^3^	1.72420 × 10^3^	1.74687 × 10^3^	1.77592 × 10^3^	1.77263 × 10^3^
F7	2.32468 × 10^3^	2.32354 × 10^3^	2.30544 × 10^3^	2.30027 × 10^3^	2.33031 × 10^3^	2.30274 × 10^3^	2.37467 × 10^3^	2.33994 × 10^3^	2.34428 × 10^3^	2.60063 × 10^3^	3.32953 × 10^3^
F8	2.30120 × 10^3^	2.30025 × 10^3^	2.30063 × 10^3^	2.30033 × 10^3^	2.30017 × 10^3^	2.30000 × 10^3^	2.30033 × 10^3^	2.30008 × 10^3^	2.30000 × 10^3^	2.30008 × 10^3^	2.30000 × 10^3^
F9	2.88899 × 10^3^	2.88574 × 10^3^	2.88413 × 10^3^	2.87893 × 10^3^	2.87874 × 10^3^	2.88038 × 10^3^	2.87497 × 10^3^	2.87255 × 10^3^	2.87393 × 10^3^	2.87680 × 10^3^	2.86618 × 10^3^
F10	2.88687 × 10^3^	2.88799 × 10^3^	2.88687 × 10^3^	2.88658 × 10^3^	2.88867 × 10^3^	2.88648 × 10^3^	2.88677 × 10^3^	2.88629 × 10^3^	2.88669 × 10^3^	2.88624 × 10^3^	2.88620 × 10^3^
Mean Rank	6.8	6.1	5.9	4.2	5.1	4.2	5.4	5.6	5.6	5.7	5.8

**Table 8:** Experimental data of the CEC2020 test suite (*D* = 10).

Fun	Index	SPNOA	NOA	APO	AROA	BWO	HEOA	RBMO	KOA	HFPSO	EAPSO	ACEPSO	RNEGDE	MCDE	HMRFO	IMODE	AGSK	LSHADE_ cnEpSin
F1	Mean	1.00000 × 10^2^	1.00000 × 10^2^	1.00001 × 10^2^	1.52562 × 10^3^	6.19039 × 10^9^	1.43838 × 10^8^	1.00000 × 10^2^	1.00000 × 10^2^	7.12290 × 10^3^	3.84280 × 10^3^	1.17025 × 10^3^	1.00000 × 10^2^	1.00000 × 10^2^	1.89251 × 10^3^	1.00000 × 10^2^	1.00000 × 10^2^	1.00000 × 10^2^
	Std	0.00000 × 10^0^	5.90074 × 10^−15^	7.03033 × 10^−03^	1.47910 × 10^3^	2.11697 × 10^9^	1.25115 × 10^8^	0.00000 × 10^0^	3.73195 × 10^−15^	4.37984 × 10^3^	3.84326 × 10^3^	1.82862 × 10^3^	2.63889 × 10^−15^	0.00000 × 10^0^	1.59869 × 10^3^	0.00000 × 10^0^	1.86598 × 10^−14^	0.00000 × 10^0^
	Worst	1.00000 × 10^2^	1.00000 × 10^2^	1.00039 × 10^2^	6.11589 × 10^3^	1.03726 × 10^10^	4.99913 × 10^8^	1.00000 × 10^2^	1.00000 × 10^2^	1.27408 × 10^4^	1.16378 × 10^4^	5.60041 × 10^3^	1.00000 × 10^2^	1.00000 × 10^2^	4.93754 × 10^3^	1.00000 × 10^2^	1.00000 × 10^2^	1.00000 × 10^2^
	Best	1.00000 × 10^2^	1.00000 × 10^2^	1.00000 × 10^2^	1.48159 × 10^2^	1.62057 × 10^9^	1.72930 × 10^7^	1.00000 × 10^2^	1.00000 × 10^2^	1.76547 × 10^2^	1.15588 × 10^2^	1.00228 × 10^2^	1.00000 × 10^2^	1.00000 × 10^2^	1.03551 × 10^2^	1.00000 × 10^2^	1.00000 × 10^2^	1.00000 × 10^2^
	Rank	1	8	10	12	17	16	1	7	15	14	11	6	**1**	13	**1**	9	**1**
	Time	0.45	0.38	0.34	0.64	0.45	0.72	1.43	0.33	0.22	1.21	0.94	1.71	0.99	0.14	0.24	0.22	0.92
F2	Mean	1.11221 × 10^3^	1.17609 × 10^3^	1.15550 × 103	1.40074 × 10^3^	2.41048 × 10^3^	2.31483 × 10^3^	1.37448 × 10^3^	1.40942 × 10^3^	1.58218 × 10^3^	1.61919 × 10^3^	1.49044 × 10^3^	1.21973 × 10^3^	1.26477 × 10^3^	1.51116 × 10^3^	1.12160 × 10^3^	1.28105 × 10^3^	1.15095 × 10^3^
	Std	9.84212 × 10^0^	6.72577 × 10^1^	6.03412 × 101	1.57974 × 10^2^	1.61886 × 10^2^	3.01354 × 10^2^	1.62292 × 10^2^	1.28076 × 10^2^	2.31168 × 10^2^	2.39183 × 10^2^	2.01096 × 10^2^	8.72594 × 10^1^	1.29500 × 10^2^	2.81180 × 10^2^	2.35845 × 10^1^	8.73895 × 10^1^	5.93632 × 10^1^
	Worst	1.14347 × 10^3^	1.34742 × 10^3^	1.35556 × 103	1.67801 × 10^3^	2.66362 × 10^3^	2.87206 × 10^3^	1.68455 × 10^3^	1.66677 × 10^3^	2.12515 × 10^3^	2.00662 × 10^3^	1.92165 × 10^3^	1.41131 × 10^3^	1.56105 × 10^3^	1.99850 × 10^3^	1.23356 × 10^3^	1.45631 × 10^3^	1.33323 × 10^3^
	Best	1.10025 × 10^3^	1.10689 × 10^3^	1.10362 × 103	1.11530 × 10^3^	2.02771 × 10^3^	1.45993 × 10^3^	1.11031 × 10^3^	1.19706 × 10^3^	1.13024 × 10^3^	1.11853 × 10^3^	1.10689 × 10^3^	1.10020 × 10^3^	1.10695 × 10^3^	1.11031 × 10^3^	1.10378 × 10^3^	1.14000 × 10^3^	1.10031 × 10^3^
	Rank	1	5	4	10	17	16	9	11	14	15	12	6	7	13	2	8	3
	Time	0.49	0.42	0.39	0.68	0.51	0.75	1.56	0.39	0.26	1.14	0.99	1.60	1.03	0.21	0.38	0.31	1.31
F3	Mean	7.13756 × 10^2^	7.17164 × 10^2^	7.12290 × 10^2^	7.16940 × 10^2^	7.88549 × 10^2^	7.99501 × 10^2^	7.15313 × 10^2^	7.17901 × 10^2^	7.18709 × 10^2^	7.29845 × 10^2^	7.14013 × 10^2^	7.14344 × 10^2^	7.18729 × 10^2^	7.20801 × 10^2^	7.15310 × 10^2^	7.17808 × 10^2^	7.13498 × 10^2^
	Std	1.43527 × 10^0^	3.38804 × 10^0^	7.14783 × 10^1^	1.79910 × 10^0^	9.96636 × 10^0^	1.80895 × 10^1^	2.99350 × 10^0^	2.95256 × 10^0^	2.62894 × 10^0^	8.49234 × 10^0^	1.68424 × 10^0^	1.70752 × 10^0^	4.00160 × 10^0^	5.41518 × 10^0^	1.68074 × 10^0^	2.82361 × 10^0^	1.06328 × 10^0^
	Worst	7.16568 × 10^2^	7.25110 × 10^2^	7.13659 × 10^2^	7.21697 × 10^2^	8.12033 × 10^2^	8.35904 × 10^2^	7.22337 × 10^2^	7.24144 × 10^2^	7.23769 × 10^2^	7.48795 × 10^2^	7.18809 × 10^2^	7.19364 × 10^2^	7.28279 × 10^2^	7.33536 × 10^2^	7.18766 × 10^2^	7.23656 × 10^2^	7.16400 × 10^2^
	Best	7.10987 × 10^2^	7.11846 × 10^2^	7.11182 × 10^2^	7.13527 × 10^2^	7.69365 × 10^2^	7.65332 × 10^2^	7.05579 × 10^2^	7.13860 × 10^2^	7.13005 × 10^2^	7.14473 × 10^2^	7.10676 × 10^2^	7.11712 × 10^2^	7.12609 × 10^2^	7.12684 × 10^2^	7.12569 × 10^2^	7.12665 × 10^2^	7.11536 × 10^2^
	Rank	3	9	1	8	16	17	7	11	12	15	4	5	13	14	6	10	2
	Time	0.46	0.40	0.36	0.67	0.49	0.74	1.49	0.37	0.25	1.08	0.97	1.54	1.01	0.18	0.33	0.28	1.28
F4	Mean	1.90000 × 10^3^	1.90000 × 10^3^	1.90029 × 10^3^	1.90044 × 10^3^	1.90000 × 10^3^	1.90000 × 10^3^	1.90060 × 10^3^	1.90072 × 10^3^	1.90071 × 10^3^	1.90099 × 10^3^	1.90058 × 10^3^	1.90030 × 10^3^	1.90041 × 10^3^	1.90000 × 10^3^	1.90000 × 10^3^	1.90052 × 10^3^	1.90023 × 10^3^
	Std	0.00000 × 10^0^	0.00000 × 10^0^	8.35847 × 10^−2^	3.29164 × 10^−1^	0.00000 × 10^0^	1.82331 × 10^2^	1.22038 × 10^−1^	1.18350 × 10^−1^	2.96687 × 10^−1^	3.93119 × 10^−1^	1.45481 × 10^−1^	5.42392 × 10^−2^	1.73474 × 10^−1^	0.00000 × 10^0^	0.00000 × 10^0^	6.99177 × 10^−2^	5.21350 × 10^−2^
	Worst	1.90000 × 10^3^	1.90000 × 10^3^	1.90045 × 10^3^	1.90112 × 10^3^	1.90000 × 10^3^	1.90010 × 10^3^	1.90091 × 10^3^	1.90096 × 10^3^	1.90165 × 10^3^	1.90187 × 10^3^	1.90093 × 10^3^	1.90043 × 10^3^	1.90089 × 10^3^	1.90000 × 10^3^	1.90000 × 10^3^	1.90065 × 10^3^	1.90034 × 10^3^
	Best	1.90000 × 10^3^	1.90000 × 10^3^	1.90011 × 10^3^	1.90000 × 10^3^	1.90000 × 10^3^	1.90000 × 10^3^	1.90037 × 10^3^	1.90051 × 10^3^	1.90031 × 10^3^	1.90038 × 10^3^	1.90032 × 10^3^	1.90020 × 10^3^	1.90013 × 10^3^	1.90000 × 10^3^	1.90000 × 10^3^	1.90039 × 10^3^	1.90012 × 10^3^
	Rank	1	1	8	11	1	6	14	16	15	17	13	9	10	1	1	12	7
	Time	0.45	0.39	0.35	0.69	0.49	0.74	1.45	0.35	0.23	1.12	0.95	1.61	1.00	0.17	0.09	0.27	1.26
F5	Mean	1.70261 × 10^3^	1.70438 × 10^3^	1.73845 × 10^3^	1.72267 × 10^3^	1.37751 × 10^5^	2.32443 × 10^5^	1.74598 × 10^3^	1.72974 × 10^3^	7.43335 × 10^3^	5.28123 × 10^3^	2.87343 × 10^3^	1.80543 × 10^3^	1.71500 × 10^3^	2.36841 × 10^3^	1.71240 × 10^3^	1.71234 × 10^3^	1.88357 × 10^3^
	Std	3.76300 × 10^0^	4.20222 × 10^0^	4.17842 × 10^1^	2.79111 × 10^1^	9.96895 × 10^4^	1.70900 × 10^5^	3.29348 × 101	2.80148 × 10^1^	4.30465 × 10^3^	1.82108 × 10^3^	1.21851 × 10^3^	7.11176 × 10^1^	1.98184 × 10^1^	3.77126 × 10^2^	7.21779 × 10^0^	9.81572 × 10^0^	1.39998 × 10^2^
	Worst	1.71457 × 10^3^	1.71920 × 10^3^	1.83209 × 10^3^	1.83504 × 10^3^	4.36548 × 10^5^	5.28003 × 10^5^	1.84969 × 103	1.83287 × 10^3^	1.75278 × 10^4^	8.94452 × 10^3^	7.65214 × 10^3^	1.91588 × 10^3^	1.78330 × 10^3^	3.90176 × 10^3^	1.74299 × 10^3^	1.74036 × 10^3^	2.19405 × 10^3^
	Best	1.70003 × 10^3^	1.70023 × 10^3^	1.70141 × 10^3^	1.70200 × 10^3^	4.57717 × 10^3^	6.27640 × 10^3^	1.70565 × 103	1.70361 × 10^3^	1.92755 × 10^3^	2.12206 × 10^3^	1.74506 × 10^3^	1.70288 × 10^3^	1.70000 × 10^3^	1.97887 × 10^3^	1.70199 × 10^3^	1.70287 × 10^3^	1.71435 × 10^3^
	Rank	1	2	8	6	16	17	9	7	15	14	13	10	5	12	4	3	11
	Time	0.48	0.45	0.41	0.78	0.56	0.80	1.58	0.38	0.26	1.24	0.99	1.88	1.17	0.19	0.34	0.32	0.85
F6	Mean	1.60036 × 10^3^	1.60037 × 10^3^	1.60093 × 10^3^	1.60156 × 10^3^	1.85984 × 10^3^	1.86419 × 10^3^	1.63138 × 10^3^	1.60099 × 10^3^	1.75709 × 10^3^	1.69898 × 10^3^	1.77192 × 10^3^	1.62880 × 10^3^	1.61465 × 10^3^	1.65505 × 10^3^	1.60061 × 10^3^	1.60113 × 10^3^	1.62906 × 10^3^
	Std	2.49005 × 10^−1^	1.36672 × 10^−1^	2.18685 × 10^1^	1.63164 × 10^0^	6.79350 × 10^1^	1.01732 × 10^2^	8.90691 × 10^1^	4.28122 × 10^−1^	9.11868 × 10^1^	9.27473 × 10^1^	7.84770 × 10^1^	5.44312 × 10^1^	4.18658 × 10^0^	7.44243 × 10^1^	2.98335 × 10^−1^	3.07281 × 10^−1^	6.02608 × 10^1^
	Worst	1.60098 × 10^3^	1.60105 × 10^3^	1.60235 × 10^3^	1.60449 × 10^3^	1.98348 × 10^3^	2.17742 × 10^3^	1.73511 × 10^3^	1.60193 × 10^3^	1.94957 × 10^3^	1.91191 × 10^3^	1.95830 × 10^3^	1.72102 × 10^3^	1.72009 × 10^3^	1.84048 × 10^3^	1.60141 × 10^3^	1.60146 × 10^3^	1.84047 × 10^3^
	Best	1.60005 × 10^3^	1.60005 × 10^3^	1.60019 × 10^3^	1.60054 × 10^3^	1.69085 × 10^3^	1.71839 × 10^3^	1.60046 × 10^3^	1.60019 × 10^3^	1.60157 × 10^3^	1.60047 × 10^3^	1.60155 × 10^3^	1.60036 × 10^3^	1.60002 × 10^3^	1.60046 × 10^3^	1.60016 × 10^3^	1.60027 × 10^3^	1.60031 × 10^3^
	Rank	1	2	4	7	16	17	11	5	14	13	15	9	8	12	3	6	10
	Time	0.47	0.41	0.37	0.69	0.48	0.76	1.55	0.37	0.25	1.16	0.97	1.59	1.01	0.19	0.34	0.27	1.27
F7	Mean	2.10018 × 10^3^	2.10020 × 10^3^	2.10448 × 10^3^	2.10205 × 10^3^	2.28220 × 10^4^	4.67671 × 10^4^	2.10978 × 10^3^	2.10082 × 10^3^	2.47070 × 10^3^	2.35938 × 10^3^	2.68301 × 10^3^	2.12100 × 10^3^	2.10678 × 10^3^	2.23093 × 10^3^	2.10040 × 10^3^	2.10058 × 10^3^	2.19022 × 10^3^
	Std	2.14594 × 10^−1^	2.24158 × 10^−1^	4.83182 × 10^−1^	7.49843 × 10^−1^	6.88494 × 10^1^	1.12866 × 10^2^	5.48313 × 10^1^	4.71906 × 10^−1^	1.06330 × 10^2^	8.64905 × 10^1^	8.12284 × 10^1^	5.12126 × 10^1^	3.59783 × 10^1^	8.05612 × 10^1^	2.98335 × 10^−1^	2.68251 × 10^−1^	1.02529 × 10^2^
	Worst	2.10065 × 10^3^	2.10064 × 10^3^	2.22091 × 10^3^	2.11763 × 10^3^	5.34068 × 10^4^	2.24175 × 10^5^	2.13009 × 10^3^	2.10218 × 10^3^	3.05540 × 10^3^	2.85361 × 10^3^	4.48685 × 10^3^	2.22105 × 10^3^	2.15852 × 10^3^	2.46590 × 10^3^	2.10082 × 10^3^	2.10112 × 10^3^	2.51670 × 10^3^
	Best	2.10000 × 10^3^	2.10001 × 10^3^	2.10001 × 10^3^	2.10001 × 10^3^	3.28808 × 10^3^	3.08842 × 10^3^	2.10084 × 10^3^	2.10006 × 10^3^	2.10194 × 10^3^	2.10110 × 10^3^	2.18879 × 10^3^	2.10005 × 10^3^	2.10000 × 10^3^	2.10303 × 10^3^	2.10003 × 10^3^	2.10008 × 10^3^	2.10005 × 10^3^
	Rank	1	2	7	6	16	17	9	5	14	13	15	10	8	12	3	4	11
	Time	0.45	0.41	0.35	0.68	0.48	0.73	1.47	0.35	0.23	1.16	0.92	1.53	1.02	0.17	0.32	0.28	1,17
F8	Mean	2.28790 × 10^3^	2.26273 × 10^3^	2.29816 × 10^3^	2.29809 × 10^3^	2.47952 × 10^3^	2.33559 × 10^3^	2.29732 × 10^3^	2.30057 × 10^3^	2.35065 × 10^3^	2.32415 × 10^3^	2.29708 × 10^3^	2.30013 × 10^3^	2.29425 × 10^3^	2.29555 × 10^3^	2.25704 × 10^3^	2.29145 × 10^3^	2.30046 × 10^3^
	Std	3.24386 × 10^1^	4.74006 × 10^1^	1.18123 × 10^1^	1.64188 × 10^1^	1.46772 × 10^2^	9.98707 × 10^1^	1.83831 × 10^1^	3.92139 × 10^−1^	1.36132 × 10^2^	1.51133 × 10^2^	1.83400 × 10^1^	1.98089 × 10^−1^	2.56305 × 10^1^	2.31371 × 10^1^	3.26026 × 10^1^	2.64357 × 10^1^	3.56394 × 10^−1^
	Worst	2.30092 × 10^3^	2.30103 × 10^3^	2.30111 × 10^3^	2.30237 × 10^3^	2.88613 × 10^3^	2.84342 × 10^3^	2.30114 × 10^3^	2.30155 × 10^3^	2.97055 × 10^3^	3.11905 × 10^3^	2.30165 × 10^3^	2.30085 × 10^3^	2.30259 × 10^3^	2.30405 × 10^3^	2.30035 × 10^3^	2.30000 × 10^3^	2.30113 × 10^3^
	Best	2.20000 × 10^3^	2.20000 × 10^3^	2.23564 × 10^3^	2.21120 × 10^3^	2.33694 × 10^3^	2.27012 × 10^3^	2.20000 × 10^3^	2.30000 × 10^3^	2.27769 × 10^3^	2.23149 × 10^3^	2.20000 × 10^3^	2.30000 × 10^3^	2.20000 × 10^3^	2.20000 × 10^3^	2.21120 × 10^3^	2.20000 × 10^3^	2.30000 × 10^3^
	Rank	3	2	10	9	17	15	8	13	16	14	7	11	5	6	1	4	12
	Time	0.56	0.52	0.47	0.84	0.63	0.88	1.73	0.49	0.36	1.23	1.16	1.75	1.12	0.30	0.46	0.40	0.89
F9	Mean	2.55068 × 10^3^	2.62843 × 10^3^	2.71460 × 10^3^	2.72748 × 10^3^	2.68021 × 10^3^	2.74062 × 10^3^	2.74161 × 10^3^	2.65132 × 10^3^	2.74948 × 10^3^	2.74044 × 10^3^	2.59102 × 10^3^	2.72621 × 10^3^	2.70785 × 10^3^	2.62448 × 10^3^	2.48172 × 10^3^	2.50000 × 10^3^	2.71905 × 10^3^
	Std	9.63144 × 10^1^	1.16804 × 10^2^	6.57792 × 10^1^	4.30378 × 10^1^	7.06982 × 10^1^	1.23902 × 10^2^	6.79415 × 10^0^	1.13922 × 10^2^	6.95337 × 10^1^	4.61135 × 10^1^	1.16144 × 10^2^	4.28230 × 10^1^	8.10669 × 10^1^	1.21161 × 10^2^	3.49441 × 10^1^	5.13656 × 10^−13^	5.96136 × 10^1^
	Worst	2.73977 × 10^3^	2.74012 × 10^3^	2.74191 × 10^3^	2.74092 × 10^3^	2.82515 × 10^3^	2.86608 × 10^3^	2.76624 × 10^3^	2.73944 × 10^3^	2.81834 × 10^3^	2.77398 × 10^3^	2.75498 × 10^3^	2.73943 × 10^3^	2.74556 × 10^3^	2.75187 × 10^3^	2.50000 × 10^3^	2.50000 × 10^3^	2.74137 × 10^3^
	Best	2.50000 × 10^3^	2.50000 × 10^3^	2.50000 × 10^3^	2.50001 × 10^3^	2.59551 × 10^3^	2.52104 × 10^3^	2.73390 × 10^3^	2.43592 × 10^3^	2.50000 × 10^3^	2.50000 × 10^3^	2.50000 × 10^3^	2.50002 × 10^3^	2.50000 × 10^3^	2.50000 × 10^3^	2.40000 × 10^3^	2.50000 × 10^3^	2.50000 × 10^3^
	Rank	3	6	10	13	8	15	16	7	17	14	4	12	9	5	1	2	11
	Time	0.61	0.55	0.51	0.84	0.65	0.90	1.82	0.52	0.39	1.18	1.13	1.69	1.17	0.33	0.51	0.43	1.46
F10	Mean	2.89777 × 10^3^	2.91614 × 10^3^	2.92017 × 10^3^	2.92290 × 10^3^	3.14912 × 10^3^	2.94244 × 10^3^	2.91704 × 10^3^	2.91831 × 10^3^	2.93114 × 10^3^	2.92350 × 10^3^	2.92249 × 10^3^	2.92925 × 10^3^	2.91010 × 10^3^	2.93134 × 10^3^	2.89797 × 10^3^	2.89945 × 10^3^	2.93016 × 10^3^
	Std	8.51891 × 10^−2^	2.26436 × 10^1^	2.35166 × 10^1^	2.30680 × 10^1^	7.22635 × 10^1^	3.60842 × 10^1^	2.31516 × 10^1^	2.30316 × 10^1^	2.92793 × 10^1^	3.25490 × 10^1^	2.30994 × 10^1^	2.21093 × 10^1^	2.04516 × 10^1^	2.17382 × 10^1^	5.51051 × 10^−1^	8.29327 × 10^0^	2.13122 × 10^1^
	Worst	2.89808 × 10^3^	2.94345 × 10^3^	2.94957 × 10^3^	2.94596 × 10^3^	3.26108 × 10^3^	3.03872 × 10^3^	2.94597 × 10^3^	2.94591 × 10^3^	2.97202 × 10^3^	2.94941 × 10^3^	2.94586 × 10^3^	2.94619 × 10^3^	2.94353 × 10^3^	2.94725 × 10^3^	2.89958 × 10^3^	2.94336 × 10^3^	2.94607 × 10^3^
	Best	2.89774 × 10^3^	2.89774 × 10^3^	2.89774 × 10^3^	2.89774 × 10^3^	2.97102 × 10^3^	2.83291 × 10^3^	2.89774 × 10^3^	2.89774 × 10^3^	2.89776 × 10^3^	2.80000 × 10^3^	2.89774 × 10^3^	2.89778 × 10^3^	2.89774 × 10^3^	2.89789 × 10^3^	2.89774 × 10^3^	2.89774 × 10^3^	2.89774 × 10^3^
	Rank	1	5	8	10	17	16	6	7	14	11	9	12	4	15	2	3	13
	Time	0.57	0.51	0.44	0.82	0.63	0.82	1.64	0.45	0.33	1.14	1.12	1.71	1.12	0.28	0.43	0.34	0.89
Mean Rank		1.6	4	7	9.2	14.1	15.2	9	8.9	14.6	14	10.3	9	7	10.3	2.4	6.1	8.1

**Table 9:** Experimental data of the CEC2020 test suite (*D* = 20).

Fun	Index	SPNOA	NOA	APO	AROA	BWO	HEOA	RBMO	KOA	HFPSO	EAPSO	ACEPSO	RNEGDE	MCDE	HMRFO	IMODE	AGSK	LSHADE_ cnEpSin
F1	Mean	1.00000 × 10^2^	1.00000 × 10^2^	8.82508 × 10^2^	3.95801 × 10^3^	2.34392 × 10^10^	2.01474 × 10^9^	1.00000 × 10^2^	1.00000 × 10^2^	3.10920 × 10^3^	2.41355 × 10^3^	2.79688 × 10^3^	1.00000 × 10^2^	1.00000 × 10^2^	9.39078 × 10^2^	1.00000 × 10^2^	1.00000 × 10^2^	2.52830 × 10^2^
	Std	2.55850 × 10^−14^	6.76091 × 10^−7^	2.14595 × 10^3^	2.76330 × 10^3^	3.20176 × 10^9^	8.19953 × 10^8^	0.00000 × 10^0^	5.27778 × 10^−15^	3.79915 × 10^3^	2.03043 × 10^3^	2.98564 × 10^3^	1.84722 × 10^−14^	4.57069 × 10^−15^	1.01829 × 10^3^	2.74619 × 10^−5^	1.16111 × 10^−13^	8.37085 × 10^2^
	Worst	1.00000 × 10^2^	1.00000 × 10^2^	9.47112 × 10^3^	1.00529 × 10^4^	2.96882 × 10^10^	3.84776 × 10^9^	1.00000 × 10^2^	1.00000 × 10^2^	1.21918 × 10^4^	7.66656 × 10^3^	1.18075 × 10^4^	1.00000 × 10^2^	1.00000 × 10^2^	4.14520 × 10^3^	1.00000 × 10^2^	1.00000 × 10^2^	4.68490 × 10^3^
	Best	1.00000 × 10^2^	1.00000 × 10^2^	1.00000 × 10^2^	5.16660 × 10^2^	1.31051 × 10^10^	7.74188 × 10^8^	1.00000 × 10^2^	1.00000 × 10^2^	1.00146 × 10^2^	1.89180 × 10^2^	1.00936 × 10^2^	1.00000 × 10^2^	1.00000 × 10^2^	1.00752 × 10^2^	1.00000 × 10^2^	1.00000 × 10^2^	1.00000 × 10^2^
	Rank	5	7	10	15	17	16	1	3	14	12	13	4	2	11	8	6	9
	Time	1.05	0.96	0.73	1.41	1.05	1.57	2.91	0.69	0.60	2.40	1.89	3.33	3.22	0.31	0.44	0.85	2.03
F2	Mean	1.16708 × 10^3^	1.37216 × 10^3^	1.28077 × 10^3^	2.39444 × 10^3^	4.99552 × 10^3^	4.24990 × 10^3^	2.15385 × 10^3^	1.66490 × 10^3^	2.04314 × 10^3^	2.19665 × 10^3^	2.14967 × 10^3^	1.31672 × 10^3^	1.33088 × 10^3^	2.14297 × 10^3^	1.33197 × 10^3^	1.37270 × 10^3^	1.25348 × 10^3^
	Std	8.89361 × 10^1^	1.87144 × 10^2^	1.36922 × 10^2^	5.56499 × 10^2^	2.26906 × 10^2^	6.37134 × 10^2^	4.13886 × 10^2^	2.17668 × 10^2^	3.81648 × 10^2^	4.52675 × 10^2^	3.46766 × 10^2^	1.11510 × 10^2^	2.41778 × 10^2^	4.40263 × 10^2^	9.88320 × 10^1^	7.24281 × 10^1^	1.13059 × 10^2^
	Worst	1.35980 × 10^3^	1.85389 × 10^3^	1.56175 × 10^3^	3.21059 × 10^3^	5.51519 × 10^3^	5.42937 × 10^3^	2.98212 × 10^3^	2.10262 × 10^3^	3.32067 × 10^3^	3.52318 × 10^3^	2.96536 × 10^3^	1.68034 × 10^3^	2.17352 × 10^3^	3.10720 × 10^3^	1.55772 × 10^3^	1.54712 × 10^3^	1.45225 × 10^3^
	Best	1.10363 × 10^3^	1.10186 × 10^3^	1.11396 × 10^3^	1.52954 × 10^3^	4.56338 × 10^3^	3.04098 × 10^3^	1.37630 × 10^3^	1.23453 × 10^3^	1.47158 × 10^3^	1.58227 × 10^3^	1.47306 × 10^3^	1.10852 × 10^3^	1.10863 × 10^3^	1.45211 × 10^3^	1.11531 × 10^3^	1.16626 × 10^3^	1.10192 × 10^3^
	Rank	1	7	3	15	17	16	13	9	10	14	12	4	5	11	6	8	2
	Time	1.29	1.18	0.95	1.63	1.33	1.79	3.40	0.96	0.80	2.31	2.15	3.19	3.23	0.54	0.67	1.21	2.22
F3	Mean	7.26765 × 10^2^	7.28217 × 10^2^	7.26893 × 10^2^	7.36635 × 10^2^	9.88711 × 10^2^	9.65753 × 10^2^	7.48129 × 10^2^	7.33909 × 10^2^	7.49832 × 10^2^	7.64562 × 10^2^	7.26954 × 10^2^	7.28052 × 10^2^	7.28599 × 10^2^	7.73934 × 10^2^	7.23494 × 10^2^	7.31125 × 10^2^	7.27893 × 10^2^
	Std	2.18843 × 10^0^	2.79344 × 10^0^	2.96257 × 10^0^	6.55201 × 10^0^	1.52566 × 10^1^	4.22237 × 10^1^	9.60596 × 10^0^	4.02587 × 10^0^	1.20049 × 10^1^	1.62988 × 10^1^	3.13555 × 10^0^	2.08087 × 10^0^	3.43266 × 10^0^	2.63346 × 10^1^	1.24797 × 10^0^	1.64721 × 10^0^	4.30373 × 10^0^
	Worst	7.31416 × 10^2^	7.36343 × 10^2^	7.34314 × 10^2^	7.57132 × 10^2^	1.03196 × 10^3^	1.05530 × 10^3^	7.76115 × 10^2^	7.42701 × 10^2^	7.71885 × 10^2^	7.93476 × 10^2^	7.36857 × 10^2^	7.33778 × 10^2^	7.37038 × 10^2^	8.51047 × 10^2^	7.26803 × 10^2^	7.35117 × 10^2^	7.33187 × 10^2^
	Best	7.22654 × 10^2^	7.22908 × 10^2^	7.23087 × 10^2^	7.26753 × 10^2^	9.61997 × 10^2^	8.31908 × 10^2^	7.32681 × 10^2^	7.26451 × 10^2^	7.14939 × 10^2^	7.29746 × 10^2^	7.22642 × 10^2^	7.25218 × 10^2^	7.23657 × 10^2^	7.44267 × 10^2^	7.21100 × 10^2^	7.28100 × 10^2^	7.09530 × 10^2^
	Rank	2	7	3	11	17	16	12	10	13	14	4	6	8	15	1	9	5
	Time	1.14	1.01	0.84	1.45	1.28	1.71	3.44	0.89	0.77	2.49	2.18	3.38	3.46	0.45	0.56	1.17	2.09
F4	Mean	1.90000 × 10^3^	1.90000 × 10^3^	1.90092 × 10^3^	1.90125 × 10^3^	1.90000 × 10^3^	1.90010 × 10^3^	1.90195 × 10^3^	1.90236 × 10^3^	1.90165 × 10^3^	1.90414 × 10^3^	1.90119 × 10^3^	1.90098 × 10^3^	1.90106 × 10^3^	1.90000 × 10^3^	1.90007 × 10^3^	1.90144 × 10^3^	1.90061 × 10^3^
	Std	0.00000 × 10^0^	0.00000 × 10^0^	1.80210 × 10^−1^	7.00732 × 10^−1^	0.00000 × 10^0^	3.70682 × 10^−1^	5.81253 × 10^−1^	2.81496 × 10^−1^	3.95939 × 10^−1^	3.29634 × 10^0^	2.54626 × 10^−1^	2.69871 × 10^−1^	2.85335 × 10^−1^	0.00000 × 10^0^	6.17098 × 10^−2^	2.21814 × 10^−1^	9.65500 × 10^−2^
	Worst	1.90000 × 10^3^	1.90000 × 10^3^	1.90123 × 10^3^	1.90324 × 10^3^	1.90000 × 10^3^	1.90187 × 10^3^	1.90319 × 10^3^	1.90294 × 10^3^	1.90235 × 10^3^	1.91624 × 10^3^	1.90183 × 10^3^	1.90204 × 10^3^	1.90162 × 10^3^	1.90000 × 10^3^	1.90022 × 10^3^	1.90194 × 10^3^	1.90077 × 10^3^
	Best	1.90000 × 10^3^	1.90000 × 10^3^	1.90059 × 10^3^	1.90043 × 10^3^	1.90000 × 10^3^	1.90000 × 10^3^	1.90113 × 10^3^	1.90191 × 10^3^	1.90065 × 10^3^	1.90160 × 10^3^	1.90071 × 10^3^	1.90068 × 10^3^	1.90059 × 10^3^	1.90000 × 10^3^	1.90000 × 10^3^	1.90097 × 10^3^	1.90038 × 10^3^
	Rank	1	1	8	12	1	6	15	16	14	17	11	9	10	1	5	13	7
	Time	1.04	0.95	0.81	1.37	1.14	1.62	3.11	0.79	0.66	2.29	2.02	3.02	3.25	0.36	0.47	1.02	2.04
F5	Mean	1.84932 × 10^3^	2.01936 × 10^3^	1.98150 × 10^4^	3.71817 × 10^3^	2.21324 × 10^6^	7.46463 × 10^5^	2.03486 × 10^3^	3.12966 × 10^3^	4.95963 × 10^4^	2.68410 × 10^4^	6.54364 × 10^4^	2.68964 × 10^3^	2.00660 × 10^3^	2.04476 × 10^4^	2.65758 × 10^3^	2.24009 × 10^3^	2.03653 × 10^3^
	Std	9.63381 × 10^1^	1.72053 × 10^2^	2.31101 × 10^4^	4.01474 × 10^3^	7.78420 × 10^5^	4.34404 × 10^5^	1.42193 × 10^2^	1.81100 × 10^3^	4.42329 × 10^4^	1.78100 × 10^4^	3.68286 × 10^4^	5.94348 × 10^2^	1.68802 × 10^2^	1.60423 × 10^4^	3.07130 × 10^2^	1.95095 × 10^2^	1.65134 × 10^2^
	Worst	2.08797 × 10^3^	2.35952 × 10^3^	8.85436 × 10^4^	2.33885 × 10^4^	3.68130 × 10^6^	1.62649 × 10^6^	2.36207 × 10^3^	9.72604 × 10^3^	1.46542 × 10^5^	5.67462 × 10^4^	1.55877 × 10^5^	4.91653 × 10^3^	2.35396 × 10^3^	5.95570 × 10^4^	3.36211 × 10^3^	2.85769 × 10^3^	2.48966 × 10^3^
	Best	1.70168 × 10^3^	1.72432 × 10^3^	2.19251 × 10^3^	1.87162 × 10^3^	9.40681 × 10^5^	8.05389 × 10^4^	1.79722 × 10^3^	1.84687 × 10^3^	3.04926 × 10^3^	3.34858 × 10^3^	1.15144 × 10^4^	1.88154 × 10^3^	1.71357 × 10^3^	5.33177 × 10^3^	2.17811 × 10^3^	1.91707 × 10^3^	1.74428 × 10^3^
	Rank	1	3	11	10	17	16	4	9	14	13	15	8	2	12	7	6	5
	Time	1.31	1.21	1.03	1.63	1.43	1.89	3.59	1.04	0.89	2.52	2.25	3.46	3.48	0.63	0.70	1.30	2.18
F6	Mean	1.60124 × 10^3^	1.60188 × 10^3^	1.60656 × 10^3^	1.62860 × 10^3^	2.83912 × 10^3^	2.29145 × 10^3^	1.77174 × 10^3^	1.60288 × 10^3^	1.90924 × 10^3^	1.85861 × 10^3^	1.89321 × 10^3^	1.62635 × 10^3^	1.60172 × 10^3^	1.64294 × 10^3^	1.60261 × 10^3^	1.60122 × 10^3^	1.60124 × 10^3^
	Std	5.48649 × 10^−1^	3.32447 × 10^−1^	5.96056 × 10^0^	3.87956 × 10^1^	1.55586 × 10^2^	2.53887 × 10^2^	1.50283 × 10^2^	4.49464 × 10^0^	1.20518 × 10^2^	1.70658 × 10^2^	1.27719 × 10^2^	4.83342 × 10^1^	1.75453 × 10^0^	7.18484 × 10^1^	5.67646 × 10^−1^	3.33338 × 10^−1^	4.12366 × 10^−1^
	Worst	1.60237 × 10^3^	1.60233 × 10^3^	1.62717 × 10^3^	1.73458 × 10^3^	3.13956 × 10^3^	2.85940 × 10^3^	2.09410 × 10^3^	1.62591 × 10^3^	2.09044 × 10^3^	2.25370 × 10^3^	2.08732 × 10^3^	1.72586 × 10^3^	1.60759 × 10^3^	1.83962 × 10^3^	1.60381 × 10^3^	1.60187 × 10^3^	1.60208 × 10^3^
	Best	1.60033 × 10^3^	1.60108 × 10^3^	1.60107 × 10^3^	1.60273 × 10^3^	2.50169 × 10^3^	1.86630 × 10^3^	1.60348 × 10^3^	1.60093 × 10^3^	1.72125 × 10^3^	1.60218 × 10^3^	1.72064 × 10^3^	1.60120 × 10^3^	1.60016 × 10^3^	1.60129 × 10^3^	1.60167 × 10^3^	1.60064 × 10^3^	1.60049 × 10^3^
	Rank	3	5	8	10	17	16	12	7	15	13	14	9	4	11	6	1	2
	Time	1.15	1.04	0.90	1.54	1.23	1.72	3.44	0.85	0.73	2.37	2.19	3.03	3.29	0.43	0.55	1.11	2.27
F7	Mean	2.14350 × 10^3^	2.24810 × 10^3^	2.67966 × 10^3^	2.78683 × 10^3^	5.89590 × 10^5^	3.22789 × 10^5^	2.40495 × 10^3^	2.35317 × 10^3^	1.45126 × 10^4^	6.84893 × 10^3^	2.25808 × 10^4^	5.87671 × 10^3^	2.24819 × 10^3^	8.28882 × 10^3^	2.69516 × 10^3^	2.28912 × 10^3^	2.15547 × 10^3^
	Std	5.04669 × 10^1^	7.44156 × 10^1^	8.07632 × 10^2^	1.08857 × 10^3^	2.67906 × 10^5^	2.11037 × 10^5^	1.50969 × 10^2^	4.46977 × 10^2^	1.00978 × 10^4^	3.15347 × 10^3^	1.84729 × 10^4^	1.82793 × 10^4^	9.65755 × 10^1^	6.94291 × 10^3^	2.26269 × 10^2^	1.02047 × 10^2^	4.79224 × 10^1^
	Worst	2.26109 × 10^3^	2.39695 × 10^3^	6.16588 × 10^3^	6.80294 × 10^3^	1.11025 × 10^6^	1.02383 × 10^6^	2.76769 × 10^3^	4.45529 × 10^3^	4.79311 × 10^4^	1.38213 × 10^4^	7.13894 × 10^4^	1.02636 × 10^5^	2.50802 × 10^3^	3.52610 × 10^4^	3.27441 × 10^3^	2.56453 × 10^3^	2.29980 × 10^3^
	Best	2.10095 × 10^3^	2.11903 × 10^3^	2.14803 × 10^3^	2.12085 × 10^3^	1.14791 × 10^5^	4.17314 × 10^4^	2.19373 × 10^3^	2.10308 × 10^3^	2.58302 × 10^3^	3.29017 × 10^3^	3.08890 × 10^3^	2.13897 × 10^3^	2.10538 × 10^3^	3.39244 × 10^3^	2.29016 × 10^3^	2.13537 × 10^3^	2.10311 × 10^3^
	Rank	1	3	8	10	17	16	7	6	14	12	15	11	4	13	9	5	2
	Time	1.31	1.19	0.97	1.73	1.40	1.88	3.67	0.98	0.85	2.56	2.31	3.37	3.76	0.55	0.65	1.25	2.15
F8	Mean	2.29798 × 10^3^	2.30018 × 10^3^	2.30035 × 10^3^	2.30145 × 10^3^	4.78434 × 10^3^	2.80150 × 10^3^	2.63595 × 10^3^	2.30056 × 10^3^	2.54096 × 10^3^	3.32071 × 10^3^	2.42872 × 10^3^	2.41367 × 10^3^	2.29846 × 10^3^	2.30065 × 10^3^	2.30044 × 10^3^	2.44168 × 10^3^	2.30000 × 10^3^
	Std	1.16989 × 10^1^	4.60932 × 10^−1^	5.98170 × 10^−1^	7.67092 × 10^−1^	2.98900 × 10^2^	1.00788 × 10^3^	7.09861 × 10^2^	7.16737 × 10^−1^	6.37621 × 10^2^	1.22592 × 10^3^	5.00563 × 10^2^	4.32004 × 10^2^	8.64104 × 10^0^	6.74942 × 10^−1^	6.49639 × 10^−1^	5.40299 × 10^2^	0.00000 × 10^0^
	Worst	2.30115 × 10^3^	2.30148 × 10^3^	2.30189 × 10^3^	2.30366 × 10^3^	5.27843 × 10^3^	6.21331 × 10^3^	4.83718 × 10^3^	2.30203 × 10^3^	4.49989 × 10^3^	5.41025 × 10^3^	4.63189 × 10^3^	4.07749 × 10^3^	2.30112 × 10^3^	2.30175 × 10^3^	2.30200 × 10^3^	4.64399 × 10^3^	2.30000 × 10^3^
	Best	2.23607 × 10^3^	2.30000 × 10^3^	2.30000 × 10^3^	2.30014 × 10^3^	3.91242 × 10^3^	2.36694 × 10^3^	2.30000 × 10^3^	2.30000 × 10^3^	2.30000 × 10^3^	2.27282 × 10^3^	2.30000 × 10^3^	2.30000 × 10^3^	2.25272 × 10^3^	2.30000 × 10^3^	2.30000 × 10^3^	2.30000 × 10^3^	2.30000 × 10^3^
	Rank	1	4	5	9	17	15	14	7	13	16	11	10	2	8	6	12	3
	Time	1.52	1.45	1.27	1.87	1.73	2.12	3.96	1.20	1.13	2.36	2.54	3.67	3.47	0.88	1.03	1.37	2.50
F9	Mean	2.80730 × 10^3^	2.82265 × 10^3^	2.81723 × 10^3^	2.82695 × 10^3^	3.15365 × 10^3^	3.07437 × 10^3^	2.83431 × 10^3^	2.82247 × 10^3^	2.89755 × 10^3^	2.85750 × 10^3^	2.83156 × 10^3^	2.81373 × 10^3^	2.83148 × 10^3^	2.83159 × 10^3^	2.50675 × 10^3^	2.80913 × 10^3^	2.84741 × 10^3^
	Std	8.42842 × 10^1^	6.08941 × 10^0^	6.97290 × 10^0^	9.54280 × 10^0^	4.96583 × 10^1^	6.25908 × 10^1^	1.34598 × 10^1^	9.15006 × 10^0^	3.79162 × 10^1^	2.43877 × 10^1^	6.54922 × 10^1^	7.00483 × 10^0^	1.49152 × 10^1^	1.58721 × 10^1^	2.56770 × 10^1^	9.77712 × 10^1^	1.25300 × 10^1^
	Worst	2.85085 × 10^3^	2.83708 × 10^3^	2.83309 × 10^3^	2.85164 × 10^3^	3.27583 × 10^3^	3.23167 × 10^3^	2.86694 × 10^3^	2.85093 × 10^3^	2.97970 × 10^3^	2.93139 × 10^3^	2.88008 × 10^3^	2.83671 × 10^3^	2.86697 × 10^3^	2.88025 × 10^3^	2.60142 × 10^3^	2.87337 × 10^3^	2.87071 × 10^3^
	Best	2.50000 × 10^3^	2.81074 × 10^3^	2.80251 × 10^3^	2.81118 × 10^3^	3.04535 × 10^3^	2.99428 × 10^3^	2.81816 × 10^3^	2.80991 × 10^3^	2.84179 × 10^3^	2.81993 × 10^3^	2.50000 × 10^3^	2.80469 × 10^3^	2.81152 × 10^3^	2.80930 × 10^3^	2.50000 × 10^3^	2.50000 × 10^3^	2.82035 × 10^3^
	Rank	2	7	5	8	17	16	12	6	15	14	10	4	9	11	1	3	13
	Time	1.87	1.84	1.50	2.18	1.93	2.44	4.59	1.49	1.35	2.53	3.09	3.88	4.31	1.08	1.26	1.82	2.90
F10	Mean	2.91478 × 10^3^	2.92980 × 10^3^	2.94885 × 10^3^	2.94000 × 10^3^	4.20383 × 10^3^	3.09749 × 10^3^	2.93703 × 10^3^	2.93786 × 10^3^	2.94286 × 10^3^	2.93588 × 10^3^	2.93397 × 10^3^	2.93057 × 10^3^	2.91641 × 10^3^	2.96255 × 10^3^	2.91913 × 10^3^	2.91077 × 10^3^	2.90632 × 10^3^
	Std	9.13568 × 10^0^	2.91359 × 10^1^	3.02101 × 10^1^	2.92416 × 10^1^	3.34736 × 10^2^	6.62935 × 10^1^	2.89150 × 10^1^	3.22489 × 10^1^	3.17900 × 10^1^	2.97275 × 10^1^	2.67084 × 10^1^	2.91899 × 10^1^	1.17736 × 10^1^	2.53942 × 10^1^	1.61526 × 10^1^	1.11048 × 10^1^	1.07731 × 10^0^
	Worst	2.95929 × 10^3^	2.99376 × 10^3^	2.99327 × 10^3^	2.99519 × 10^3^	4.84590 × 10^3^	3.28850 × 10^3^	2.99434 × 10^3^	3.00080 × 10^3^	2.99656 × 10^3^	3.01564 × 10^3^	2.97251 × 10^3^	2.99433 × 10^3^	2.95955 × 10^3^	2.99732 × 10^3^	2.96100 × 10^3^	2.95931 × 10^3^	2.90679 × 10^3^
	Best	2.89911 × 10^3^	2.90304 × 10^3^	2.91076 × 10^3^	2.90756 × 10^3^	3.55813 × 10^3^	2.99818 × 10^3^	2.91032 × 10^3^	2.91065 × 10^3^	2.90273 × 10^3^	2.91020 × 10^3^	2.89957 × 10^3^	2.91087 × 10^3^	2.91006 × 10^3^	2.91384 × 10^3^	2.90250 × 10^3^	2.89907 × 10^3^	2.90063 × 10^3^
	Rank	3	6	14	12	17	16	10	11	13	9	8	7	4	15	5	2	**1**
	Time	1.64	1.55	1.30	2.06	1.75	2.25	4.12	1.21	1.13	2.56	2.75	3.76	3.98	0.86	0.97	1.46	2.98
Mean Rank		**2**	5	7.5	11.2	15.4	14.9	10	8.2	13.5	13.4	11.3	7.2	5	10.8	5.4	6.5	4.9

**Table 10:** Experimental data of the CEC2020 test suite (*D* = 50).

Fun	Index	SPNOA	NOA	APO	AROA	BWO	HEOA	RBMO	KOA	HFPSO	EAPSO	ACEPSO	RNEGDE	MCDE	HMRFO	IMODE	AGSK	LSHADE_ cnEpSin
F1	Mean	1.70207 × 10^3^	1.65548 × 10^3^	2.57235 × 10^3^	2.66306 × 10^6^	9.65177 × 10^10^	1.65881 × 10^10^	4.84968 × 10^3^	1.00030 × 10^2^	5.73366 × 10^3^	5.03973 × 10^3^	8.61904 × 10^3^	1.00000 × 10^2^	2.90612 × 10^6^	3.25071 × 10^3^	1.39785 × 10^8^	2.32051 × 10^3^	4.19415 × 10^3^
	Std	1.66071 × 10^3^	2.24208 × 10^3^	3.05609 × 10^3^	1.05126 × 10^6^	3.88347 × 10^9^	4.14492 × 10^9^	5.47640 × 10^3^	7.08702 × 10^−2^	7.57651 × 10^3^	4.93701 × 10^3^	9.84488 × 10^3^	4.91040 × 10^−9^	4.97678 × 10^5^	4.32967 × 10^3^	5.32779 × 10^8^	4.04922 × 10^3^	4.00067 × 10^3^
	Worst	6.83565 × 10^3^	1.01693 × 10^4^	1.46168 × 10^4^	6.01648 × 10^6^	1.05225 × 10^11^	2.80454 × 10^10^	1.92836 × 10^4^	1.00284 × 10^2^	3.18637 × 10^4^	1.89610 × 10^4^	3.18954 × 10^4^	1.00000 × 10^2^	4.51665 × 10^6^	1.90011 × 10^4^	2.52828 × 10^9^	2.00129 × 10^4^	1.44810 × 10^4^
	Best	2.16950 × 10^2^	1.02767 × 10^2^	1.00010 × 10^2^	1.34070 × 10^6^	8.83560 × 10^10^	9.89075 × 10^9^	1.00273 × 10^2^	1.00000 × 10^2^	1.01472 × 10^2^	1.01146 × 10^2^	1.19503 × 10^2^	1.00000 × 10^2^	2.34896 × 10^6^	1.26802 × 10^2^	8.10183 × 10^2^	1.00000 × 10^2^	1.68119 × 10^2^
	Rank	3	4	6	13	17	16	9	2	11	10	12	1	14	7	15	5	8
	Time	4.08	3.66	2.79	4.71	4.79	5.93	9.94	2.61	4.19	7.04	6.53	10.76	19.69	1.89	14.09	3.92	9.11
F2	Mean	5.54916 × 10^3^	6.11952 × 10^3^	5.93751 × 10^3^	7.91905 × 10^3^	1.43599 × 10^4^	1.31326 × 10^4^	7.47889 × 10^3^	1.02258 × 10^4^	7.05597 × 10^3^	7.68190 × 10^3^	5.82449 × 10^3^	5.88142 × 10^3^	7.68606 × 10^3^	8.07105 × 10^3^	6.90555 × 10^3^	8.96206 × 10^3^	5.05421 × 10^3^
	Std	6.18600 × 10^2^	5.37725 × 10^2^	6.50402 × 10^2^	2.03911 × 10^3^	3.92298 × 10^2^	8.69665 × 10^2^	6.96590 × 10^2^	3.88523 × 10^2^	8.68416 × 10^2^	1.11676 × 10^3^	8.49013 × 10^2^	4.53052 × 10^2^	1.51416 × 10^3^	7.87715 × 10^2^	5.88597 × 10^2^	3.49249 × 10^2^	2.77670 × 10^2^
	Worst	7.00649 × 10^3^	7.32119 × 10^3^	7.19643 × 10^3^	1.24243 × 10^4^	1.50295 × 10^4^	1.48627 × 10^4^	8.82553 × 10^3^	1.09379 × 10^4^	8.57068 × 10^3^	1.02766 × 10^4^	7.70141 × 10^3^	6.46660 × 10^3^	1.12139 × 10^4^	9.50606 × 10^3^	7.89642 × 10^3^	9.69932 × 10^3^	5.67014 × 10^3^
	Best	4.10350 × 10^3^	4.61270 × 10^3^	4.81609 × 10^3^	4.93033 × 10^3^	1.35359 × 10^4^	1.12569 × 10^4^	5.94686 × 10^3^	9.11358 × 10^3^	4.60970 × 10^3^	5.19811 × 10^3^	4.35596 × 10^3^	4.73745 × 10^3^	4.58873 × 10^3^	6.37449 × 10^3^	5.52636 × 10^3^	8.31417 × 10^3^	4.60764 × 10^3^
	Rank	2	6	5	12	17	16	9	15	8	10	3	4	11	13	7	14	1
	Time	5.62	5.38	4.64	6.31	6.73	7.94	14.06	4.58	5.51	7.37	8.62	12.25	21.97	3.25	15.15	5.99	14.19
F3	Mean	8.85266 × 10^2^	9.08764 × 10^2^	9.13227 × 10^2^	9.09767 × 10^2^	1.88088 × 10^3^	1.89290 × 10^3^	9.10099 × 10^2^	9.96105 × 10^2^	9.29621 × 10^2^	1.11436 × 10^3^	7.88764 × 10^2^	8.90971 × 10^2^	9.29562 × 10^2^	9.32777 × 10^2^	1.85325 × 10^3^	1.01571 × 10^3^	9.47484 × 10^2^
	Std	3.12587 × 10^1^	3.49832 × 10^1^	3.35757 × 10^1^	3.55811 × 10^1^	3.33064 × 10^1^	9.82482 × 10^1^	4.60088 × 10^1^	6.13946 × 10^1^	3.41092 × 10^1^	8.54473 × 10^1^	8.91968 × 10^0^	2.97153 × 10^1^	3.44755 × 10^1^	3.55156 × 10^1^	1.76795 × 10^2^	2.74539 × 10^1^	6.28141 × 10^1^
	Worst	9.64955 × 10^2^	1.03781 × 10^3^	9.76956 × 10^2^	9.99095 × 10^2^	1.94284 × 10^3^	2.05920 × 10^3^	9.88202 × 10^2^	1.16214 × 10^3^	1.00866 × 10^3^	1.28166 × 10^3^	8.07091 × 10^2^	9.58525 × 10^2^	9.90255 × 10^2^	9.94167 × 10^2^	2.16873 × 10^3^	1.06888 × 10^3^	1.12715 × 10^3^
	Best	8.34773 × 10^2^	8.59993 × 10^2^	8.49664 × 10^2^	8.46415 × 10^2^	1.79586 × 10^3^	1.74056 × 10^3^	8.38681 × 10^2^	8.80680 × 10^2^	8.62852 × 10^2^	9.33982 × 10^2^	7.71945 × 10^2^	8.37843 × 10^2^	8.80207 × 10^2^	8.57635 × 10^2^	1.47118 × 10^3^	9.63041 × 10^2^	8.59913 × 10^2^
	Rank	2	4	7	5	16	17	6	12	9	14	1	3	8	10	15	13	11
	Time	4.73	4.59	3.40	5.32	5.91	7.00	11.77	3.41	4.64	6.40	7.79	10.67	19.56	2.55	13.93	4.56	13.05
F4	Mean	1.90000 × 10^3^	1.90000 × 10^3^	1.90523 × 10^3^	1.90521 × 10^3^	1.90000 × 10^3^	1.90137 × 10^3^	1.90924 × 10^3^	1.91306 × 10^3^	1.90747 × 10^3^	1.94092 × 10^3^	1.90265 × 10^3^	1.91278 × 10^3^	1.90676 × 10^3^	1.90000 × 10^3^	2.16171 × 10^3^	1.91024 × 10^3^	1.90923 × 10^3^
	Std	0.00000 × 10^0^	0.00000 × 10^0^	2.51409 × 10^0^	2.14873 × 10^0^	0.00000 × 10^0^	4.31929 × 10^0^	2.82229 × 10^0^	1.86738 × 10^0^	1.51964 × 10^0^	2.40913 × 10^1^	3.13717 × 10^−1^	3.98159 × 10^0^	1.45907 × 10^0^	0.00000 × 10^0^	1.78912 × 10^2^	1.03714 × 10^0^	9.14346 × 10^0^
	Worst	1.90000 × 10^3^	1.90000 × 10^3^	1.90957 × 10^3^	1.91033 × 10^3^	1.90000 × 10^3^	1.91806 × 10^3^	1.91656 × 10^3^	1.91690 × 10^3^	1.91061 × 10^3^	2.03761 × 10^3^	1.90331 × 10^3^	1.92154 × 10^3^	1.91016 × 10^3^	1.90000 × 10^3^	2.60015 × 10^3^	1.91236 × 10^3^	1.94148 × 10^3^
	Best	1.90000 × 10^3^	1.90000 × 10^3^	1.90000 × 10^3^	1.90178 × 10^3^	1.90000 × 10^3^	1.90000 × 10^3^	1.90403 × 10^3^	1.90965 × 10^3^	1.90514 × 10^3^	1.91747 × 10^3^	1.90199 × 10^3^	1.90506 × 10^3^	1.90447 × 10^3^	1.90000 × 10^3^	1.94119 × 10^3^	1.90791 × 10^3^	1.90268 × 10^3^
	Rank	1	1	8	7	1	5	12	15	10	16	6	14	9	1	17	13	11
	Time	3.86	3.67	2.76	4.33	4.70	5.85	9.91	2.78	4.09	6.37	6.52	9.11	16.72	1.59	13.27	3.81	12.62
F5	Mean	2.31068 × 10^4^	5.11566 × 10^4^	2.75612 × 10^5^	7.97337 × 10^5^	2.38442 × 10^8^	3.75363 × 10^7^	1.00411 × 10^4^	4.60803 × 10^4^	1.52299 × 10^5^	4.99634 × 10^4^	5.33483 × 10^5^	7.77218 × 10^3^	2.75117 × 10^5^	2.36626 × 10^5^	1.38170 × 10^6^	6.24630 × 10^4^	6.92980 × 10^3^
	Std	1.31571 × 10^4^	3.49311 × 10^4^	1.43595 × 10^5^	6.51040 × 10^5^	6.19999 × 10^7^	1.49760 × 10^7^	4.28705 × 10^3^	2.41215 × 10^4^	1.01108 × 10^5^	2.53790 × 10^4^	3.95664 × 10^5^	4.08845 × 10^3^	1.47732 × 10^5^	1.17568 × 10^5^	5.76793 × 10^6^	4.55282 × 10^4^	1.55962 × 10^3^
	Worst	5.48141 × 10^4^	1.76164 × 10^5^	7.81325 × 10^5^	2.69474 × 10^6^	3.46181 × 10^8^	7.80762 × 10^7^	1.97001 × 10^4^	1.03498 × 10^5^	4.31346 × 10^5^	1.37853 × 10^5^	1.77899 × 10^6^	2.16708 × 10^4^	5.91157 × 10^5^	5.21840 × 10^5^	2.98244 × 10^7^	2.18809 × 10^5^	1.07284 × 10^4^
	Best	5.31623 × 10^3^	8.85991 × 10^3^	1.07229 × 10^5^	5.01619 × 10^4^	1.02181 × 10^8^	1.12264 × 10^7^	4.47723 × 10^3^	9.41792 × 10^3^	2.02577 × 10^4^	2.00982 × 10^4^	1.14075 × 10^5^	4.01106 × 10^3^	8.29752 × 10^4^	3.98826 × 10^4^	3.10494 × 10^3^	2.12125 × 10^4^	4.65423 × 10^3^
	Rank	4	7	12	14	17	16	3	5	9	6	13	2	11	10	15	8	1
	Time	6.41	6.14	5.12	6.99	7.23	8.13	14.15	4.92	6.57	8.15	9.15	12.63	20.32	4.15	14.90	6.33	10.64
F6	Mean	1.92500 × 10^3^	2.01471 × 10^3^	2.21677 × 10^3^	2.13008 × 10^3^	7.32545 × 10^3^	5.31310 × 10^3^	2.76234 × 10^3^	2.55816 × 10^3^	2.45491 × 10^3^	3.02857 × 10^3^	2.41898 × 10^3^	2.17202 × 10^3^	2.46558 × 10^3^	2.54914 × 10^3^	3.15999 × 10^3^	2.63871 × 10^3^	2.04248 × 10^3^
	Std	1.31661 × 10^2^	1.45637 × 10^2^	1.70261 × 10^2^	1.50294 × 10^2^	3.94926 × 10^2^	7.51024 × 10^2^	3.52203 × 10^2^	2.37616 × 10^2^	3.01970 × 10^2^	4.95303 × 10^2^	2.34790 × 10^2^	1.59872 × 10^2^	2.69917 × 10^2^	3.91122 × 10^2^	4.55390 × 10^2^	1.37926 × 10^2^	1.10617 × 10^2^
	Worst	2.16266 × 10^3^	2.31261 × 10^3^	2.70865 × 10^3^	2.49863 × 10^3^	8.03115 × 10^3^	7.65090 × 10^3^	3.28405 × 10^3^	2.93910 × 10^3^	3.13846 × 10^3^	4.13474 × 10^3^	2.89812 × 10^3^	2.51164 × 10^3^	3.19894 × 10^3^	3.85294 × 10^3^	4.15718 × 10^3^	2.82571 × 10^3^	2.26490 × 10^3^
	Best	1.64052 × 10^3^	1.76092 × 10^3^	1.93199 × 10^3^	1.88518 × 10^3^	6.68170 × 10^3^	3.99158 × 10^3^	1.95244 × 10^3^	2.12176 × 10^3^	1.83827 × 10^3^	2.30448 × 10^3^	1.90893 × 10^3^	1.91094 × 10^3^	1.91782 × 10^3^	1.90257 × 10^3^	2.24108 × 10^3^	2.33188 × 10^3^	1.80561 × 10^3^
	Rank	1	2	6	4	17	16	13	11	8	14	7	5	9	10	15	12	3
	Time	4.89	4.58	3.59	5.49	5.89	6.78	11.89	3.45	4.98	7.45	7.49	10.45	19.02	2.53	14.52	4.90	13.45
F7	Mean	8.38597 × 10^3^	9.76120 × 10^3^	2.33257 × 10^5^	2.79766 × 10^5^	3.07802 × 10^7^	8.27686 × 10^6^	4.90139 × 10^3^	3.91517 × 10^4^	7.81544 × 10^4^	4.16978 × 10^4^	2.75318 × 10^5^	4.97854 × 10^3^	1.42122 × 10^5^	1.42769 × 10^5^	3.75783 × 10^3^	2.62347 × 10^4^	4.60749 × 10^3^
	Std	6.03337 × 10^3^	1.43607 × 10^4^	1.30183 × 10^5^	2.44354 × 10^5^	1.13181 × 10^7^	2.31501 × 10^6^	1.32716 × 10^3^	3.40150 × 10^4^	4.28897 × 10^4^	3.01860 × 10^4^	1.29972 × 10^5^	2.23021 × 10^3^	9.29954 × 10^4^	7.27765 × 10^4^	3.86392 × 10^2^	1.95035 × 10^4^	8.00868 × 10^2^
	Worst	2.33161 × 10^4^	7.16615 × 10^4^	7.23912 × 10^5^	1.24359 × 10^6^	5.46328 × 10^7^	1.38772 × 10^7^	9.53824 × 10^3^	1.28748 × 10^5^	1.48474 × 10^5^	1.14809 × 10^5^	5.58311 × 10^5^	1.33088 × 10^4^	4.09534 × 10^5^	3.37324 × 10^5^	4.65879 × 10^3^	9.25313 × 10^4^	7.97903 × 10^3^
	Best	3.00440 × 10^3^	3.11057 × 10^3^	3.12059 × 10^4^	4.02683 × 10^4^	1.11675 × 10^7^	3.39135 × 10^6^	3.45458 × 10^3^	5.05054 × 10^3^	8.32499 × 10^3^	5.01495 × 10^3^	3.81618 × 10^4^	3.40811 × 10^3^	1.90330 × 10^4^	4.64418 × 10^4^	2.99352 × 10^3^	6.05053 × 10^3^	3.61997 × 10^3^
	Rank	5	6	13	15	17	16	3	8	10	9	14	4	11	12	1	7	2
	Time	4.55	4.34	3.38	5.00	5.61	6.56	10.94	3.34	4.61	6.64	7.06	10.32	16.99	2.45	13.35	4.50	10.31
F8	Mean	5.98791 × 10^3^	6.73663 × 10^3^	7.30580 × 10^3^	9.33024 × 10^3^	1.59808 × 10^4^	1.42560 × 10^4^	8.89465 × 10^3^	1.02359 × 10^4^	8.26131 × 10^3^	9.11673 × 10^3^	6.68337 × 10^3^	7.27782 × 10^3^	8.79349 × 10^3^	3.25996 × 10^3^	8.52447 × 10^3^	1.02602 × 10^4^	6.58972 × 10^3^
	Std	2.49665 × 10^3^	2.09473 × 10^3^	1.10948 × 10^3^	2.27030 × 10^3^	4.32563 × 10^2^	1.03542 × 10^3^	7.44236 × 10^2^	3.23187 × 10^3^	1.75255 × 10^3^	9.13370 × 10^2^	1.90841 × 10^3^	1.40721 × 10^3^	1.51411 × 10^3^	2.49153 × 10^3^	8.77502 × 10^2^	1.53768 × 10^3^	9.50852 × 10^2^
	Worst	8.64676 × 10^3^	8.88042 × 10^3^	8.65474 × 10^3^	1.32013 × 10^4^	1.67732 × 10^4^	1.69361 × 10^4^	1.04218 × 10^4^	1.24010 × 10^4^	1.02628 × 10^4^	1.12762 × 10^4^	8.67388 × 10^3^	8.43274 × 10^3^	1.08464 × 10^4^	1.00535 × 10^4^	1.02663 × 10^4^	1.12366 × 10^4^	7.66711 × 10^3^
	Best	2.30000 × 10^3^	2.30000 × 10^3^	2.30000 × 10^3^	2.31518 × 10^3^	1.50672 × 10^4^	1.28966 × 10^4^	7.42591 × 10^3^	2.30000 × 10^3^	2.30000 × 10^3^	7.41290 × 10^3^	2.30001 × 10^3^	2.30112 × 10^3^	2.31661 × 10^3^	2.30000 × 10^3^	6.67879 × 10^3^	2.30667 × 10^3^	2.30000 × 10^3^
	Rank	2	5	7	13	17	16	11	14	8	12	4	6	10	1	9	15	3
	Time	8.38	8.35	7.59	9.40	10.25	11.12	20.34	7.19	8.60	9.01	12.23	15.35	24.21	6.54	16.16	8.46	17.56
F9	Mean	3.04511 × 10^3^	3.04640 × 10^3^	3.02507 × 10^3^	3.06863 × 10^3^	4.23924 × 10^3^	3.95053 × 10^3^	3.15265 × 10^3^	3.02094 × 10^3^	3.39172 × 10^3^	3.12021 × 10^3^	3.16745 × 10^3^	2.99961 × 10^3^	3.06062 × 10^3^	3.07887 × 10^3^	3.76912 × 10^3^	3.18438 × 10^3^	3.10053 × 10^3^
	Std	3.11136 × 10^1^	3.35031 × 10^1^	2.37833 × 10^1^	3.74461 × 10^1^	1.10576 × 10^2^	1.63238 × 10^2^	8.60904 × 10^1^	3.13414 × 10^1^	1.58085 × 10^2^	4.93864 × 10^1^	1.27954 × 10^2^	2.65778 × 10^1^	4.96213 × 10^1^	5.70738 × 10^1^	9.91084 × 10^1^	3.60474 × 10^1^	5.38041 × 10^1^
	Worst	3.12629 × 10^3^	3.11301 × 10^3^	3.08736 × 10^3^	3.14490 × 10^3^	4.44248 × 10^3^	4.33105 × 10^3^	3.37668 × 10^3^	3.09692 × 10^3^	3.72875 × 10^3^	3.21388 × 10^3^	3.56791 × 10^3^	3.05612 × 10^3^	3.24095 × 10^3^	3.30886 × 10^3^	3.93160 × 10^3^	3.27722 × 10^3^	3.21935 × 10^3^
	Best	2.98817 × 10^3^	2.98076 × 10^3^	2.98236 × 10^3^	3.00406 × 10^3^	3.87108 × 10^3^	3.72735 × 10^3^	3.00872 × 10^3^	2.97323 × 10^3^	3.10887 × 10^3^	3.03046 × 10^3^	3.00086 × 10^3^	2.95198 × 10^3^	3.00972 × 10^3^	3.00970 × 10^3^	3.54578 × 10^3^	3.11984 × 10^3^	3.02342 × 10^3^
	Rank	4	5	3	7	17	16	11	2	14	10	12	1	6	8	15	13	9
	Time	9.03	8.97	7.90	9.70	10.78	11.25	20.11	7.89	9.04	8.27	12.45	14.41	22.50	7.06	15.16	9.20	19.12
F10	Mean	3.02756 × 10^3^	3.07349 × 10^3^	3.05622 × 10^3^	3.14236 × 10^3^	1.23788 × 10^4^	4.29116 × 10^3^	3.04666 × 10^3^	3.06579 × 10^3^	3.02869 × 10^3^	3.03705 × 10^3^	3.03951 × 10^3^	3.03827 × 10^3^	3.03436 × 10^3^	3.05960 × 10^3^	3.03541 × 10^3^	3.05615 × 10^3^	3.03106 × 10^3^
	Std	4.24816 × 10^1^	2.93074 × 10^1^	4.25644 × 10^1^	4.11161 × 10^1^	7.88180 × 10^2^	2.99096 × 10^2^	4.39229 × 10^1^	3.61294 × 10^1^	3.50208 × 10^1^	4.64331 × 10^1^	2.92204 × 10^1^	3.70343 × 10^1^	2.68539 × 10^1^	3.60872 × 10^1^	3.54486 × 10^1^	4.25255 × 10^1^	2.30192 × 10^1^
	Worst	3.09351 × 10^3^	3.11114 × 10^3^	3.10806 × 10^3^	3.27606 × 10^3^	1.35178 × 10^4^	5.10675 × 10^3^	3.11537 × 10^3^	3.11534 × 10^3^	3.08231 × 10^3^	3.10547 × 10^3^	3.08673 × 10^3^	3.09678 × 10^3^	3.07329 × 10^3^	3.11220 × 10^3^	3.08697 × 10^3^	3.11713 × 10^3^	3.08846 × 10^3^
	Best	2.96044 × 10^3^	2.96144 × 10^3^	2.96098 × 10^3^	3.07137 × 10^3^	1.01600 × 10^4^	3.75048 × 10^3^	2.96065 × 10^3^	2.96841 × 10^3^	2.96303 × 10^3^	2.96008 × 10^3^	2.95865 × 10^3^	2.96204 × 10^3^	2.98159 × 10^3^	2.95947 × 10^3^	2.96101 × 10^3^	2.96097 × 10^3^	2.97972 × 10^3^
	Rank	**1**	14	11	15	17	16	9	13	2	6	8	7	4	12	5	10	3
	Time	7.92	7.86	6.69	8.32	9.29	9.98	17.43	6.26	8.04	7.87	10.58	13.94	20.74	5.69	14.71	7.21	16.50
Mean Rank		**2.5**	5.4	7.80	10.5	15.3	15	8.60	9.7	8.9	10.70	8	4.70	9.30	8.4	11.4	11	5.2

**Table 11:** Results of Friedman test.

**Fun**	**SPNOA**	**NOA**	**APO**	**AROA**	**BWO**	**HEOA**	**RBMO**	**KOA**	**HFPSO**	**EAPSO**	**ACEPSO**	**RNEGDE**	**MCDE**	**HMRFO**	**IMODE**	**AGSK**	**LSHADE_** **cnEpSin**
F1	5.25000	5.25000	7.75000	12.76667	17.00000	16.00000	5.25000	5.25000	14.46667	13.23333	11.83333	5.25000	5.25000	12.70000	5.25000	5.25000	5.25000
F2	2.36667	5.10000	4.70000	10.66667	16.53333	16.30000	10.23333	10.50000	12.46667	12.76667	11.40000	6.36667	7.43333	11.00000	2.96667	8.46667	3.73333
F3	4.43333	8.90000	1.80000	9.63333	16.26667	16.73333	7.06667	10.16667	10.90000	13.93333	4.73333	5.30000	10.46667	11.43333	7.03333	10.13333	4.06667
F4	3.56667	3.56667	9.00000	10.96667	3.56667	3.76667	13.66667	14.83333	14.06667	15.80000	13.33333	8.90000	10.56667	3.56667	3.56667	12.53333	7.73333
F5	1.95000	2.70000	6.66667	5.90000	16.23333	16.70000	8.26667	6.73333	14.33333	14.23333	12.73333	9.33333	4.21667	12.46667	5.23333	4.83333	10.46667
F6	2.36667	2.46667	5.93333	8.83333	15.83333	15.63333	9.43333	6.50000	14.06667	12.20000	14.13333	8.20000	5.93333	10.46667	4.43333	7.53333	9.03333
F7	2.33333	2.73333	4.40000	6.90000	16.63333	16.36667	9.66667	6.63333	13.70000	12.83333	14.03333	7.46667	6.53333	12.26667	4.33333	5.43333	10.73333
F8	6.43333	4.53333	6.66667	11.40000	16.73333	12.60000	9.43333	8.90000	14.80000	12.20000	7.36667	5.76667	9.81667	12.20000	2.93333	3.33333	7.88333
F9	4.73333	6.33333	9.71667	10.10000	8.70000	13.40000	12.76667	7.61667	15.11667	14.46667	6.56667	9.23333	11.15000	8.08333	2.81667	2.90000	9.30000
F10	2.20000	5.91667	10.01667	9.60000	17.00000	12.13333	8.93333	8.38333	11.70000	11.13333	8.11667	10.58333	6.08333	12.11667	3.98333	4.73333	10.36667
Mean rank	3.56333	4.75000	6.66500	9.67667	14.45000	13.96333	9.47167	8.55167	13.56167	13.28000	10.42500	7.64000	7.74500	10.63000	4.25500	6.5150	7.8567
Final rank	1	3	5	11	17	16	10	9	15	14	12	6	7	13	2	4	8

**Table 12:** Results of Wilcoxon rank-sum test.

Fun	NOA	APO	AROA	BWO	HEOA	RBMO	KOA	HFPSO	EAPSO	ACEPSO	RNEGDE	MCDE	HMRFO	IMODE	AGSK	LSHADE_ cnEpSin
F1	NaN	1.27112 × 10^−5^	1.21079 × 10^−12^	1.21079 × 10^−12^	1.21079 × 10^−12^	NaN	NaN	9.43159 × 10^−13^	1.20882 × 10^−12^	1.21178 × 10^−12^	NaN	NaN	1.21178 × 10^−12^	NaN	NaN	NaN
	=	+	+	+	+	=	=	+	+	+	=	=	+	=	=	=
F2	1.02773 × 10^−6^	1.16711 × 10^−5^	9.91273 × 10^−11^	3.01797 × 10^−11^	3.01797 × 10^−11^	1.77588 × 10^−10^	3.01797 × 10^−11^	3.33631 × 10^−11^	5.49070 × 10^−11^	2.37147 × 10^−10^	5.18568 × 10^−7^	2.19474 × 10^−8^	6.71466 × 10^−10^	3.14656 × 10^−2^	3.33839 × 10^−11^	1.51780 × 10^−3^
	+	+	+	+	+	+	+	+	+	+	+	+	+	+	+	+
F3	1.16744 × 10^−5^	3.15647 × 10^−5^	2.83020 × 10^−8^	3.01797 × 10^−11^	3.01797 × 10^−11^	3.03354 × 10^−3^	3.96305 × 10^−8^	7.11859 × 10^−9^	1.32885 × 10^−10^	7.84460 × 10^−1^	2.39850 × 10^−1^	5.59991 × 10^−7^	9.06321 × 10^−8^	1.23618 × 10^−3^	6.52774 × 10^−8^	5.49327 × 10^−1^
	+	-	+	+	+	+	+	+	+	=	=	+	+	+	+	=
F4	NaN	1.21178 × 10^−12^	1.94574 × 10^−9^	NaN	1.60802 × 10^−1^	1.21178 × 10^−12^	1.21178 × 10^−12^	1.21178 × 10^−12^	1.21178 × 10^−12^	1.21178 × 10^−12^	1.21178 × 10^−12^	1.21178 × 10^−12^	NaN	NaN	1.21178 × 10^−12^	1.21178 × 10^−12^
	=	+	+	=	=	+	+	+	+	+	+	+	=	=	+	+
F5	4.71204 × 10^−4^	6.51201 × 10^−9^	1.20121 × 10^−8^	3.01608 × 10^−11^	3.01608 × 10^−11^	7.38018 × 10^−11^	4.61081 × 10^−10^	3.01608 × 10^−11^	3.01608 × 10^−11^	3.01608 × 10^−11^	8.14297 × 10^−11^	3.19605 × 10^−2^	3.01418 × 10^−11^	1.55665 × 10^−8^	2.01341 × 10^−8^	3.33214 × 10^−11^
	+	+	+	+	+	+	+	+	+	+	+	+	+	+	+	+
F6	4.30255 × 10^−2^	2.00164 × 10^−6^	1.61229 × 10^−10^	3.01797 × 10^−11^	3.01797 × 10^−11^	6.11770 × 10^−10^	1.47273 × 10^−7^	3.01986 × 10^−11^	1.09367 × 10^−10^	3.01986 × 10^−11^	8.48477 × 10^−9^	1.51780 × 10^−3^	1.32885 × 10^−10^	5.87373 × 10^−4^	4.61081 × 10^−10^	2.92155 × 10^−9^
	+	+	+	+	+	+	+	+	+	+	+	+	+	+	+	+
F7	1.91124 × 10^−2^	3.00589 × 10^−4^	7.11859 × 10^−9^	3.01986 × 10^−11^	3.01986 × 10^−11^	3.01986 × 10^−11^	8.48477 × 10^−9^	3.01797 × 10^−11^	3.01797 × 10^−11^	3.01986 × 10^−11^	5.53286 × 10^−8^	3.52006 × 10^−7^	3.01986 × 10^−11^	1.16744 × 10^−5^	2.78287 × 10^−7^	1.20567 × 10^−10^
	+	+	+	+	+	+	+	+	+	+	+	+	+	+	+	+
F8	3.56663 × 10^−2^	9.16423 × 10^−1^	1.05452 × 10^−7^	2.96727 × 10^−11^	7.60963 × 10^−5^	1.61659 × 10^−4^	5.67495 × 10^−3^	1.74755 × 10^−9^	2.26070 × 10^−8^	3.46592 × 10^−1^	5.03937 × 10^−2^	1.56255 × 10^−3^	2.07307 × 10^−8^	6.31437 × 10^−5^	1.81589 × 10^−5^	3.92372 × 10^−2^
	-	=	+	+	+	+	+	+	+	=	=	+	+	-	+	+
F9	2.12163 × 10^−2^	1.27860 × 10^−6^	3.03247 × 10^−7^	2.66259 × 10^−6^	5.83140 × 10^−9^	3.77900 × 10^−10^	2.40844 × 10^−3^	3.26169 × 10^−10^	6.13717 × 10^−11^	4.34056 × 10^−2^	2.47547 × 10^−6^	1.70516 × 10^−7^	2.37556 × 10^−3^	4.87420 × 10^−1^	5.58431 × 10^−3^	5.33782 × 10^−6^
	+	+	+	+	+	+	+	+	+	+	+	+	+	=	+	+
F10	2.64520 × 10^−4^	3.37036 × 10^−11^	1.78883 × 10^−11^	3.16021 × 10^−12^	7.75912 × 10^−11^	4.85151 × 10^−11^	5.59475 × 10^−9^	3.93193 × 10^−12^	1.05485 × 10^−10^	2.86431 × 10^−9^	7.57013 × 10^−12^	5.25518 × 10^−6^	7.56460 × 10^−12^	2.19273 × 10^−8^	3.08579 × 10^−7^	2.73277 × 10^−11^
	+	+	+	+	+	+	+	+	+	+	+	+	+	+	-	+
+/=/-	7/2/1	8/1/1	10/0/0	9/1/0	9/1/0	9/1/0	9/1/0	10/0/0	10/0/0	8/2/0	7/3/0	9/1/0	9/1/0	6/3/1	8/1/1	8/2/0

**Table 13:** Experimental data for the engineering problems.

Fun	Index	SPNOA	NOA	APO	AROA	BWO	HEOA	RBMO	KOA	HFPSO	EAPSO	ACEPSO	RNEGDE	HMRFO	IMODE	AGSK	LSHADE_ cnEpSin
RW1	Mean	4.11451 × 10^2^	4.19956 × 10^2^	6.42914 × 10^2^	1.88429 × 10^5^	9.05443 × 10^9^	1.54235 × 10^10^	6.04920 × 10^3^	3.23380 × 10^4^	4.99914 × 10^10^	5.72211 × 10^2^	7.67195 × 10^5^	1.70835 × 10^3^	3.75531 × 10^6^	1.13514 × 10^4^	8.77135 × 10^4^	6.01377 × 10^2^
	Std	1.91852 × 10^2^	9.05284 × 10^1^	5.23473 × 10^2^	6.86496 × 10^5^	5.74852 × 10^9^	6.50922 × 10^9^	9.61099 × 10^3^	7.29329 × 10^4^	1.39021 × 10^10^	1.92551 × 10^2^	4.83981 × 10^5^	3.95827 × 10^3^	1.42647 × 10^7^	2.52313 × 10^4^	2.26686 × 10^5^	2.35010 × 10^2^
	Worst	9.42743 × 10^2^	5.93197 × 10^2^	2.54802 × 10^3^	2.80771 × 10^6^	2.45759 × 10^10^	2.99552 × 10^10^	4.27600 × 10^4^	2.90340 × 10^5^	7.59000 × 10^10^	1.03432 × 10^3^	1.81599 × 10^6^	2.20787 × 10^4^	6.10622 × 10^7^	1.15048 × 10^5^	1.17325 × 10^6^	1.06895 × 10^3^
	Best	1.96279 × 10^2^	2.31540 × 10^2^	2.10879 × 10^2^	1.99653 × 10^2^	6.18321 × 10^8^	1.73210 × 10^9^	1.89474 × 10^2^	2.06342 × 10^2^	2.39095 × 10^10^	2.76884 × 10^2^	3.94758 × 10^2^	3.37579 × 10^2^	3.41016 × 10^2^	4.75425 × 10^2^	1.89389 × 10^2^	2.01015 × 10^2^
	Rank	1	2	5	11	14	15	7	9	16	3	12	6	13	8	10	4
RW2	Mean	7.04904 × 10^3^	7.04904 × 10^3^	7.04904 × 10^3^	1.10248 × 10^10^	7.38647 × 10^9^	2.53811 × 10^9^	2.39862 × 10^8^	7.04904 × 10^3^	1.05103 × 10^10^	7.04904 × 10^3^	1.56761 × 10^6^	7.04904 × 10^3^	7.04904 × 10^3^	9.76178 × 10^4^	7.04904 × 10^3^	7.04904 × 10^3^
	Std	1.03906 × 10^−10^	8.50073 × 10^−7^	4.24985 × 10^−7^	5.08825 × 10^9^	1.22171 × 10^9^	1.51561 × 10^9^	3.78414 × 10^8^	9.37291 × 10^−7^	1.47216 × 10^9^	3.61961 × 10^−6^	1.24711 × 10^6^	1.06293 × 10^−6^	1.05999 × 10^−3^	6.35570 × 10^4^	5.90752 × 10^−7^	8.51772 × 10^−6^
	Worst	7.04904 × 10^3^	7.04904 × 10^3^	7.04904 × 10^3^	1.36210 × 10^10^	8.97123 × 10^9^	5.64120 × 10^9^	1.31043 × 10^9^	7.04904 × 10^3^	1.27569 × 10^10^	7.04904 × 10^3^	4.38904 × 10^6^	7.04904 × 10^3^	7.04904 × 10^3^	2.94740 × 10^5^	7.04904 × 10^3^	7.04904 × 10^3^
	Best	7.04904 × 10^3^	7.04904 × 10^3^	7.04904 × 10^3^	1.14486 × 10^8^	4.29173 × 10^9^	4.43438 × 10^8^	7.18218 × 10^3^	7.04904 × 10^3^	5.83270 × 10^9^	7.04904 × 10^3^	7.04916 × 10^3^	7.04904 × 10^3^	7.04904 × 10^3^	3.30033 × 10^4^	7.04904 × 10^3^	7.04904 × 10^3^
	Rank	1	3	2	16	14	13	12	6	15	7	11	5	9	10	4	8
RW3	Mean	-4.49637 × 10^3^	-4.36452 × 10^3^	-4.52881 × 10^3^	-4.38290 × 10^3^	2.15865 × 10^6^	1.00705 × 10^6^	-3.62942 × 10^3^	-4.52912 × 10^3^	2.97416 × 10^8^	2.09280 × 10^3^	8.54403 × 10^5^	-1.61193 × 10^3^	4.46122 × 10^2^	6.23344 × 10^6^	-4.60535 × 10^2^	1.71578 × 10^1^
	Std	1.77968 × 10^2^	8.02571 × 10^2^	1.69693 × 10^0^	8.00878 × 10^2^	2.52781 × 10^5^	1.49415 × 10^6^	1.83086 × 10^3^	9.12312 × 10^−9^	2.83176 × 10^8^	1.33776 × 10^4^	2.21461 × 10^6^	2.21958 × 10^3^	1.22449 × 10^3^	1.55519 × 10^7^	1.15762 × 10^3^	1.56588 × 10^2^
	Worst	-3.55412 × 10^3^	-1.42719 × 10^2^	-4.51983 × 10^3^	-1.42528 × 10^2^	2.66147 × 10^6^	6.12588 × 10^6^	9.15754 × 10^1^	-4.52912 × 10^3^	8.85031 × 10^8^	7.07712 × 10^4^	6.41183 × 10^6^	5.66717 × 10^2^	6.84169 × 10^3^	8.60405 × 10^7^	1.07358 × 10^2^	2.66919 × 10^2^
	Best	-4.52912 × 10^3^	-4.52912 × 10^3^	-4.52912 × 10^3^	-4.52912 × 10^3^	1.89665 × 10^6^	-1.67689 × 10^3^	-4.52912 × 10^3^	-4.52912 × 10^3^	4.47452 × 10^6^	-4.52802 × 10^3^	-4.49801 × 10^3^	-4.52912 × 10^3^	-1.31326 × 10^2^	-1.57950 × 10^3^	-4.30151 × 10^3^	-1.42719 × 10^2^
	Rank	3	5	2	4	14	13	6	1	16	11	12	7	10	15	8	9
RW4	Mean	2.00000 × 10^0^	2.00000 × 10^0^	2.00000 × 10^0^	2.00000 × 10^0^	2.00004 × 10^0^	2.00000 × 10^0^	2.00000 × 10^0^	2.00000 × 10^0^	2.02879 × 10^0^	2.00000 × 10^0^	2.00000 × 10^0^	2.00000 × 10^0^	2.00000 × 10^0^	2.00000 × 10^0^	2.00000 × 10^0^	2.00000 × 10^0^
	Std	0.00000 × 10^0^	0.00000 × 10^0^	7.14170 × 10^−17^	0.00000 × 10^0^	5.24774 × 10^−5^	1.46101 × 10^−6^	2.01998 × 10^−16^	0.00000 × 10^0^	3.00488 × 10^−2^	1.84398 × 10^−16^	1.79729 × 10^−16^	1.84398 × 10^−16^	0.00000 × 10^0^	0.00000 × 10^0^	0.00000 × 10^0^	0.00000 × 10^0^
	Worst	2.00000 × 10^0^	2.00000 × 10^0^	2.00000 × 10^0^	2.00000 × 10^0^	2.00018 × 10^0^	2.00001 × 10^0^	2.00000 × 10^0^	2.00000 × 10^0^	2.11606 × 10^0^	2.00000 × 10^0^	2.00000 × 10^0^	2.00000 × 10^0^	2.00000 × 10^0^	2.00000 × 10^0^	2.00000 × 10^0^	2.00000 × 10^0^
	Best	2.00000 × 10^0^	2.00000 × 10^0^	2.00000 × 10^0^	2.00000 × 10^0^	2.00000 × 10^0^	2.00000 × 10^0^	2.00000 × 10^0^	2.00000 × 10^0^	2.00023 × 10^0^	2.00000 × 10^0^	2.00000 × 10^0^	2.00000 × 10^0^	2.00000 × 10^0^	2.00000 × 10^0^	2.00000 × 10^0^	2.00000 × 10^0^
	Rank	1	1	9	1	15	14	13	1	16	11	10	12	1	1	1	1
RW5	Mean	2.58143 × 10^0^	2.57222 × 10^0^	2.72959 × 10^0^	2.59551 × 10^0^	3.15290 × 10^0^	2.73169 × 10^0^	2.65149 × 10^0^	2.61476 × 10^0^	5.54300 × 10^2^	2.73493 × 10^0^	3.22476 × 10^0^	2.63034 × 10^0^	2.62618 × 10^0^	2.56867 × 10^0^	2.56916 × 10^0^	2.63792 × 10^0^
	Std	3.18016 × 10^−2^	1.66306 × 10^−2^	2.44981 × 10^−1^	4.15688 × 10^−2^	3.97667 × 10^−1^	1.74757 × 10^−1^	2.52427 × 10^−1^	4.09099 × 10^−2^	4.53986 × 10^2^	1.92494 × 10^−1^	2.80472 × 10^−1^	5.09117 × 10^−2^	4.60566 × 10^−2^	1.64067 × 10^−2^	1.89406 × 10^−2^	6.23981 × 10^−2^
	Worst	2.70832 × 10^0^	2.62405 × 10^0^	3.38750 × 10^0^	2.71988 × 10^0^	4.55603 × 10^0^	3.45412 × 10^0^	3.38632 × 10^0^	2.70978 × 10^0^	1.60448 × 10^3^	3.48699 × 10^0^	3.38643 × 10^0^	2.73033 × 10^0^	2.75863 × 10^0^	2.62163 × 10^0^	2.63393 × 10^0^	2.80662 × 10^0^
	Best	2.55782 × 10^0^	2.55782 × 10^0^	2.56034 × 10^0^	2.55985 × 10^0^	2.68921 × 10^0^	2.55973 × 10^0^	2.55782 × 10^0^	2.55785 × 10^0^	3.56486 × 10^1^	2.56432 × 10^0^	2.58710 × 10^0^	2.55836 × 10^0^	2.57260 × 10^0^	2.55782 × 10^0^	2.55782 × 10^0^	2.55782 × 10^0^
	Rank	4	3	11	5	14	12	10	6	16	13	15	8	7	1	2	9
RW6	Mean	1.08232 × 10^0^	1.07654 × 10^0^	1.08817 × 10^0^	1.07654 × 10^0^	1.23602 × 10^0^	1.24617 × 10^0^	1.14014 × 10^0^	1.11702 × 10^0^	1.23687 × 10^0^	1.08232 × 10^0^	1.23599 × 10^0^	1.13205 × 10^0^	1.07654 × 10^0^	1.09989 × 10^0^	1.07654 × 10^0^	1.10545 × 10^0^
	Std	3.16688 × 10^−2^	4.51681 × 10^−16^	2.57636 × 10^−2^	4.51681 × 10^−16^	4.32751 × 10^−2^	2.09649 × 10^−2^	8.50169 × 10^−2^	7.46182 × 10^−2^	6.22890 × 10^−2^	3.16688 × 10^−2^	5.70455 × 10^−2^	7.87957 × 10^−2^	4.51681 × 10^−16^	5.98958 × 10^−2^	4.51681 × 10^−16^	6.57487 × 10^−2^
	Worst	1.25000 × 10^0^	1.07654 × 10^0^	1.18338 × 10^0^	1.07654 × 10^0^	1.25000 × 10^0^	1.25000 × 10^0^	1.25000 × 10^0^	1.25000 × 10^0^	1.35000 × 10^0^	1.25000 × 10^0^	1.35000 × 10^0^	1.25000 × 10^0^	1.07654 × 10^0^	1.25000 × 10^0^	1.07654 × 10^0^	1.25000 × 10^0^
	Best	1.07654 × 10^0^	1.07654 × 10^0^	1.07654 × 10^0^	1.07654 × 10^0^	1.08039 × 10^0^	1.13517 × 10^0^	1.07654 × 10^0^	1.07654 × 10^0^	1.13450 × 10^0^	1.07654 × 10^0^	1.07654 × 10^0^	1.07654 × 10^0^	1.07654 × 10^0^	1.07654 × 10^0^	1.07654 × 10^0^	1.07654 × 10^0^
	Rank	5	1	7	2	14	16	12	10	15	6	13	11	3	8	4	9
RW7	Mean	2.92557 × 10^0^	2.92631 × 10^0^	3.45225 × 10^0^	4.50929 × 10^0^	4.03721 × 10^0^	4.24369 × 10^0^	3.25966 × 10^0^	2.93005 × 10^0^	1.02052 × 10^19^	3.01469 × 10^0^	6.90225 × 10^0^	2.99434 × 10^0^	3.06321 × 10^0^	2.92483 × 10^0^	2.92483 × 10^0^	3.62780 × 10^0^
	Std	4.04047 × 10^−3^	5.61470 × 10^−3^	7.55071 × 10^−1^	1.45141 × 10^0^	3.56246 × 10^−1^	1.80297 × 10^−1^	4.75930 × 10^−1^	9.49248 × 10^−3^	4.12741 × 10^19^	2.33472 × 10^−1^	1.73236 × 10^0^	1.98976 × 10^−1^	3.94017 × 10^−1^	4.51681 × 10^−16^	4.51681 × 10^−16^	6.75701 × 10^−1^
	Worst	2.94696 × 10^0^	2.94696 × 10^0^	5.39078 × 10^0^	1.07837 × 10^1^	4.95377 × 10^0^	5.05007 × 10^0^	4.20646 × 10^0^	2.94696 × 10^0^	2.19519 × 10^20^	4.20694 × 10^0^	1.33069 × 10^1^	4.00423 × 10^0^	4.69000 × 10^0^	2.92483 × 10^0^	2.92483 × 10^0^	4.76750 × 10^0^
	Best	2.92483 × 10^0^	2.92483 × 10^0^	2.92483 × 10^0^	2.95057 × 10^0^	3.32132 × 10^0^	3.82394 × 10^0^	2.92483 × 10^0^	2.92483 × 10^0^	1.43360 × 10^1^	2.92485 × 10^0^	3.93958 × 10^0^	2.92483 × 10^0^	2.92483 × 10^0^	2.92483 × 10^0^	2.92483 × 10^0^	2.92483 × 10^0^
	Rank	3	4	10	14	12	13	9	5	16	7	15	6	8	2	1	11
RW8	Mean	2.68874 × 10^4^	2.68874 × 10^4^	2.68874 × 10^4^	2.68874 × 10^4^	2.68878 × 10^4^	2.69155 × 10^4^	2.68874 × 10^4^	2.68874 × 10^4^	3.41978 × 10^4^	2.68874 × 10^4^	2.68874 × 10^4^	2.68874 × 10^4^	2.68874 × 10^4^	2.68874 × 10^4^	2.68874 × 10^4^	2.68874 × 10^4^
	Std	1.48007 × 10^−11^	1.48007 × 10^−11^	1.48007 × 10^−11^	1.48007 × 10^−11^	5.56508 × 10^−1^	4.82133 × 10^1^	1.48007 × 10^−11^	1.48007 × 10^−11^	5.17825 × 10^3^	1.48007 × 10^−11^	1.48007 × 10^−11^	1.48007 × 10^−11^	1.55231 × 10^−11^	1.48007 × 10^−11^	1.48007 × 10^−11^	1.48007 × 10^−11^
	Worst	2.68874 × 10^4^	2.68874 × 10^4^	2.68874 × 10^4^	2.68874 × 10^4^	2.68895 × 10^4^	2.71327 × 10^4^	2.68874 × 10^4^	2.68874 × 10^4^	5.12950 × 10^4^	2.68874 × 10^4^	2.68874 × 10^4^	2.68874 × 10^4^	2.68874 × 10^4^	2.68874 × 10^4^	2.68874 × 10^4^	2.68874 × 10^4^
	Best	2.68874 × 10^4^	2.68874 × 10^4^	2.68874 × 10^4^	2.68874 × 10^4^	2.68874 × 10^4^	2.68874 × 10^4^	2.68874 × 10^4^	2.68874 × 10^4^	2.90868 × 10^4^	2.68874 × 10^4^	2.68874 × 10^4^	2.68874 × 10^4^	2.68874 × 10^4^	2.68874 × 10^4^	2.68874 × 10^4^	2.68874 × 10^4^
	Rank	1	1	1	1	14	15	1	1	16	1	1	1	1	1	1	1
RW9	Mean	5.44690 × 10^4^	5.63260 × 10^4^	5.50522 × 10^4^	5.47953 × 10^4^	2.92426 × 10^5^	9.30380 × 10^4^	5.65238 × 10^4^	5.58548 × 10^4^	2.23610 × 10^5^	5.79753 × 10^4^	5.81133 × 10^4^	5.75237 × 10^4^	5.75526 × 10^4^	1.21775 × 10^5^	5.36389 × 10^4^	5.76247 × 10^4^
	Std	1.89137 × 10^3^	2.56572 × 10^3^	2.38930 × 10^3^	2.13430 × 10^3^	2.95989 × 10^5^	1.36386 × 10^4^	3.61414 × 10^3^	2.58746 × 10^3^	2.54227 × 10^5^	2.76756 × 10^3^	5.76226 × 10^3^	2.19780 × 10^3^	2.08495 × 10^3^	2.60313 × 10^4^	1.48080 × 10^−4^	2.03603 × 10^3^
	Worst	5.91851 × 10^4^	5.91852 × 10^4^	5.92358 × 10^4^	5.91856 × 10^4^	1.54993 × 10^6^	1.28228 × 10^5^	6.65224 × 10^4^	5.91851 × 10^4^	1.55970 × 10^6^	6.57243 × 10^4^	7.08536 × 10^4^	5.91853 × 10^4^	5.96786 × 10^4^	1.71656 × 10^5^	5.36389 × 10^4^	5.91857 × 10^4^
	Best	5.36389 × 10^4^	5.36389 × 10^4^	5.36389 × 10^4^	5.36389 × 10^4^	9.69592 × 10^4^	7.06600 × 10^4^	5.36389 × 10^4^	5.36389 × 10^4^	1.11982 × 10^5^	5.36417 × 10^4^	5.36390 × 10^4^	5.36389 × 10^4^	5.36392 × 10^4^	6.69352 × 10^4^	5.36389 × 10^4^	5.36389 × 10^4^
	Rank	2	6	4	3	16	13	7	5	15	11	12	8	9	14	1	10
RW10	Mean	2.99442 × 10^3^	2.99442 × 10^3^	2.99442 × 10^3^	2.99442 × 10^3^	3.05542 × 10^3^	4.14071 × 10^3^	2.99442 × 10^3^	2.99442 × 10^3^	7.35079 × 10^3^	2.99442 × 10^3^	2.99442 × 10^3^	2.99442 × 10^3^	2.99442 × 10^3^	2.99442 × 10^3^	2.99442 × 10^3^	2.99442 × 10^3^
	Std	0.00000 × 10^0^	0.00000 × 10^0^	0.00000 × 10^0^	2.02970 × 10^−9^	1.81110 × 10^1^	1.21038 × 10^3^	0.00000 × 10^0^	0.00000 × 10^0^	2.00337 × 10^3^	4.13692 × 10^−13^	4.75261 × 10^−7^	0.00000 × 10^0^	8.09634 × 10^−7^	1.63989 × 10^−8^	0.00000 × 10^0^	8.44445 × 10^−14^
	Worst	2.99442 × 10^3^	2.99442 × 10^3^	2.99442 × 10^3^	2.99442 × 10^3^	3.08379 × 10^3^	8.74655 × 10^3^	2.99442 × 10^3^	2.99442 × 10^3^	1.20851 × 10^4^	2.99442 × 10^3^	2.99442 × 10^3^	2.99442 × 10^3^	2.99442 × 10^3^	2.99442 × 10^3^	2.99442 × 10^3^	2.99442 × 10^3^
	Best	2.99442 × 10^3^	2.99442 × 10^3^	2.99442 × 10^3^	2.99442 × 10^3^	3.01155 × 10^3^	3.06584 × 10^3^	10+A71:P73	2.99442 × 10^3^	4.63365 × 10^3^	2.99442 × 10^3^	2.99442 × 10^3^	2.99442 × 10^3^	2.99442 × 10^3^	2.99442 × 10^3^	2.99442 × 10^3^	2.99442 × 10^3^
	Rank	1	1	1	10	14	15	1	1	16	9	12	1	13	11	1	8
RW11	Mean	3.22130 × 10^−2^	3.22130 × 10^−2^	2.90717 × 10^3^	4.19929 × 10^3^	1.56312 × 10^4^	1.73668 × 10^4^	3.87621 × 10^3^	3.23047 × 10^2^	1.99843 × 10^6^	3.55321 × 10^3^	3.87621 × 10^3^	1.93813 × 10^3^	5.81692 × 10^−2^	3.22130 × 10^−2^	3.22130 × 10^−2^	1.29209 × 10^3^
	Std	2.80359 × 10^−13^	5.15337 × 10^−11^	4.51663 × 10^3^	4.88399 × 10^3^	1.55963 × 10^4^	1.41619 × 10^4^	4.82849 × 10^3^	1.76923 × 10^3^	1.06889 × 10^6^	4.74959 × 10^3^	4.82849 × 10^3^	3.94244 × 10^3^	1.67468 × 10^−2^	2.11725 × 10^−17^	5.54333 × 10^−12^	3.35043 × 10^3^
	Worst	3.22130 × 10^−2^	3.22130 × 10^−2^	9.69048 × 10^3^	9.69048 × 10^3^	5.95195 × 10^4^	5.96466 × 10^4^	9.69048 × 10^3^	9.69048 × 10^3^	4.76148 × 10^6^	9.69048 × 10^3^	9.69048 × 10^3^	9.69048 × 10^3^	1.20911 × 10^−1^	3.22130 × 10^−2^	3.22130 × 10^−2^	9.69048 × 10^3^
	Best	3.22130 × 10^−2^	3.22130 × 10^−2^	3.22130 × 10^−2^	3.22130 × 10^−2^	1.97276 × 10^1^	9.70095 × 10^2^	3.22130 × 10^−2^	3.22130 × 10^−2^	4.65934 × 10^5^	3.30542 × 10^−2^	3.22130 × 10^−2^	3.22130 × 10^−2^	3.58056 × 10^−2^	3.22130 × 10^−2^	3.22130 × 10^−2^	3.22130 × 10^−2^
	Rank	2	4	9	13	14	15	11	6	16	10	12	8	5	1	3	7
RW12	Mean	1.26652 × 10^−2^	1.26652 × 10^−2^	1.26653 × 10^−2^	1.26653 × 10^−2^	1.31947 × 10^−2^	1.86672 × 10^−2^	1.26652 × 10^−2^	1.26652 × 10^−2^	3.66197 × 10^3^	1.32394 × 10^−2^	1.27351 × 10^−2^	1.26874 × 10^−2^	1.26889 × 10^−2^	1.26652 × 10^−2^	1.26652 × 10^−2^	1.26998 × 10^−2^
	Std	2.64693 × 10^−9^	2.47923 × 10^−9^	6.03514 × 10^−8^	7.75367 × 10^−8^	6.57864 × 10^−4^	1.70536 × 10^−3^	5.95042 × 10^−10^	1.23082 × 10^−8^	3.50014 × 10^3^	9.73149 × 10^−4^	8.53476 × 10^−5^	5.81811 × 10^−5^	1.68253 × 10^−5^	3.48252 × 10^−8^	3.56431 × 10^−11^	6.18913 × 10^−5^
	Worst	1.26652 × 10^−2^	1.26652 × 10^−2^	1.26655 × 10^−2^	1.26656 × 10^−2^	1.53007 × 10^−2^	2.18388 × 10^−2^	1.26652 × 10^−2^	1.26653 × 10^−2^	9.83855 × 10^3^	1.64396 × 10^−2^	1.31285 × 10^−2^	1.29842 × 10^−2^	1.27338 × 10^−2^	1.26654 × 10^−2^	1.26652 × 10^−2^	1.29079 × 10^−2^
	Best	1.26652 × 10^−2^	1.26652 × 10^−2^	1.26652 × 10^−2^	1.26652 × 10^−2^	1.27396 × 10^−2^	1.35433 × 10^−2^	1.26652 × 10^−2^	1.26652 × 10^−2^	4.91290 × 10^−2^	1.26654 × 10^−2^	1.26799 × 10^−2^	1.26652 × 10^−2^	1.26677 × 10^−2^	1.26652 × 10^−2^	1.26652 × 10^−2^	1.26652 × 10^−2^
	Rank	3	4	7	8	13	15	2	5	16	14	12	9	10	6	1	11
RW13	Mean	6.03345 × 10^3^	6.03629 × 10^3^	6.11963 × 10^3^	6.04481 × 10^3^	6.15012 × 10^3^	6.63283 × 10^3^	6.04160 × 10^3^	6.04354 × 10^3^	2.44561 × 10^4^	6.28402 × 10^3^	6.24758 × 10^3^	6.08476 × 10^3^	6.18985 × 10^3^	6.59473 × 10^3^	6.03798 × 10^3^	6.09407 × 10^3^
	Std	4.95980 × 10^0^	1.23413 × 10^1^	1.25585 × 10^2^	2.08673 × 10^1^	5.53465 × 10^1^	4.63523 × 10^2^	1.30251 × 10^1^	1.56682 × 10^1^	1.08286 × 10^4^	4.00251 × 10^2^	4.89824 × 10^2^	9.12061 × 10^1^	1.87640 × 10^2^	9.72729 × 10^2^	1.10521 × 10^1^	1.04231 × 10^2^
	Worst	6.05971 × 10^3^	6.09053 × 10^3^	6.41009 × 10^3^	6.09053 × 10^3^	6.27134 × 10^3^	7.71363 × 10^3^	6.05971 × 10^3^	6.09053 × 10^3^	5.80304 × 10^4^	7.33284 × 10^3^	8.65644 × 10^3^	6.41009 × 10^3^	6.82041 × 10^3^	1.08428 × 10^4^	6.05971 × 10^3^	6.41009 × 10^3^
	Best	6.03255 × 10^3^	6.03255 × 10^3^	6.03255 × 10^3^	6.03255 × 10^3^	6.05834 × 10^3^	6.09938 × 10^3^	6.03255 × 10^3^	6.03255 × 10^3^	1.13781 × 10^4^	6.03255 × 10^3^	6.03255 × 10^3^	6.03255 × 10^3^	6.03293 × 10^3^	6.06459 × 10^3^	6.03255 × 10^3^	6.03255 × 10^3^
	Rank	1	2	9	6	10	15	4	5	16	13	12	7	11	14	3	8
RW14	Mean	1.67022 × 10^0^	1.67022 × 10^0^	1.67022 × 10^0^	1.67022 × 10^0^	1.85170 × 10^0^	3.38587 × 10^0^	1.67022 × 10^0^	1.67022 × 10^0^	6.44316 × 10^0^	1.71003 × 10^0^	1.67294 × 10^0^	1.67022 × 10^0^	1.67022 × 10^0^	1.67022 × 10^0^	1.67022 × 10^0^	1.67022 × 10^0^
	Std	1.70007 × 10^−16^	2.10246 × 10^−16^	1.79729 × 10^−16^	1.06925 × 10^−12^	8.92151 × 10^−2^	6.81760 × 10^−1^	1.74935 × 10^−16^	2.25841 × 10^−16^	1.41549 × 10^0^	1.01174 × 10^−1^	1.46148 × 10^−2^	2.18183 × 10^−16^	1.28152 × 10^−13^	4.51681 × 10^−16^	2.25841 × 10^−16^	2.10246 × 10^−16^
	Worst	1.67022 × 10^0^	1.67022 × 10^0^	1.67022 × 10^0^	1.67022 × 10^0^	2.13688 × 10^0^	4.20932 × 10^0^	1.67022 × 10^0^	1.67022 × 10^0^	9.42532 × 10^0^	2.09517 × 10^0^	1.75031 × 10^0^	1.67022 × 10^0^	1.67022 × 10^0^	1.67022 × 10^0^	1.67022 × 10^0^	1.67022 × 10^0^
	Best	1.67022 × 10^0^	1.67022 × 10^0^	1.67022 × 10^0^	1.67022 × 10^0^	1.72047 × 10^0^	1.95262 × 10^0^	1.67022 × 10^0^	1.67022 × 10^0^	3.71722 × 10^0^	1.67022 × 10^0^	1.67022 × 10^0^	1.67022 × 10^0^	1.67022 × 10^0^	1.67022 × 10^0^	1.67022 × 10^0^	1.67022 × 10^0^
	Rank	1	5	3	10	14	15	2	8	16	13	12	6	9	11	7	4
RW15	Mean	2.63896 × 10^2^	2.63896 × 10^2^	2.63896 × 10^2^	2.63896 × 10^2^	2.64183 × 10^2^	2.63970 × 10^2^	2.63896 × 10^2^	2.63896 × 10^2^	2.70018 × 10^2^	2.63923 × 10^2^	2.64527 × 10^2^	2.63896 × 10^2^	2.63896 × 10^2^	2.63896 × 10^2^	2.63896 × 10^2^	2.63896 × 10^2^
	Std	1.05556 × 10^−14^	1.49278 × 10^−14^	0.00000 × 10^0^	0.00000 × 10^0^	2.43323 × 10^−1^	1.10130 × 10^−1^	9.26246 × 10^−14^	0.00000 × 10^0^	3.79399 × 10^0^	3.82059 × 10^−2^	3.45920 × 10^0^	0.00000 × 10^0^	1.21347 × 10^−5^	5.78152 × 10^−14^	0.00000 × 10^0^	0.00000 × 10^0^
	Worst	2.63896 × 10^2^	2.63896 × 10^2^	2.63896 × 10^2^	2.63896 × 10^2^	2.64810 × 10^2^	2.64419 × 10^2^	2.63896 × 10^2^	2.63896 × 10^2^	2.78691 × 10^2^	2.64038 × 10^2^	2.82843 × 10^2^	2.63896 × 10^2^	2.63896 × 10^2^	2.63896 × 10^2^	2.63896 × 10^2^	2.63896 × 10^2^
	Best	2.63896 × 10^2^	2.63896 × 10^2^	2.63896 × 10^2^	2.63896 × 10^2^	2.63903 × 10^2^	2.63896 × 10^2^	2.63896 × 10^2^	2.63896 × 10^2^	2.64111 × 10^2^	2.63896 × 10^2^	2.63896 × 10^2^	2.63896 × 10^2^	2.63896 × 10^2^	2.63896 × 10^2^	2.63896 × 10^2^	2.63896 × 10^2^
	Rank	7	8	1	1	14	13	9	1	16	12	15	1	11	10	1	1
RW16	Mean	2.35242 × 10^−1^	2.35242 × 10^−1^	2.35242 × 10^−1^	2.35242 × 10^−1^	2.35371 × 10^−1^	2.38847 × 10^−1^	2.35242 × 10^−1^	2.35242 × 10^−1^	5.31656 × 10^−1^	2.35242 × 10^−1^	2.35242 × 10^−1^	2.35242 × 10^−1^	2.35242 × 10^−1^	2.36091 × 10^−1^	2.35242 × 10^−1^	2.35242 × 10^−1^
	Std	1.41150 × 10^−16^	1.41150 × 10^−16^	1.41150 × 10^−16^	1.41150 × 10^−16^	1.34124 × 10^−4^	1.74153 × 10^−2^	1.41150 × 10^−16^	1.41150 × 10^−16^	1.17212 × 10^−1^	1.41150 × 10^−16^	1.41150 × 10^−16^	1.41150 × 10^−16^	5.49757 × 10^−14^	4.64824 × 10^−3^	1.41150 × 10^−16^	1.41150 × 10^−16^
	Worst	2.35242 × 10^−1^	2.35242 × 10^−1^	2.35242 × 10^−1^	2.35242 × 10^−1^	2.35924 × 10^−1^	3.30810 × 10^−1^	2.35242 × 10^−1^	2.35242 × 10^−1^	7.92661 × 10^−1^	2.35242 × 10^−1^	2.35242 × 10^−1^	2.35242 × 10^−1^	2.35242 × 10^−1^	2.60702 × 10^−1^	2.35242 × 10^−1^	2.35242 × 10^−1^
	Best	2.35242 × 10^−1^	2.35242 × 10^−1^	2.35242 × 10^−1^	2.35242 × 10^−1^	2.35245 × 10^−1^	2.35242 × 10^−1^	2.35242 × 10^−1^	2.35242 × 10^−1^	3.15014 × 10^−1^	2.35242 × 10^−1^	2.35242 × 10^−1^	2.35242 × 10^−1^	2.35242 × 10^−1^	2.35242 × 10^−1^	2.35242 × 10^−1^	2.35242 × 10^−1^
	Rank	1	1	1	1	13	15	1	1	16	1	1	1	12	14	1	1
RW17	Mean	1.60903 × 10^1^	1.60903 × 10^1^	1.60904 × 10^1^	1.60903 × 10^1^	2.62312 × 10^1^	1.30526 × 10^2^	1.60903 × 10^1^	1.60903 × 10^1^	5.56956 × 10^2^	1.68104 × 10^1^	1.71200 × 10^1^	1.60903 × 10^1^	1.65958 × 10^1^	1.60903 × 10^1^	1.60903 × 10^1^	1.60903 × 10^1^
	Std	7.10543 × 10^−15^	3.71448 × 10^−13^	5.72761 × 10^−4^	3.36295 × 10^−11^	7.68225 × 10^0^	1.30093 × 10^2^	7.53092 × 10^−15^	4.39192 × 10^−13^	4.99358 × 10^2^	2.69181 × 10^−1^	2.96212 × 10^−2^	7.98185 × 10^−15^	1.96095 × 10^−1^	2.05893 × 10^−14^	3.48905 × 10^−14^	8.59722 × 10^−15^
	Worst	1.60903 × 10^1^	1.60903 × 10^1^	1.60933 × 10^1^	1.60903 × 10^1^	5.56504 × 10^1^	5.07668 × 10^2^	1.60903 × 10^1^	1.60903 × 10^1^	1.94460 × 10^3^	1.71341 × 10^1^	1.71364 × 10^1^	1.60903 × 10^1^	1.70310 × 10^1^	1.60903 × 10^1^	1.60903 × 10^1^	1.60903 × 10^1^
	Best	1.60903 × 10^1^	1.60903 × 10^1^	1.60903 × 10^1^	1.60903 × 10^1^	1.77153 × 10^1^	2.42457 × 10^1^	1.60903 × 10^1^	1.60903 × 10^1^	1.61936 × 10^2^	1.62008 × 10^1^	1.70178 × 10^1^	1.60903 × 10^1^	1.62315 × 10^1^	1.60903 × 10^1^	1.60903 × 10^1^	1.60903 × 10^1^
	Rank	1	7	10	9	14	15	2	8	16	12	13	3	11	5	6	4
RW18	Mean	2.54528 × 10^0^	2.54917 × 10^0^	2.77263 × 10^0^	2.64662 × 10^0^	5.10167 × 10^0^	7.47106 × 10^0^	2.54830 × 10^0^	2.55005 × 10^0^	4.78730 × 10^5^	3.60383 × 10^0^	2.96808 × 10^0^	2.59724 × 10^0^	2.91715 × 10^0^	5.79995 × 10^3^	2.54460 × 10^0^	2.55888 × 10^0^
	Std	8.16128 × 10^−3^	7.01528 × 10^−3^	1.79249 × 10^−1^	1.40098 × 10^−1^	3.27499 × 10^0^	1.74248 × 10^0^	1.36312 × 10^−2^	1.43179 × 10^−2^	5.67207 × 10^5^	6.93797 × 10^−1^	2.23519 × 10^−1^	1.73504 × 10^−1^	1.42898 × 10^−1^	3.07831 × 10^4^	1.75888 × 10^−3^	4.07210 × 10^−2^
	Worst	2.58849 × 10^0^	2.57354 × 10^0^	3.07992 × 10^0^	3.11950 × 10^0^	2.22643 × 10^1^	9.97153 × 10^0^	2.58854 × 10^0^	2.59257 × 10^0^	2.71497 × 10^6^	5.18099 × 10^0^	3.12775 × 10^0^	3.41486 × 10^0^	3.35359 × 10^0^	1.68740 × 10^5^	2.55272 × 10^0^	2.74581 × 10^0^
	Best	2.54379 × 10^0^	2.54390 × 10^0^	2.54390 × 10^0^	2.54379 × 10^0^	4.19891 × 10^0^	4.58741 × 10^0^	2.54379 × 10^0^	2.54382 × 10^0^	3.72096 × 10^1^	2.67630 × 10^0^	2.54380 × 10^0^	2.54379 × 10^0^	2.65813 × 10^0^	4.68937 × 10^0^	2.54379 × 10^0^	2.54379 × 10^0^
	Rank	2	4	9	8	13	14	3	5	16	12	11	7	10	15	1	6
RW19	Mean	5.24451 × 10^2^	5.24451 × 10^2^	5.25624 × 10^2^	5.25374 × 10^2^	5.98400 × 10^2^	5.75252 × 10^2^	5.26084 × 10^2^	5.24672 × 10^2^	7.54060 × 10^2^	5.29968 × 10^2^	5.29533 × 10^2^	5.24931 × 10^2^	5.27233 × 10^2^	5.24588 × 10^2^	5.24473 × 10^2^	5.26230 × 10^2^
	Std	7.35201 × 10^−4^	2.62083 × 10^−4^	2.06204 × 10^0^	2.02529 × 10^0^	1.05851 × 10^1^	1.05023 × 10^1^	2.75423 × 10^0^	1.11881 × 10^0^	5.35250 × 10^1^	3.03496 × 10^0^	3.99180 × 10^0^	1.58823 × 10^0^	3.10737 × 10^0^	1.22109 × 10^−1^	1.74129 × 10^−2^	2.81335 × 10^0^
	Worst	5.24454 × 10^2^	5.24452 × 10^2^	5.32281 × 10^2^	5.31648 × 10^2^	6.20814 × 10^2^	6.04842 × 10^2^	5.30574 × 10^2^	5.30594 × 10^2^	8.59495 × 10^2^	5.34278 × 10^2^	5.44231 × 10^2^	5.30808 × 10^2^	5.32708 × 10^2^	5.24968 × 10^2^	5.24526 × 10^2^	5.31303 × 10^2^
	Best	5.24451 × 10^2^	5.24451 × 10^2^	5.24503 × 10^2^	5.24452 × 10^2^	5.77180 × 10^2^	5.56338 × 10^2^	5.24451 × 10^2^	5.24451 × 10^2^	6.19076 × 10^2^	5.25095 × 10^2^	5.24567 × 10^2^	5.24451 × 10^2^	5.24499 × 10^2^	5.24461 × 10^2^	5.24453 × 10^2^	5.24455 × 10^2^
	Rank	2	1	8	7	15	14	9	5	16	13	12	6	11	4	3	10
RW20	Mean	1.69582 × 10^4^	1.69582 × 10^4^	1.69582 × 10^4^	1.69582 × 10^4^	1.73555 × 10^4^	1.84883 × 10^4^	1.69582 × 10^4^	1.69582 × 10^4^	9.73181 × 10^4^	1.69582 × 10^4^	1.69589 × 10^4^	1.69582 × 10^4^	1.69582 × 10^4^	2.42136 × 10^4^	1.69582 × 10^4^	1.69582 × 10^4^
	Std	7.40034 × 10^−12^	7.40034 × 10^−12^	7.40034 × 10^−12^	7.40034 × 10^−12^	2.67061 × 10^2^	2.25625 × 10^3^	7.40034 × 10^−12^	7.40034 × 10^−12^	3.71691 × 10^4^	5.85048 × 10^−12^	3.65830 × 10^0^	7.40034 × 10^−12^	3.05391 × 10^−6^	4.45201 × 10^3^	7.40034 × 10^−12^	7.40034 × 10^−12^
	Worst	1.69582 × 10^4^	1.69582 × 10^4^	1.69582 × 10^4^	1.69582 × 10^4^	1.80791 × 10^4^	2.85797 × 10^4^	1.69582 × 10^4^	1.69582 × 10^4^	2.22504 × 10^5^	1.69582 × 10^4^	1.69782 × 10^4^	1.69582 × 10^4^	1.69582 × 10^4^	3.20648 × 10^4^	1.69582 × 10^4^	1.69582 × 10^4^
	Best	1.69582 × 10^4^	1.69582 × 10^4^	1.69582 × 10^4^	1.69582 × 10^4^	1.70720 × 10^4^	1.70588 × 10^4^	1.69582 × 10^4^	1.69582 × 10^4^	3.49772 × 10^4^	1.69582 × 10^4^	1.69582 × 10^4^	1.69582 × 10^4^	1.69582 × 10^4^	1.69582 × 10^4^	1.69582 × 10^4^	1.69582 × 10^4^
	Rank	1	1	1	1	13	14	1	1	16	10	12	1	11	15	1	1
RW21	Mean	2.65856 × 10^0^	2.65856 × 10^0^	2.67903 × 10^0^	2.65992 × 10^0^	3.43281 × 10^0^	3.20321 × 10^0^	2.79441 × 10^0^	2.66129 × 10^0^	1.28549 × 10^3^	2.75875 × 10^0^	2.65856 × 10^0^	2.67540 × 10^0^	2.68799 × 10^0^	2.65856 × 10^0^	2.65856 × 10^0^	2.67900 × 10^0^
	Std	4.51681 × 10^−16^	4.51681 × 10^−16^	2.08172 × 10^−2^	7.47359 × 10^−3^	2.91693 × 10^−1^	3.63380 × 10^−1^	3.32206 × 10^−1^	1.03854 × 10^−2^	4.48467 × 10^3^	1.56781 × 10^−1^	4.51681 × 10^−16^	4.72136 × 10^−2^	6.61790 × 10^−2^	4.51681 × 10^−16^	4.51681 × 10^−16^	4.63914 × 10^−2^
	Worst	2.65856 × 10^0^	2.65856 × 10^0^	2.69949 × 10^0^	2.69949 × 10^0^	3.64776 × 10^0^	3.95978 × 10^0^	3.62625 × 10^0^	2.69949 × 10^0^	2.36537 × 10^4^	3.20335 × 10^0^	2.65856 × 10^0^	2.90962 × 10^0^	2.90962 × 10^0^	2.65856 × 10^0^	2.65856 × 10^0^	2.90322 × 10^0^
	Best	2.65856 × 10^0^	2.65856 × 10^0^	2.65856 × 10^0^	2.65856 × 10^0^	2.67240 × 10^0^	2.65856 × 10^0^	2.65856 × 10^0^	2.65856 × 10^0^	3.37600 × 10^0^	2.65856 × 10^0^	2.65856 × 10^0^	2.65856 × 10^0^	2.65856 × 10^0^	2.65856 × 10^0^	2.65856 × 10^0^	2.65856 × 10^0^
	Rank	1	2	10	6	15	14	13	7	16	12	3	8	11	4	5	9
RW22	Mean	-3.06655 × 10^4^	-3.06655 × 10^4^	-3.06655 × 10^4^	-3.06655 × 10^4^	-3.06342 × 10^4^	-3.05123 × 10^4^	-3.06655 × 10^4^	-3.06655 × 10^4^	-2.87550 × 10^4^	-3.06655 × 10^4^	-3.06655 × 10^4^	-3.06655 × 10^4^	-3.06655 × 10^4^	-3.06655 × 10^4^	-3.06655 × 10^4^	-3.06655 × 10^4^
	Std	1.11005 × 10^−11^	1.09973 × 10^−11^	1.11005 × 10^−11^	1.11005 × 10^−11^	1.41461 × 10^1^	6.83088 × 10^1^	1.11005 × 10^−11^	1.11005 × 10^−11^	5.95149 × 10^2^	1.88067 × 10^−11^	2.15120 × 10^−11^	1.11005 × 10^−11^	4.01721 × 10^−5^	1.11005 × 10^−11^	1.11005 × 10^−11^	1.11005 × 10^−11^
	Worst	-3.06655 × 10^4^	-3.06655 × 10^4^	-3.06655 × 10^4^	-3.06655 × 10^4^	-3.06008 × 10^4^	-3.03490 × 10^4^	-3.06655 × 10^4^	-3.06655 × 10^4^	-2.71913 × 10^4^	-3.06655 × 10^4^	-3.06655 × 10^4^	-3.06655 × 10^4^	-3.06655 × 10^4^	-3.06655 × 10^4^	-3.06655 × 10^4^	-3.06655 × 10^4^
	Best	-3.06655 × 10^4^	-3.06655 × 10^4^	-3.06655 × 10^4^	-3.06655 × 10^4^	-3.06581 × 10^4^	-3.06316 × 10^4^	-3.06655 × 10^4^	-3.06655 × 10^4^	-2.98151 × 10^4^	-3.06655 × 10^4^	-3.06655 × 10^4^	-3.06655 × 10^4^	-3.06655 × 10^4^	-3.06655 × 10^4^	-3.06655 × 10^4^	-3.06655 × 10^4^
	Rank	1	1	1	1	14	15	1	1	6	12	11	1	13	1	1	1
RW23	Mean	-6.09308 × 10^3^	-6.03948 × 10^3^	-6.18638 × 10^3^	-6.01653 × 10^3^	-5.20984 × 10^3^	-5.31168 × 10^3^	-6.01186 × 10^3^	-5.98941 × 10^3^	-9.26845 × 10^2^	-5.79754 × 10^3^	-5.74301 × 10^3^	-6.13009 × 10^3^	-5.65363 × 10^3^	-4.96078 × 10^3^	-6.00824 × 10^3^	-6.16138 × 10^3^
	Std	4.48731 × 10^1^	8.18332 × 10^1^	4.46849 × 10^1^	1.38671 × 10^2^	8.83264 × 10^1^	2.01142 × 10^2^	9.00723 × 10^1^	6.66196 × 10^1^	1.38057 × 10^4^	1.24820 × 10^2^	1.17643 × 10^2^	4.38905 × 10^1^	1.68287 × 10^2^	1.74342 × 10^2^	3.77817 × 10^1^	3.75770 × 10^1^
	Worst	-5.98819 × 10^3^	-5.91355 × 10^3^	-6.08670 × 10^3^	-5.73686 × 10^3^	-5.06550 × 10^3^	-4.92188 × 10^3^	-5.81720 × 10^3^	-5.85716 × 10^3^	6.30904 × 10^4^	-5.53580 × 10^3^	-5.50542 × 10^3^	-6.05006 × 10^3^	-5.24285 × 10^3^	-4.73308 × 10^3^	-5.92532 × 10^3^	-6.05925 × 10^3^
	Best	-6.16896 × 10^3^	-6.23012 × 10^3^	-6.25535 × 10^3^	-6.23423 × 10^3^	-5.40446 × 10^3^	-5.68833 × 10^3^	-6.19134 × 10^3^	-6.12953 × 10^3^	-4.99514 × 10^3^	-6.09053 × 10^3^	-5.98628 × 10^3^	-6.20593 × 10^3^	-5.94794 × 10^3^	-5.30657 × 10^3^	-6.08592 × 10^3^	-6.22475 × 10^3^
	Rank	4	5	1	6	14	13	7	9	16	10	11	3	12	15	8	2
RW24	Mean	8.50671 × 10^−1^	8.58069 × 10^−1^	1.02724 × 10^4^	7.93669 × 10^2^	5.45453 × 10^0^	1.14006 × 10^3^	3.28924 × 10^−1^	9.09933 × 10^−1^	2.30542 × 10^3^	1.51046 × 10^2^	4.60362 × 10^2^	8.45188 × 10^−1^	1.31510 × 10^0^	4.03141 × 10^−1^	6.25687 × 10^−1^	9.46842 × 10^−1^
	Std	1.17300 × 10^−1^	2.41755 × 10^−1^	1.99793 × 10^4^	3.64238 × 10^3^	6.36852 × 10^0^	8.99922 × 10^2^	1.52274 × 10^−1^	1.78431 × 10^−1^	1.08781 × 10^3^	8.22965 × 10^2^	1.50287 × 10^3^	3.31914 × 10^−1^	2.03150 × 10^0^	1.09215 × 10^−1^	2.28690 × 10^−1^	3.23357 × 10^−1^
	Worst	1.01563 × 10^0^	1.16678 × 10^0^	7.80474 × 10^4^	1.96616 × 10^4^	3.23716 × 10^1^	2.71888 × 10^3^	7.29312 × 10^−1^	1.15685 × 10^0^	3.20014 × 10^3^	4.50836 × 10^3^	6.79647 × 10^3^	1.73101 × 10^0^	1.20701 × 10^1^	6.01387 × 10^−1^	1.44417 × 10^0^	2.02329 × 10^0^
	Best	6.02245 × 10^−1^	5.26921 × 10^−1^	4.59271 × 10^−1^	2.77477 × 10^−1^	1.26240 × 10^0^	1.05249 × 10^1^	1.52510 × 10^−1^	3.19896 × 10^−1^	8.65624 × 10^1^	3.94265 × 10^−1^	2.72299 × 10^−1^	3.15638 × 10^−1^	9.36777 × 10^−1^	1.62039 × 10^−1^	2.84187 × 10^−1^	4.32804 × 10^−1^
	Rank	5	6	16	13	10	14	1	7	15	11	12	4	9	2	3	8
RW25	Mean	2.43709 × 10^−1^	4.28259 × 10^−1^	1.37621 × 10^4^	1.04734 × 10^3^	5.80175 × 10^0^	4.07007 × 10^1^	2.35910 × 10^−1^	7.20430 × 10^2^	4.31219 × 10^3^	7.01939 × 10^1^	2.24322 × 10^1^	2.96562 × 10^−1^	2.16056 × 10^3^	5.30635 × 10^−1^	3.12812 × 10^−1^	3.09545 × 10^1^
	Std	4.55225 × 10^−2^	8.42715 × 10^−2^	1.70173 × 10^4^	3.42034 × 10^3^	3.58609 × 10^0^	1.40426 × 10^2^	1.10241 × 10^−1^	1.46475 × 10^3^	2.13770 × 10^3^	2.78862 × 10^2^	1.20440 × 10^2^	1.47741 × 10^−1^	1.79371 × 10^3^	1.50557 × 10^−1^	9.35484 × 10^−2^	1.67485 × 10^2^
	Worst	3.03659 × 10^−1^	5.07096 × 10^−1^	6.29204 × 10^4^	1.37352 × 10^4^	1.42809 × 10^1^	7.82640 × 10^2^	5.30746 × 10^−1^	3.60074 × 10^3^	6.39933 × 10^3^	1.37009 × 10^3^	6.60120 × 10^2^	5.70409 × 10^−1^	3.60059 × 10^3^	8.43506 × 10^−1^	6.84027 × 10^−1^	9.17728 × 10^2^
	Best	1.52570 × 10^−1^	2.77042 × 10^−1^	1.29766 × 10^−1^	1.80882 × 10^−1^	5.25403 × 10^−1^	5.17096 × 10^−1^	9.37703 × 10^−2^	1.82331 × 10^−1^	4.91980 × 10^2^	1.41750 × 10^−1^	1.81228 × 10^−1^	1.12564 × 10^−1^	4.25116 × 10^−1^	2.99254 × 10^−1^	2.10231 × 10^−1^	1.57224 × 10^−1^
	Rank	2	6	16	13	8	10	1	12	15	11	9	3	14	7	5	4
RW26	Mean	1.61748 × 10^−1^	6.54022 × 10^−1^	1.71772 × 10^4^	1.82397 × 10^3^	2.81601 × 10^1^	3.04783 × 10^3^	2.66744 × 10^−1^	1.07230 × 10^2^	8.83488 × 10^3^	5.85562 × 10^−1^	2.14899 × 10^2^	5.18554 × 10^−1^	4.28762 × 10^−1^	4.80015 × 10^−1^	5.37973 × 10^−1^	4.86457 × 10^−1^
	Std	4.99415 × 10^−2^	2.38577 × 10^−1^	2.81303 × 10^4^	5.13314 × 10^3^	1.49512 × 10^1^	2.42241 × 10^3^	1.52337 × 10^−1^	2.76597 × 10^2^	2.98941 × 10^3^	2.56753 × 10^−1^	8.36403 × 10^2^	2.43099 × 10^−1^	2.89386 × 10^−1^	1.38757 × 10^−1^	2.49998 × 10^−1^	2.15119 × 10^−1^
	Worst	2.65797 × 10^−1^	1.06148 × 10^0^	1.11749 × 10^5^	2.54643 × 10^4^	6.01774 × 10^1^	1.08066 × 10^4^	8.76187 × 10^−1^	8.00563 × 10^2^	1.07999 × 10^4^	1.25383 × 10^0^	3.91780 × 10^3^	1.14474 × 10^0^	1.80152 × 10^0^	7.68054 × 10^−1^	8.94458 × 10^−1^	8.76283 × 10^−1^
	Best	1.05829 × 10^−1^	2.83526 × 10^−1^	1.11310 × 10^−1^	2.00714 × 10^−1^	3.50973 × 10^0^	1.20983 × 10^1^	1.19210 × 10^−1^	1.48502 × 10^−1^	8.00592 × 10^2^	2.52660 × 10^−1^	2.37610 × 10^−1^	1.60871 × 10^−1^	1.46447 × 10^−1^	2.28773 × 10^−1^	2.16611 × 10^−1^	1.35419 × 10^−1^
	Rank	1	9	16	13	10	14	2	11	15	8	12	6	3	4	7	5
RW27	Mean	2.89975 × 10^−1^	1.11639 × 10^2^	2.72654 × 10^4^	8.94331 × 10^3^	3.29824 × 10^0^	2.48858 × 10^1^	8.45449 × 10^1^	1.74870 × 10^3^	1.50766 × 10^4^	8.15295 × 10^3^	8.80463 × 10^3^	1.65614 × 10^1^	3.33544 × 10^3^	1.55639 × 10^2^	2.88096 × 10^−1^	8.60569 × 10^1^
	Std	1.69410 × 10^−1^	6.08920 × 10^2^	2.43298 × 10^4^	1.30199 × 10^4^	2.18031 × 10^0^	1.24512 × 10^1^	4.61167 × 10^2^	2.07958 × 10^3^	2.63283 × 10^3^	3.50768 × 10^4^	1.81347 × 10^4^	8.83423 × 10^1^	5.03791 × 10^−2^	3.78091 × 10^2^	1.26665 × 10^−1^	4.52095 × 10^2^
	Worst	6.03700 × 10^−1^	3.33566 × 10^3^	7.13189 × 10^4^	5.38429 × 10^4^	1.00682 × 10^1^	4.30883 × 10^1^	2.52626 × 10^3^	6.66521 × 10^3^	1.66653 × 10^4^	1.92821 × 10^5^	7.95030 × 10^4^	4.84303 × 10^2^	3.33553 × 10^3^	1.52262 × 10^3^	6.85854 × 10^−1^	2.47809 × 10^3^
	Best	1.26331 × 10^−1^	3.29680 × 10^−1^	2.35184 × 10^−1^	3.78929 × 10^−1^	7.91455 × 10^−1^	1.43674 × 10^0^	2.40017 × 10^−1^	2.40564 × 10^−1^	7.32799 × 10^3^	1.58643 × 10^−1^	2.28415 × 10^−1^	2.19139 × 10^−1^	3.33540 × 10^3^	1.75852 × 10^−1^	1.32298 × 10^−1^	1.60279 × 10^−1^
	Rank	2	8	16	14	3	5	6	10	15	12	13	4	11	9	1	7
Mean Rank		2.19	3.74	6.89	7.30	12.89	13.67	5.67	5.44	15.37	9.81	10.96	5.30	9.19	7.70	3.33	5.89

## Data Availability

Data will be made available upon request.

## Supplementary Materials

The following supporting information can be downloaded at: https://www.mdpi.com/article/10.3390/biomimetics11100764/s1, Table S1: Comparison of linear and nonlinear convergence factors; Table S2: CEC 2020 test suite. Table S3: 27 real-world engineering optimization cases.
