# Supplementary material for: SPNOA: An Improved Nutcracker Optimizer Based on Specular Reflection Learning and Probability-Driven Hybrid Mutation Strategy for Global Optimization
Source: Biomimetics (Basel). 2026 Oct 21;11(10):764. doi: 10.3390/biomimetics11100764 (PMC13702218; doi:10.3390/biomimetics11100764)
Supplement: Supplementary file 1 [file biomimetics-11-00764-s001.zip › biomimetics-4534620-supplementary.pdf]

Supplementary Material

Table S1. Comparison of linear and nonlinear convergence factors.

Fun	Linear	2nd power	4th power	6th power
F1	1.00000E+02	**1.00000E+02**	1.00000E+02	1.00000E+02
F2	3.01805E+03	2.99476E+03	3.05518E+03	**2.89897E+03**
F3	7.85190E+02	7.84441E+02	**7.78565E+02**	7.85122E+02
F4	**1.90000E+03**	**1.90000E+03**	**1.90000E+03**	**1.90000E+03**
F5	2.23621E+03	2.44534E+03	2.53243E+03	**2.23137E+03**
F6	1.67639E+03	1.68446E+03	**1.66815E+03**	1.68115E+03
F7	2.31953E+03	2.34184E+03	2.30771E+03	**2.29417E+03**
F8	2.30020E+03	2.30025E+03	**2.30008E+03**	2.30051E+03
F9	2.89060E+03	2.89354E+03	**2.88450E+03**	2.89448E+03
F10	2.88884E+03	2.88727E+03	**2.88610E+03**	2.88669E+03
Mean Rank	2.7	2.6	**1.9**	2.2

Table S2. CEC 2020 test suite.

Prob	No.	Functions	$F_i^* = F_i(x^*)$
Unimodal Function	1	Shifted and Rotated Bent Cigar Function	100
	2	Shifted and Rotated Schwefel's Function	1100
Basic Functions	3	Shifted and Rotated Lunacek bi-Rastrigin Function	700
	4	Expanded Rosenbrock's plus Griewangk's Function	1900
	5	Hybrid Function 1 ($N = 3$)	1700
Hybrid Functions	6	Hybrid Function 2 ($N = 4$)	1600
	7	Hybrid Function 3 ($N = 5$)	2100
	8	Composition Function 1 ($N = 3$)	2200
Composition Functions	9	Composition Function 2 ($N = 4$)	2400
	10	Composition Function 3 ($N = 5$)	2500
		Search range: $[-100, 100]^D$	

Table S3. 27 real-world engineering optimization cases.

Prob	Name	*D*	*g*(*z*)	*h*(*z*)	*f**(*z*)
RW01	Heat exchanger network (case 1)	9	0	8	1.8931162966E+02
RW02	Heat exchanger network (case 2)	11	0	9	7.0490369540E+03
RW03	Alkylation unit	7	14	0	-4.5291197395E+03
RW04	Process synthesis	2	2	0	2.0000000000E+00
RW05	Process synthesis and design	3	1	1	2.5576545740E+00
RW02	Process flow sheeting	3	3	0	1.0765430833E+00
RW07	Process synthesis	7	9	0	2.9248305537E+00
RW08	Process design	5	3	0	2.6887000000E+04
RW09	Multi-product batch plant	10	10	0	5.3638942722E+04
RW10	Speed reducer	7	11	0	2.9944244658E+03
RW11	Industrial refrigeration system	14	15	0	3.2213000814E-02
RW12	Spring design (case 1)	3	3	0	1.2665232788E-02
RW13	Pressure vessel	4	4	0	5.8853327736E+03
RW14	Welded beam	4	5	0	1.6702177263E+00
RW15	Three-bar truss design	2	3	0	2.6389584338E+02
RW16	Multiple disk clutch brake design	5	7	0	2.3524245790E-01
RW17	Step-cone pulley problem	5	8	3	1.6069868725E+01
RW18	Robot gripper problem	7	7	0	2.5287918415E+00
RW19	10-bar truss design	10	3	0	5.2445076066E+02
RW20	Rolling element bearing	10	9	0	1.4614135715E+04
RW21	Spring design (case 2)	3	8	0	2.6138840583E+00
RW22	Himmelblau's function	5	6	0	-3.0665538672E+04
RW23	Wind farm layout problem	30	91	0	-6.2731715000E+03
RW24	SOPWM for 3-level inverters	25	24	1	3.0739360000E-02
RW25	SOPWM for 5-level inverters	25	24	1	2.0240335000E-02
RW26	SOPWM for 7-level inverters	25	24	1	1.2783068000E-02
RW27	SOPWM for 11-level inverters	30	29	1	9.3118741800E-03
